# Graphdiyne biomaterials: from characterization to properties and applications

**DOI:** 10.1186/s12951-025-03227-y

**Published:** 2025-03-04

**Authors:** Ling-Xiao Zhao, Yong-Gang Fan, Xue Zhang, Chan Li, Xue-Yan Cheng, Feng Guo, Zhan-You Wang

**Affiliations:** 1 Key Laboratory of Medical Cell Biology of Ministry of Education, Key Laboratory of Major Chronic Diseases of Nervous System of Liaoning Province, Health Sciences Institute of China Medical University, Shenyang, 110122 China; 2 Central Laboratory, Cancer Hospital of Dalian University of Technology, Liaoning Cancer Hospital & Institute, Shenyang, 110042 China; 3 Department of Pharmacy, The Fourth Affiliated Hospital of China Medical University, Shenyang, 110032 China; 4 Department of Pharmaceutical Toxicology, School of Pharmacy, China Medical University, Shenyang, 110122 China

**Keywords:** Graphdiyne, Characterization techniques, Nanodelivery system, Biomedical application, Antitumor

## Abstract

Graphdiyne (GDY), the sole synthetic carbon allotrope with *sp*-hybridized carbon atoms, has been extensively researched that benefit from its pore structure, fully conjugated surfaces, wide band gaps, and more reactive C≡C bonds. In addition to the intrinsic features of GDY, engineering at the nanoscale, including metal/transition metal ion modification, chemical elemental doping, and other biomolecular modifications, endowed GDY with a broader functionality. This has led to its involvement in biomedical applications, including enzyme catalysis, molecular assays, targeted drug delivery, antitumor, and sensors. These promising research developments have been made possible by the rational design and critical characterization of GDY biomaterials. In contrast to other research areas, GDY biomaterials research has led to the development of characterization techniques and methods with specific patterns and some innovations based on the integration of materials science and biology, which are crucial for the biomedical applications of GDY. The objective of this review is to provide a comprehensive overview of the biomedical applications of GDY and the characterization techniques and methods that are essential in this process. Additionally, a general strategy for the biomedical research of GDY will be proposed, which will be of limited help to researchers in the field of GDY or nanomedicine.

**Graphical Abstract:**

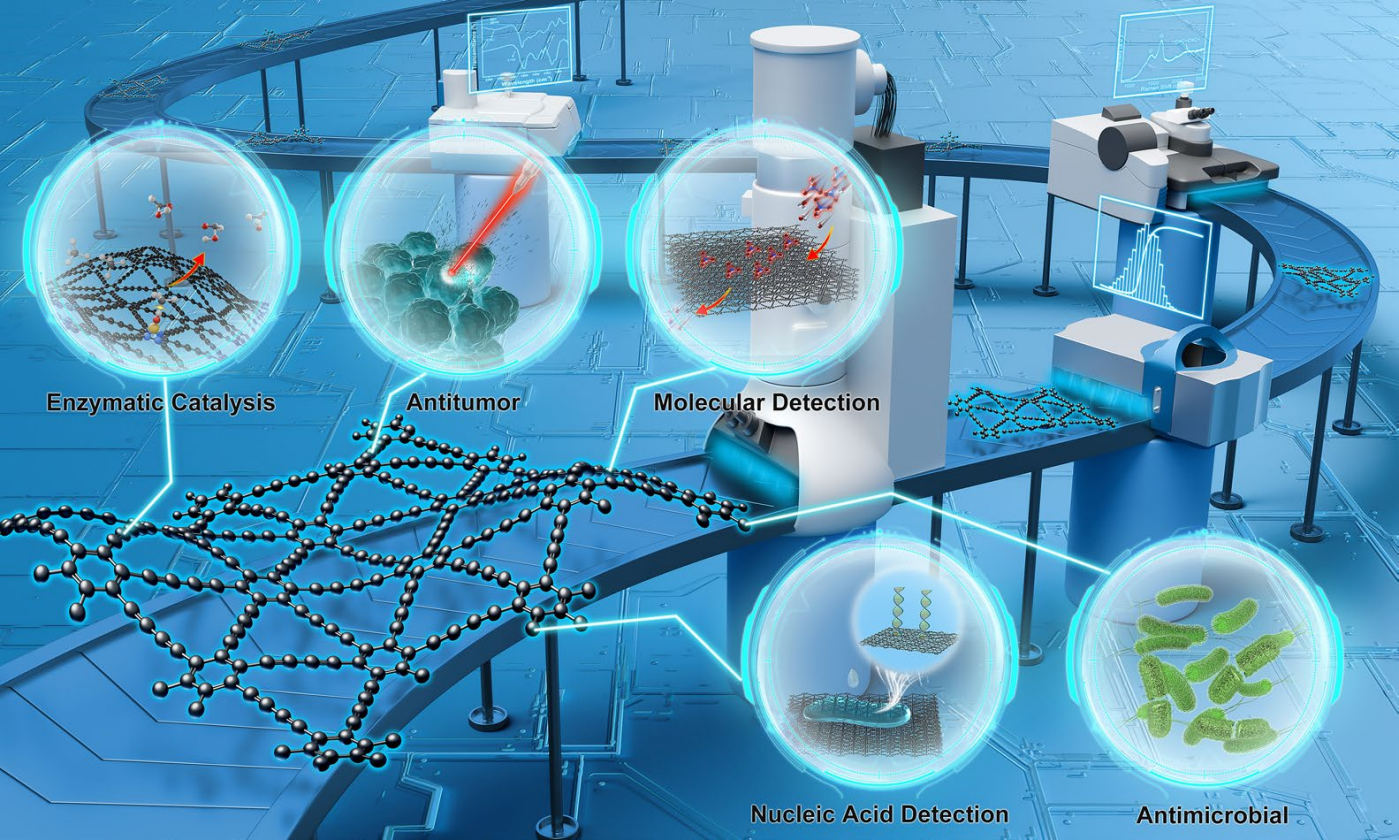

**Supplementary Information:**

The online version contains supplementary material available at 10.1186/s12951-025-03227-y.

## Introduction

Graphdiyne (GDY), the sole recently synthesized carbon isomer with *sp*-hybridized carbon atoms [1, 2], has demonstrated excellent properties in several fields, including energy storage, catalysis, electronics, and photovoltaic conversion. These properties are attributed to its wider natural band gap, fully-conjugated structure, and higher activity of *sp*-hybridized carbon atoms [3–5]. The rapid development of nanoscience has enabled the exploration of a wider range of application scenarios in materials science, which has led researchers to divert their attention to biomedical applications of materials [6]. Similar to but distinct from graphene, the structural characteristics of GDY permit it to exhibit unique properties and considerable potential in biomedical applications [7].

The application of materials in biomedicine generally follows a progression from laboratory to clinical settings, from in vitro to in vivo studies, and from non-invasive to invasive procedures [8, 9]. With the synthesis and performance testing of GDY and its variants, GDY nanomaterials have begun to expand from enzyme catalysis and molecular detection applications to drug delivery, cancer therapy, and in vivo imaging studies [10–13]. For instance, GDY has been employed extensively to fabricate catalytically active GDY/metal composites, due to its distinctive C≡C structure, which endows it with a strong affinity for transition metal ions and heavy metal ions [14–16]. Furthermore, GDY’s natural band gap, substantial specific surface area, and conjugation system enable it to adsorb polycyclic aromatic hydrocarbons and exhibit remarkable drug-carrying capacity [17–19]. Moreover, GDY’s exceptional extinction coefficient across a broad wavelength range and its robust light absorption in the near-infrared region (NIR) confer upon it remarkable photothermal conversion capability, which are being explored for potential applications in photothermal therapy (PTT) [20–22]. These properties of GDY are the reason for its rapid development in biomedical research. However, this advanced cross-disciplinary progress has in turn made biomedical research of materials, including GDY, more challenging.

It can be observed that biomaterials research, which encompasses not only GDY biomaterials, frequently employs characterization techniques and methodologies [23]. However, based on the previously reported work, we believe that conducting GDY biomaterials research by referring to other 2-dimensional (2D) materials or previous reports on GDY, although feasible, makes it difficult for both researchers and readers to gain a deep understanding of the rationale and significance of the characterization methodology. This, in turn, prevents some of the studies from providing comprehensive data on GDY biomaterials, leading to a lack of detail in interpreting the parameters of the GDY biomaterials and creating problems for subsequent work [24, 25].

In this review, we highlighted several types of characterization techniques that have been used in GDY biomedical applications, including morphological observations, modification analysis, and property testing by analyzing specific studies. Electron microscopy is the most common method for obtaining morphology images of GDY nanomaterials. These images are used to determine parameters such as the structure, size, lattice, and thickness of the nanomaterials [26–28]. Furthermore, characterization methods for the modification of GDY include Energy dispersive spectroscopy (EDS), X-ray photoelectron spectroscopy (XPS), X-ray diffraction (XRD), Raman spectroscopy, and Fourier transform infrared spectroscopy (FTIR) are widely used [24, 29–32]. It is important to note that the data generated by each characterization technique are not independent of one another. Therefore, researchers must analyze these data collectively in order to ultimately reveal the parameters and properties of GDY materials.

The potential application of GDY in biomedicine is a fascinating research frontier. Advanced development of GDY biomaterials and detailed testing of their structures, parameters, and properties are expected to expand the application landscape of GDY materials. Furthermore, by conducting a comprehensive evaluation of the functionality and biosafety of GDY biomaterials, it is anticipated that the feasibility of GDY materials for biomedical applications will be enhanced, thereby facilitating the development of more effective disease treatment strategies [33, 34]. By emphasizing the characterization techniques and research strategies of GDY biomaterials, supplemented by detailed case studies, this review will assist researchers in comprehending and conducting GDY biomedical research in a more comprehensive, detailed, and in-depth manner.

## Structure and properties of GDY

### Structure of GDY

**Fig. 1.**
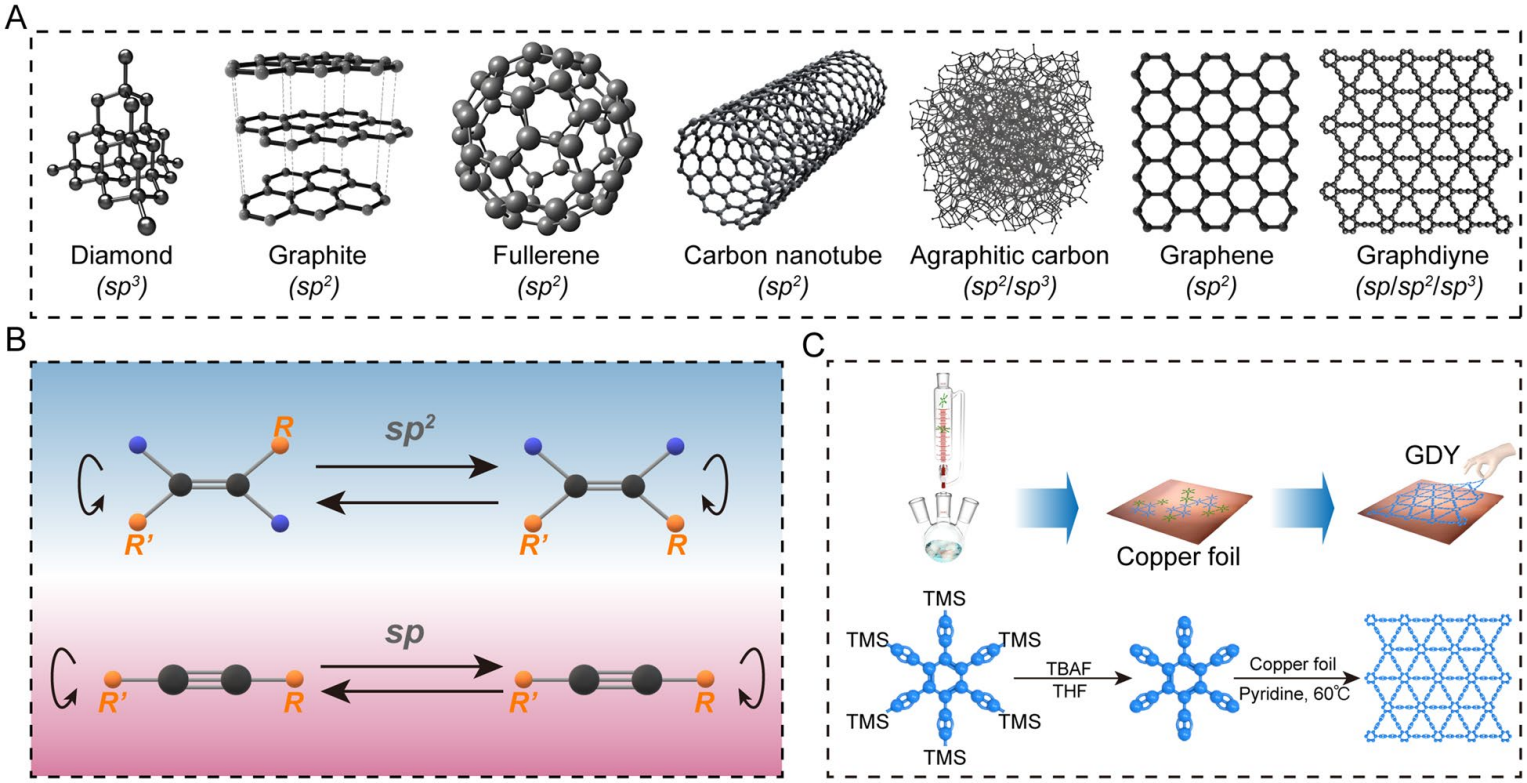
Carbon isoforms and GDY synthesis modes. **A** Structures of naturally derived or artificially synthesized carbon isoforms. **B** C≡C does not have the cis–trans structure of C=C. **C** Schematic diagram of the GDY preparation by coupling hexynylbenzene using Cu foil as catalyst and substrate.

The development of carbon materials has undergone a process of transition from naturally occurring to synthetic. Carbon materials in their natural state are primarily composed of carbon atoms in *sp*^*2*^- (graphite) or *sp*^*3*^- (diamond) hybridized forms (Fig. 1A) [35–40]. Novoselov et al. employed *sp*^*2*^-hybridized orbitals of carbon atoms to synthesize graphene, a planar 2D material with a hexagonal honeycomb lattice. This innovation initiated a new paradigm in graphene materials research and gradually facilitated its practical applications [41–43]. Nevertheless, although the *sp*-hybridized form of C≡C is more likely to form stable planar structures devoid of cis–trans isomers and with a higher degree of electron conjugation, the lack of a viable synthetic process has prevented a breakthrough in the preparation of *sp*-hybridized carbon materials (Fig. 1B).

In 1968, Baughman RH et al. derived a novel graphite structure, designated GDY, which contains exclusively *sp-* and *sp*^*2*^*-*hybridized carbon [44, 45]. Subsequent studies demonstrated that the deprotection of tetraethynyl derivatives by copper (Cu)-catalyzed oxidation yielded diynyl carbon isomers. However, the instability of the deprotected polyalkyne led to significant limitations in the synthesis of GDY [46]. In 2004, Coluci et al. developed alkyne-containing graphite nanotubes by introducing alkyne groups to prolong the covalent links of graphite-based nanotubes [44]. In 2008, Haley MM optimized an existing process and synthesized Graphyne and GDY, which were based on dehydrobenzocyclohexene and dehydrobenzocyclohexene skeletons, respectively [47]. Until 2010, Li et al. synthesized hexaethynylbenzene monomer by adding tetrabutylammonium fluoride (TBAF) to the tetrahydrofuran (THF) solution of hexakis[(trimethylsilyl)ethynyl]benzene, and prepared large-area GDY with 2D structural properties by cross-coupling the hexaethynylbenzene monomer under nitrogen (N) and pyridine conditions at 60 ℃ for 72 h with copper foil as the catalyst and the substrate, which has been an innovation, and has been from the theoretical to experimental testing since (Fig. 1C) [2].

**Fig. 2:**
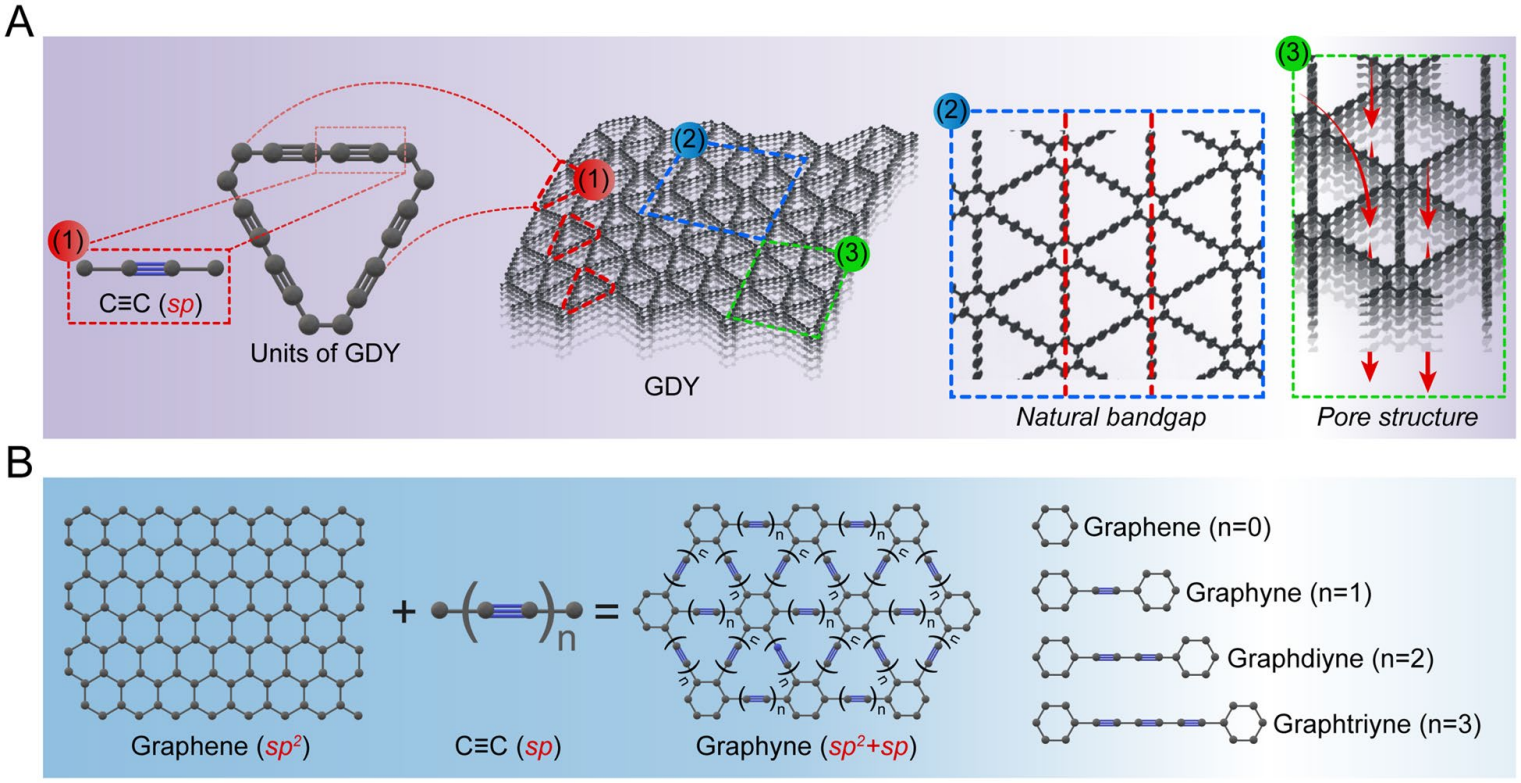
Structure characterization of GDY. **A** Large triangular basic unit (1), natural band gap (2) and pore structure (3) of GDY. **B** Schematic diagram of graphene connecting aromatic groups to GDY by adding linear acetylene and classification of GDY

GDY is a single-atom-layer 2D all-carbon polymer formed by linking benzene rings by 1,3-diyne bonds. Structurally, GDY can be viewed as one-third of C–C in graphene with different numbers of C≡C bonds inserted into it. This results in not only the benzene ring, but also a large triangular ring with 18 carbon atoms consisting of benzene ring and C≡C bonds, which forms the basic repeating unit of GDY (Fig. 2). GDY exhibits the properties of a conventional 2D material, where folds are formed to varying degrees on top of the planar structure in order to maintain its own structural stability. The 3-dimensional (3D) GDY structure is formed between GDY molecules through Van der Waals forces and π–π stacking. Large triangular structures are stacked in GDY molecules to form pores, which results in more carbon bonds, stronger conjugation systems, and a homogeneous pore structure (Fig. 2A). This allows for better adsorption of molecules and loading of ligands [1].

### Property of GDY

**Fig. 3:**
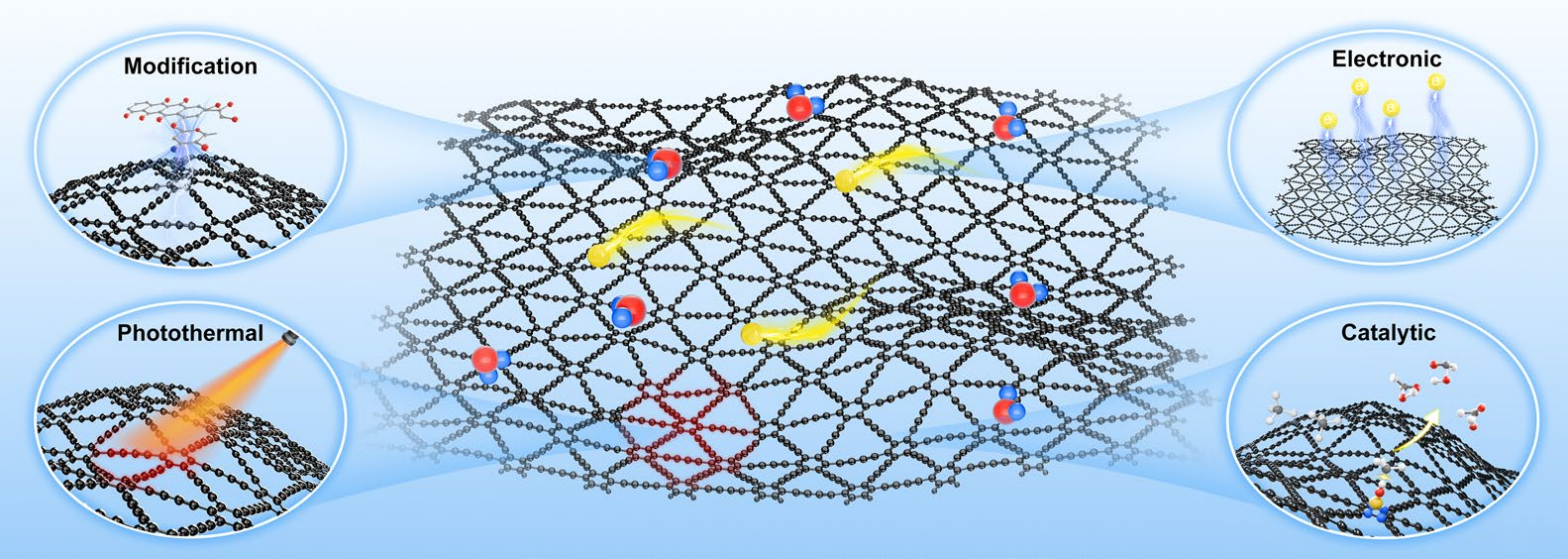
Properties of GDY. GDY has intrinsic electrical, catalytic, photothermal and modification friendly properties

Due to its considerable specific surface area, fully conjugated structure, intrinsic band gap, inherent pore structure, capacity for light absorption, and the presence of highly active *sp*-hybridized carbon atoms, GDY has demonstrated remarkable efficiency in energy storage, catalysis, electronics, and photovoltaic conversion (Fig. 3) [3–5].

#### Electrical property

First Principles studies have demonstrated that GDY contains a natural band gap and pore structure (Fig. 2A), making it a class of intrinsic semiconductors with superior charge transport capability [48]. In optoelectronics research, Jin et al. prepared self-assembled films based on GDY and zinc oxide (ZnO) nanoparticles. These films exhibited significantly enhanced photoresponsiveness and performance compared with conventional UV photodetectors [49]. Xiao et al. employed the high charge transport ability and excellent semiconductor properties of GDY in the development of chalcogenide solar cells, resulting in an average photoelectric conversion efficiency increase of 20% and a notable improvement in the stability of the prepared cell devices [50]. In 2015, Ren et al. prepared a complex of GDY and platinum (Pt) nanoparticles (GDY/Pt), which greatly improved its catalytic activity and electron transport ability. Furthermore, this GDY/Pt nanoparticle complex also significantly improved the energy conversion efficiency of dye-sensitized solar cells [51]. Moreover, the reduced atomic density of the alkyne bonds in GDY, as well as the porous nature of 3D GDY (Fig. 2A) allows it to accommodate a substantial quantity of lithium ions, theoretically adsorbing lithium at twice the capacity of graphite [52, 53]. In a recent study, for the first time, GDY membranes of varying thicknesses were prepared and assembled into batteries, resulting in high lithium storage performance under laboratory conditions [54].

#### Catalytic property

GDY and its derivatives have the potential to serve as a catalyst, which strongly depends on the structure of GDY itself and the properties of the modifiers [55–59]. Wu P et al. demonstrated through theoretical calculations that GDY can catalyze carbon monoxide at low temperatures benefiting from its *sp*-hybridized carbon atoms and is a metal-free redox electrocatalyst [60]. And the catalytic properties exhibited by GDY composites, attributable to the incorporation of modifiers, were also attributed to the high activity of the *sp*-hybridized carbon atoms of GDY [61–63]. In a study by Zhang et al. GDY crosslinked with graphene oxide (GO) and hybridized with silver/silver (Ag/Ag) bromide (GDY/GO/Ag/Ag), was used for the degradation of methyl orange pollutants under visible light. The results demonstrated that the degradation of methyl orange pollutants by GDY/GO/Ag/Ag bromide complexes was significantly enhanced [61]. Yang NL et al. demonstrated that titanium dioxide (TiO_2_)-GDY (TiO_2_-GDY) complex exhibited a greater oxidizing capacity than TiO_2_ and TiO_2_-graphene complex, which degraded methylene blue at a rate 1.63 times higher than that of TiO_2_ [62]. The composites prepared by Thangavel S et al. comprising GDY and ZnO nanoparticles demonstrated effective degradation of azo-endo dyes [63]. The aforementioned studies have demonstrated that GDY and its derived materials exhibit excellent catalytic properties.

#### Photothermal property

The superior extinction coefficient of GDY in a wide range of wavelengths and its strong light absorption in NIR, which, combined with its enormous surface area, result in excellent photothermal conversion properties [25, 64]. Previous studies on GDY biomaterials have demonstrated that polyethylene glycol (PEG)-modified GDY (GDY-PEG) can be stably dispersed in physiological solutions, with a photothermal conversion efficiency of up to 42%. This efficiency is higher than that of most carbon materials, suggesting that GDY-PEG has the potential to be a high-efficiency photothermal conversion agent [65]. Jin et al. developed a drug delivery system based on GDY nanosheets using doxorubicin (DOX). This system took advantage of the photothermal properties of GDY, which synergistically inhibited tumor growth of mice under 808 nm irradiation through heat production [25].

#### Doping and modification friendly

Due to the intrinsic properties of its *sp*-hybridized carbon atoms, the highly active carbon–carbon triple bond and fully conjugated structure, GDY are easier to be elementally doped and modified [66, 67]. Theoretical calculations indicated that GDY doped with Boron (B) and N atoms can stabilize the conformation and modulate the band gap [68]. In addition, the B-doped GDY embedded lithium atoms to achieve excellent hydrogen storage performance [69]. Wang et al. provided a more comprehensive review of the precise modification techniques of GDY, such as utilizing metal-GDY bonding to enhance charge transfer between materials, using the chemical reaction sites conferred by alkyne bonding to achieve fixed-point controllable doping of heterogeneous atoms, and utilizing the interaction of GDY alkyne-bonded electrons with the metal null orbitals to regulate the transport and anchoring of ions or atoms [70]. Consequently, the simplicity of doping and modifying GDY provides a priori opportunities for its investigation and utilisation in the fields of electronics, energy storage, and, in particular, biomedical applications.

## Modifications to GDY

The modification of GDY can be broadly classified into two categories: covalent modification and non-covalent modification. The *sp*- and *sp*^*2*^-hybridized carbon atoms of GDY exhibit a strong adsorption capacity for Hydrogen (H), Fluorine (F), N, Oxygen (O), and other atoms. This property can be exploited to covalently modify the unsaturated carbon atoms present in GDY [66]. In consideration of the various doping elements and binding modes, noncovalent modification is divided into noncovalent adsorption and metal ion doping. The former is primarily attributable to the substantial specific surface area and distinctive conjugation system of GDY, which enables the adsorption of polycyclic aromatic hydrocarbons [17–19]. In parallel, the distinctive C≡C structure of GDY exhibits a profound affinity for transition metal ions. This attribute enables GDY to be modified by transition metal ions or nanoparticles, which, in turn, enhances the physicochemical properties of GDY and introduces novel characteristics [14–16].

### Covalent modification of GDY

The unsaturated carbon atoms in GDY can be covalently modified to introduce heteroatoms, thereby enhancing the surface chemical activity of GDY nanomaterials [71–74]. Zhang et al. employed a heating process under argon conditions to react GDY with ammonia, thereby obtaining N-doped GDY. The N-doped GDY exhibited a high degree of active site generation, which resulted in superior performance in lithium-ion batteries [75]. Additionally, theoretical simulations have demonstrated that fluorinated GDY exhibits superior thermal stability [76]. The researchers additionally addressed the impact of N, B, Phosphorus (P), and Sulfur (S) doping of graphyne, and conducted a theoretical analysis of the catalytic performance of redox reactions on graphyne nanotubes (GNTs), which revealed that N-doped GNT exhibited the highest catalytic activity, followed by B- doped GNT, P- doped GNT, and S- doped GNT [77]. Based on these findings, Kang et al. demonstrated that multi-element doping can effectively modulate the GDY optoelectronic properties. For instance, the doping of three elements, B, N, and O, played a pivotal role in modulating the GDY electrical properties [78]. Oxygen doping is a common method of improving the capacity of materials. The impact of edge-oxidized GDY materials on lithium atom storage was examined by modifying GDY with distinct oxygen functional groups. The findings indicated that the GDY materials with carboxyl functional groups introduced at the edges exhibited the most favorable chemical stability [79]. In contrast, Wang CX et al. employed the technique of strong acid oxidation to modify oxygen-containing functional groups at the edge of GDY, resulting in the synthesis of graphdiyne oxide (GDYO) with excellent dispersibility in aqueous solution. This provides a solid foundation for further investigation of GDY materials in the biomedical field [80].

### Non-covalent modifications of GDY

**Fig. 4:**
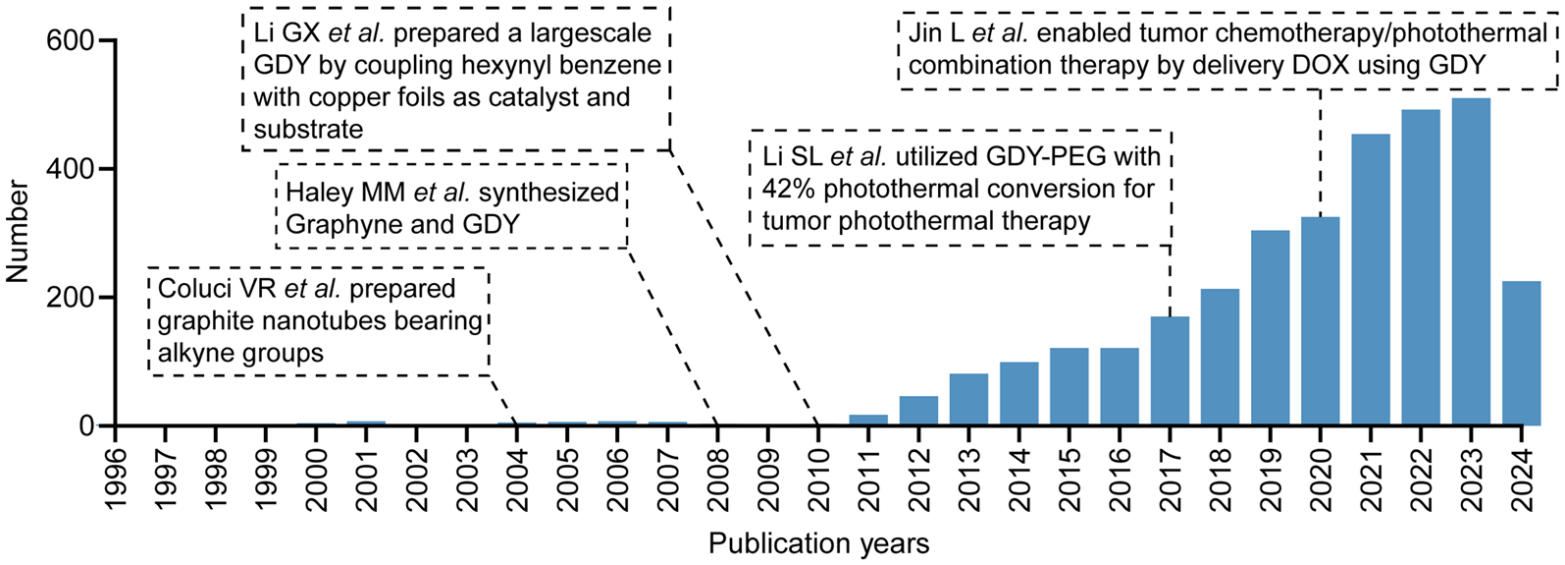
Number of GDY studies reported from 1996 to 2024. Data were obtained from the Web of Science database (www.webofscience.clarivate.com), with a search condition of “Graphyne” or “Graphdiyne”

Theoretical simulations have demonstrated that polycyclic aromatic hydrocarbons can strongly interact with GDY through n-π conjugation, suggesting that GDY can detect polycyclic aromatic compounds with high sensitivity [17]. In 2018, Jin et al. modified GDY using π-π adsorption between the aromatic ring structure of the hydrophobic molecule DOX and the conjugated structure of GDY. This modification resulted in the construction of a nanoformulation with antitumor effects at the cellular level. The findings provided insights for the potential applications of GDY in tumor therapy research [25]. The adsorption of metal ions by GDY differs from that of graphene. In the case of graphene, there is a physical adsorption between the metal atoms and the graphene, whereas the interaction between some of the metal atoms and GDY is a stronger chemical adsorption [81–83]. In a theoretical study, Lin et al. found that Gold (Au), Cu, Iron (Fe), Nickel (Ni), and Pt atoms on GDY nanoribbons exhibited superior thermal stability. The researchers additionally proposed that the Fe, Ni, and Pt atoms adsorbed on GDY are n-type doped, whereas the doping of Cu and Au exhibits metallic properties [84]. The theoretical studies conducted by Ma DW et al. and Lu ZS et al. provided further insight into the adsorption behaviors and electronic structures of Au, Pt, Iridium (Ir), Palladium (Pd), Rhodium (Rh), and Ruthenium (Ru) on GDY. Their findings indicated that noble metal atoms can be strongly adsorbed on the alkyne ring vacancies of GDY [85, 86]. In contrast, Huang et al. employed the high affinity of Fe^2+^ for GDY to synthesize ferric ferrocyanide and iron oxide nanoparticles in situ on the surface of GDY, which demonstrated electrochemical catalytic potential [87]. In order to further enhance the catalytic efficiency of GDY-doped complexes, studies also immobilized Ni/Fe single atoms on the surface of GDY and synthesized GDY/monoatomic metal complexes with exceptional catalytic performance. This innovation in methodology for GDY monoatomic doping study and the enrichment of conditions for the application of GDY represent a significant advancement in the field [88, 89]. Since its preparation, GDY has been subjected to a comprehensive investigation in the fields of photoelectrocatalysis and energy storage. The complex types and methods of modification have endowed GDY materials with a more variable structure and a wider range of properties, which have in turn prompted a gradual attempt to apply GDY materials in biomedicine. In this process, the more comprehensive biomedical studies of GDY materials initiated by Li et al. and Jin et al. in 2017 and 2018, respectively, were seminal and furnished insights and direction for subsequent investigations (Fig. 4) [25, 65].

## Performance test of GDY materials

**Fig. 5:**
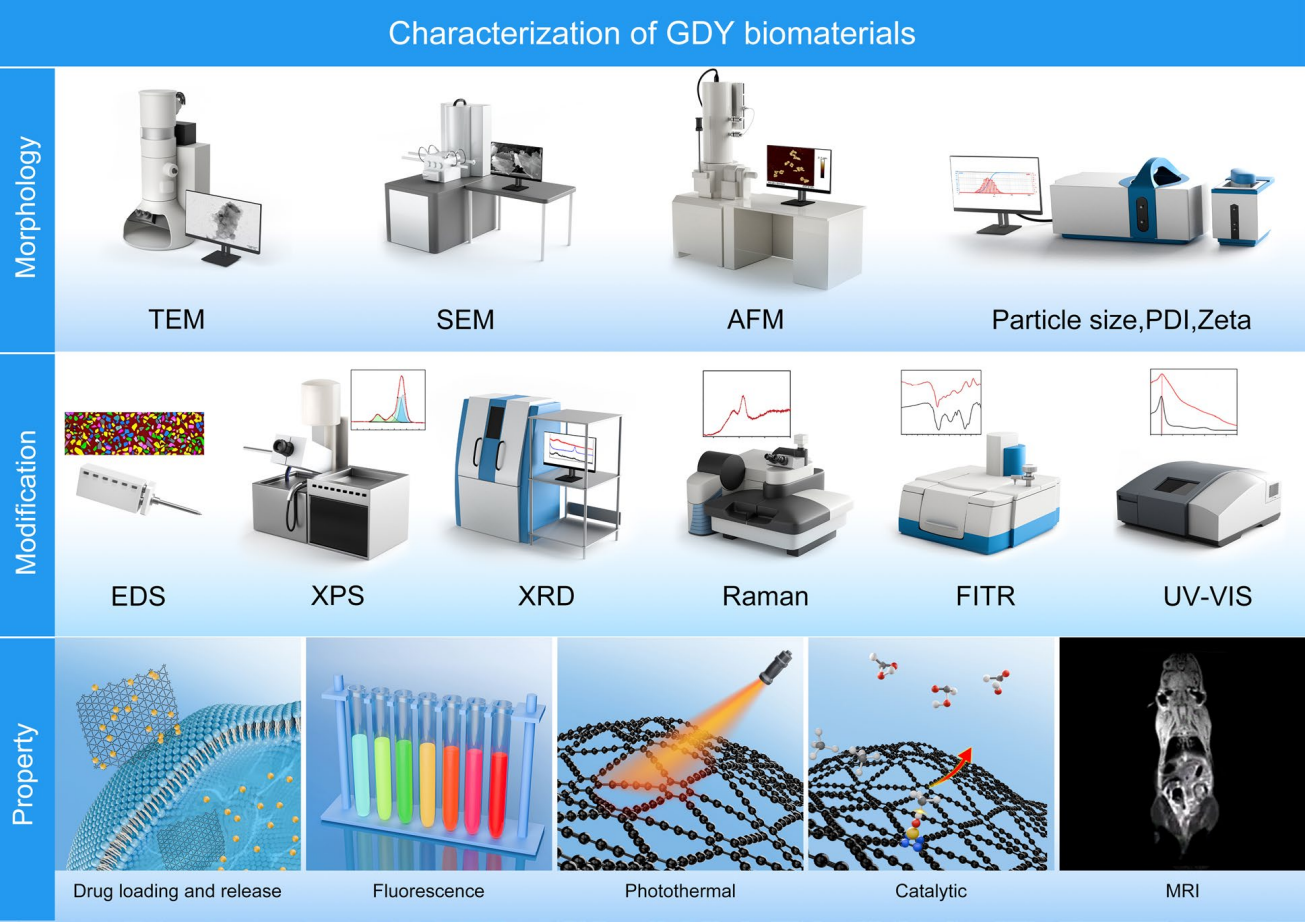
Characterization techniques for GDY biomaterials. Characterization of GDY biomaterials often includes morphological capture, modification analysis, and performance testing

The properties of nanomaterials are of paramount importance in the realization of research objectives, commercial value, and are affected by a multitude of factors, including size, structure, and modifications. Prior to their application, therefore, it is essential to characterize and test the properties of nanomaterials. In biomedical research on GDY nanomaterials, researchers frequently assess their drug-carrying and drug-releasing, catalytic properties, fluorescence properties, magnetic imaging properties, and photothermal properties (Fig. 5) to ascertain that the prepared GDY biomaterials can fulfill the research purpose to a certain extent.

### Drug loading and release

In studying nanodelivery platforms, it is crucial to critically analyze drug loading and release data. This work employs various methods depending on the properties of drugs in nanodelivery systems, with ultraviolet–visible spectrophotometry (UV–Vis) and liquid chromatography-mass spectrometry (LC–MS) being the most commonly used techniques [90, 91]. In the study, “drug” broadly refers to the cargo loaded onto the nanoplatform. This includes, but is not limited to, molecular drugs, nucleic acids, proteins, and metal ions [91–94]. In evaluating the drug-carrying capacity, two metrics, loading content (LC%) and encapsulation efficiency (EE%), were calculated (Eqs. 1, 2).

1 $$\begin{array}{c}LC\%=Weight\, of\, drug\, in\, nanoplatform/Weight\, of\, nanoplatform\times 100\%\end{array}$$

2 $$\begin{array}{c}EE\%=Weight\, of\, drug\, in\, nanoplatform/Weight\, of\, the\; feeding\, drug\times 100\%\end{array}$$

Additionally, LC and EE facilitate the investigation of nanomaterial preparation procedures. The determination of LC and EE depends on the cargo’s characteristics and can be classified into two main categories: direct and indirect techniques. LC and EE can be directly calculated when the cargo in the nanodelivery platform can pass through the column or shows distinctive peaks in UV–Vis spectroscopy. Conversely, LC and EE were calculated indirectly by detecting cargoes that were not loaded into the nanodelivery platform during preparation [90, 95]. In the case of a biomolecule such as a nucleic acid, peptide, or protein, the aforementioned study also applies fluorescence, PCR, or protein quantification to calculate LC and EE [92, 93].

For drug release, it is advisable to employ more precise experimental and computational method. The study generally employed the dialysis method, in which the dispersion of the nanodelivery platform is contained in a dialysis bag and exposed externally to solutions such as water or PBS. A fixed volume of external solution was aspirated at different time points to detect the drug content and the corresponding volume of solution was subsequently backfilled. The drug release profile was obtained by Eq. 3 [96].

3 $$\begin{array}{*{20}c} {r_{n} = {{\left( {{\text{v}}c_{n} + \begin{array}{*{20}c} {n - 1} \\ \sum \\ {i = 1} \\ \end{array} c_{i} } \right)} \mathord{\left/ {\vphantom {{\left( {{\text{v}}c_{n} + \begin{array}{*{20}c} {n - 1} \\ \sum \\ {i = 1} \\ \end{array} c_{i} } \right)} {m_{{initial}} }}} \right. \kern-\nulldelimiterspace} {m_{{initial}} }} \times 100} \\ \end{array}$$

In this equation, r_n_ denotes the release efficiency of the drug, c_n_ denotes the concentration of the drug at the n-th sampling, c_i_ denotes the concentration of the drug at the i-th sampling, m_initial_ denotes the total amount of the drug initially, and n and i denote the number of samplings.

In addition, the dispersion of the material has been investigated by centrifuging at different time points to obtain the drug release profile by detecting the drug content in the supernatant. However, the longer centrifugation time and the adequacy of nanodrug precipitation in this method may affect the accuracy of the results [97, 98]. It is crucial to note that detecting drug release also involves characterizing the responsiveness of nanomaterials to external stimuli, such as temperature, pH, and light. In brief, the drug delivery nanoplatforms are dispersed in solutions with different temperatures, pH levels, or exposed to lasers like NIR. The drug release is then monitored to evaluate the responsiveness of the nanodrug delivery platforms [99–101].

In the GDY biomaterial study, Jin et al. then calculated a LC of up to 38% by directly using UV–Vis detection of DOX in the GDY nanodelivery platform (Figure S1A) [25]. Similarly, Xing et al. obtained up to 40.3% LC of DOX on GDYO nanoplatforms using UV–Vis, and DOX could be continuously released by GDYO nanoplatforms for 24 h. At pH 6.0, 37.2% of DOX was released, whereas at pH 7.4, only 29.7% was released, indicating that the nanoplatforms exhibited pH responsiveness of drug release (Figure S1B) [102]. In addition, Min et al. used inductively coupled plasma mass spectrometry (ICP-MS) to quantitatively analyze the reduced GDYO-loaded Fe, confirming that the Fe loading under experimental conditions reached up to 60%. The Fe release data showed that release was less than 5% at pH 7.4 and 25 °C, and approximately 20% at pH 6.5 and 25 °C. At pH 6.5 and 47 °C, the release of Fe significantly accelerated, indicating that both high temperature and acidic conditions promote Fe release (Figure S1C) [96]. In our previous study, the LC (up to 47.166 ± 3.18%) and EE (68.25 ± 0.72% down to 18 ± 2.28%) were calculated indirectly by detecting unloaded FIN56 in the supernatant. The percentage of FIN56 released from the dispersed at pH 7.4 was 10.24 ± 1.02% in 6 h. In contrast, the total release of FIN56 from the pH 5.0 dispersion was 15.65 ± 1.87%. Furthermore, NIR irradiation significantly enhanced the release of FIN56 in both the pH 5.0 and pH 7.4 dispersion systems, with the total release increased to 60.76 ± 1.53% and 36.62 ± 0.57% respectively (Figure S1D) [12]. Indeed, the importance of LC and EE is that they are fundamental indicators of the application of GDY biomaterials for drug delivery therapy, without which the drug dosage during therapy would be unable to receive regulation.

### Fluorescent property

A significant number of nanomaterials exhibit fluorescent properties, with applications in diverse fields including bioimaging, sensing, optoelectronic devices, and others. To characterize the fluorescence properties of nanomaterials, it is often necessary to analyze them in terms of fluorescence spectra, quantum yields, fluorescence lifetimes, and other related data. This is typically achieved through the use of fluorescence spectrophotometers and time-resolved fluorescence spectrometers [103–105].

Fluorescence spectra encompass excitation spectra, which delineate the fluorescence intensity of a material in response to varying wavelengths of light excitation, and emission spectra, which describe the fluorescence radiation spectra of a material under a fixed excitation light. As illustrated in the emission spectra of violet-phosphorus quantum dots (VPQDs) under diverse wavelength excitations, the optimal excitation wavelength for VPQDs is 430 nm. At an excitation wavelength of 430 nm, a robust fluorescence emission band centered at 522 nm is observed for VPQDs [106]. Additionally, the quantum yield (QY), which measures the ratio of photons absorbed to photons emitted by a material, must also be recorded. Although the formula for calculating QY can vary in research, the most common method is to compare the sample to a standard with a known QY. This is done by measuring the fluorescence intensity. For instance, Kamal et al. calculated the QY of the prepared iron porphyrin bio-mimicked graphene quantum dots using quinine sulfate in 0.1 M H_2_SO_4_ as a reference [107]. We present here a more generalized Eq. 4,

4 $$\begin{array}{c}{\Phi }_{s}={\Phi }_{r}\left({I}_{S}/{I}_{r}\right)\left({\eta }_{S}/{\eta }_{r}\right)\left({A}_{r}/{A}_{s}\right)\end{array}$$

where Φ, I, A and η denote the QY value, fluorescence emission intensity, absorbance and refractive index, respectively, s denotes the sample and r denotes the reference.

Moreover, the fluorescence lifetime, as determined by time-resolved fluorescence spectroscopy, represents a crucial metric for the study of nanomaterials in biofluorescence imaging [105]. In general, the fluorescence decay of nanomaterials can be tested as a function of time by obtaining time-dependent luminescence spectra and fitting the fluorescence decay curves by exponential equations. Herein, we provide a three-exponential equation (Eq. 5).

5 $$\begin{array}{c}{\tau }_{avg}=\left({A}_{1}{\tau }_{1}{\tau }_{1}+{A}_{2}{\tau }_{2}{\tau }_{2}+{A}_{3}{\tau }_{3}{\tau }_{3}\right)/\left({A}_{1}{\tau }_{1}+{A}_{2}{\tau }_{2}+{A}_{3}{\tau }_{3}\right)\end{array}$$

A_1_, A_2_, and A_3_ are the coefficients of the three exponential functions, which represent more different decay processes. These decay processes may include radiative and non-radiative compounding, energy transfer, concentration bursts, etc. τ_avg_ is the average fluorescence lifetime, and τ_1_, τ_2_, and τ_3_ are the time constants of the three exponential functions, respectively. Most of these parameters are generated by detection devices.

Besides, fluorescence polarization (FP), a metric for assessing the difference in intensity of a sample’s fluorescence in different polarization directions, is less commonly utilized in nanoscience [108]. In biomedical applications, Qin et al. utilized MnO_2_ nanosheets conjugated to FAM-labeled DNA to generate FP signals for the detection of organophosphorus pesticides [109]. In contrast, Zhang et al. employed a protein-inorganic hybrid nanoflower based on framework nucleic acid (FNA)-encapsulated proteins for the detection of terminal deoxynucleotidyl transferase (TdT), a potential biomarker for lymphoid tumors. The detection limit was 0.023 U/mL, and linear detection from 0.1 U/mL to 100 U/mL could be achieved in 20 min [110]. It is clear that in the study of the fluorescence properties of nanomaterials, images obtained by fluorescence microscopy represent one of the most direct and convenient techniques for reflecting the fluorescence properties of nanomaterials [13, 111].

The fluorescence properties of GDY biomaterials stem from their quantum dot structure. Min H et al. demonstrated that GDY quantum dots (GDQDs), prepared via the solvothermal method, exhibited an absorption band around 371 nm. The fluorescence emission of GDQDs peaked at 495 nm when excited at 371 nm, as observed through UV–Vis and fluorescence spectroscopy. Additionally, the emission peaks of GDQDs reached their maximum intensity at pH 5 and gradually decreased with any pH deviation from this value. Notably, GDQDs were shown to localize in lysosomes within human umbilical vein endothelial cells (HUVEC) (Figure S2A) [13]. Similarly, Wang L et al. employed confocal microscopy to assess the biosafety of GDYO, and observed fluorescent signals that could be GDQDs in cells of peritoneal fluid (Figure S2B) [111]. In a catalytic study of GDYO quantum dots (GDYO QDs), the authors also discovered that when the excitation wavelength was altered from 371 to 451 nm, the emission wavelength exhibited a red shift, with the strongest excitation peak observed at 411 nm (Figure S2C) [112].

### Photothermal property

The process by which nanomaterials with a strong light-absorbing capacity convert light energy into heat energy after absorbing photons is referred to as photothermal conversion. This phenomenon is primarily attributed to the localized surface plasmon resonance (LSPR) effect, electron–hole generation and relaxation, and conjugation or hyperconjugation effects [113]. In biomedical applications, nanomaterials are employed for the purposes of tumor ablation, photoacoustic imaging (PAI), and NIR imaging, due to their photothermal properties [114, 115].

The characterization of photothermal performance typically encompasses measurements of photothermal conversion efficiency, ultraviolet–visible-NIR absorption spectroscopy (UV–Vis-NIR), thermal stability tests, and thermal diffusivity measurements [114–117]. First, the photothermal conversion efficiency is determined by measuring the temperature change curve of the nanomaterials in the presence of light with an infrared thermographic camera or thermocouple sensor. A more commonly used reference Eq. 6 is provided,

6 $$\begin{array}{c}\eta =\left\{hS\left({T}_{max}-{T}_{sur}\right)-{Q}_{Dis}\right\}/I\left(1-{10}^{-{A}_{\lambda }}\right)\end{array}$$

where h, S, and I denote the heat transfer coefficient, the light-exposed area of the sample, and the laser power, respectively. T_max_ represents the maximum temperature, T_sur_ is the ambient temperature, Q_dis_ the heat loss due to the absorption of light by the vessel, and A_λ_ is the material absorbance.

Additionally, logarithmic and time-dependent curves of temperature change during the cooling phase of photothermal materials are often used to reflect their thermal diffusion behavior, cooling rate, thermal stability, and other properties [118, 119]. Moreover, the absorption spectra obtained through UV–Vis-NIR can be utilized to determine the absorptive capacity of a photothermal material within a specific wavelength range [116, 120], while the application of thermogravimetric analysis and differential scanning calorimetry to assess the thermal stability of nanomaterials is relatively rare in biomedical applications [121, 122].

Due to the photothermal properties of GDY, GDY biomaterials are being frequently applied in tumor PTT. Xing et al. observed that the GDYO solution (concentration of 100 µg/mL, laser condition of 808 nm, 1 W/cm^2^) increased from 22.5 °C to 42.5 °C. The photothermal conversion efficiency of GDYO was calculated to be 47.2%, which was much higher than that of many other PTT agents. Moreover, the maximum temperature of the GDYO biomaterial exhibited only a slight increase following 5 laser on/off cycles, indicating the photothermal stability of the GDYO biomaterial (Figure S3A) [102]. In contrast, Jiang et al. calculated the photothermal conversion efficiency of GDYO to be as high as 60.8% using a 660 nm laser (0.5 W/cm^2^) [123]. In another work, infrared thermography revealed a dose-dependent increase in the temperature of GDY biomaterials under 808 nm (1 W/cm^2^) irradiation. The temperature of the dispersed system at 200 µg/mL reached 58.5 °C (Figure S3B) [96]. In our previous study, we observed a temperature increase of 13 °C at the glioblastoma (GBM) site induced by the target-characterized GDY nanodelivery system under 808 nm (1 W/cm^2^) irradiation conditions using infrared thermography (Figure S3C) [12]. In addition, the photothermal properties of GDY have led to its application in vivo PAI studies. Wang et al. observed through photoacoustic images that the saturated oxygen level at the tumor site increased three-fold after treatment with the GDY material and the NIR laser, suggesting an improvement in tumor hypoxia (Figure S3D) [124]. It is evident that in order to assess the photothermal properties of GDY biomaterials, it is necessary to record temperature changes at different concentrations, under different laser power conditions, and undertake multiple laser on/off cycles.

### Catalytic and magnetic resonance imaging (MRI) properties

The catalytic properties of nanomaterials are often linked to their structural characteristics and modifications. This index is calculated based on the chemical reactions involved in the catalytic process [125]. Metal nanoparticles and metal oxide nanomaterials, such as Pt, Pd, Ag, TiO_2_, and ZnO, are known for their excellent catalytic activity and are commonly employed in redox reactions, photocatalysis, and other catalytic processes [126, 127]. The catalytic properties of GDY biomaterials mainly depend on the properties of the modifiers. For instance, Hemin was immobilized on the surface of GDY/GDYO to create Hemin-GDY and Hemin/GDYO (H/GDYO) catalytic systems with peroxidase properties. The catalytic performance of these systems was evaluated using the reaction of 3,3′,5,5′-tetramethylbenzidine (TMB) in a peroxidase-catalyzed state. In this reaction, H_2_O_2_ reacts with TMB to produce a soluble blue product, measurable at 370 nm or 620–650 nm (Figure S4A, B) [24, 128]. In addition, B-doped and ketone group-rich GDY nano-enzymes, as well as GDY platforms loaded with Fe^2+^ and glucose oxidase (GOx), were employed to assess the catalytic activity of GDY biomaterials via the colorimetric reaction of TMB with H_2_O_2_ [10, 26].

The paramagnetic or magnetic modification of nanomaterials can markedly enhance the contrast and resolution of MRI. Studies use superparamagnetic nanoparticles, gold nanoparticles (AuNPs), and nanomaterials doped with magnetic elements or chemically modified for tumor imaging and brain imaging [129, 130]. The MRI properties of nanomaterials are assessed through MRI scans. Key MRI parameters include T_1_ and T_2_ relaxation times. T_1_ relaxation time relates to the recovery of longitudinal magnetization, while T_2_ relaxation time pertains to the recovery of transverse magnetization. Nanomaterials used as T_1_ contrast agents typically shorten T_1_ relaxation time, increasing signal intensity. Conversely, nanomaterials used as T_2_ contrast agents reduce T_2_ relaxation time, decreasing signal intensity [131, 132]. To date, there have been fewer MRI studies conducted on GDY biomaterials. Min H et al. tested the MRI properties of Fe_3_O_4_-loaded GDYO tumor-targeting iron sponge (TTIS), using an MRI scanner. The results demonstrated that the T_2_-weighted MRI images of TTIS dispersed solutions exhibited a reduction in signal intensity with an increase in Fe concentration/injection time. Additionally, the transverse relaxation value r_2_ of TTIS was determined to be 140 × 10^–3^ m^−1^ s^−1^, indicating that TTIS can be utilized as an effective contrast agent for T_2_-weighted MRI (Figure S4C) [96].

## Characterization techniques for biomedical applications of GDY materials

It is of paramount importance to ensure the stability of the structure, properties, and function of engineered GDY in biomedical applications. Consequently, in the field of biomedical advances involving GDY, researchers frequently, and indeed must, first undertake the characterization of the engineered GDY. In addition to addressing the structural characteristics of GDY materials, these efforts have been more oriented towards analyzing which structures or modifications of GDY materials produce biological functions [24–32]. This is analogous to the characterization of other nanomaterials for biomedical applications, with the distinction that the structural characteristics of GDY materials (*sp*-hybridized carbon atoms, band gap) necessitate more precise characterization work, which may facilitate the stability and reliability of GDY materials during biomedical applications and enhance the possibility of translational applications. This section presents a detailed analysis of the principles, advantages, and limitations of various characterization techniques and the results of representative GDY material characterization (Fig. 5).

### Morphology characterization techniques

In the majority of cases, the morphology of nanoscale materials is acquired by relying on electron microscopy. The acquisition of morphological information not only resolves the size and shape of nanomaterials, but also provides crucial insights into their crystal structure, number of layers, helix angle, and other characteristics. This information is essential for the stabilization of the structure and properties of nanomaterials.

#### Electron microscopy

##### Application of transmission electron microscopy (TEM) and scanning electron microscope (SEM) in nanomaterials research

TEM employs an electron beam to penetrate an ultrathin sample and obtain structural information about the sample by diffraction and imaging of the transmitted electrons. In the case of metallic nanomaterials, in addition to observing the shape, size, and distribution of metal nanoparticles at high resolution, such as the distribution uniformity of metal nanoparticles in catalysts and the effect of particle size on catalytic performance, TEM can also be used to determine the crystal structure and orientation of nanoparticles using selected area electron diffraction. This can help to study the nanoparticles’ growth mechanism and grain boundary properties [133–135]. Furthermore, for materials exhibiting multilayered isomorphous characteristics, such as carbon and P, TEM enables direct observation of the number of layers, crystal defects, and structural features, including the diameter, number of wall layers, and helix angle of nanotubes [136–138]. This information is crucial for comprehending the electronic and mechanical properties of materials. In the case of 1-dimensional (1D) materials, TEM can be employed to ascertain the diameter, length and crystallinity of nanowires, as well as to analyze their growth mechanisms and surface properties [139, 140]. Moreover, in nanoporous materials, TEM is capable of visualizing the pore size, pore distribution, and pore structure of nanoporous materials, which is of significance for applications such as gas storage, catalysis, and separation [141, 142].

As a complementary, the SEM utilizes a focused electron beam to scan the surface of a sample. The secondary electron signals generated by the interaction of the electron beam with the sample are collected by a detector to form an image, which is used to observe and analyze the material’s morphological characteristics, including particle size, shape, and distribution [143]. SEM is a valuable tool for studying the crystalline structure and surface defects of nanomaterials. By employing high-resolution imaging, it is possible to identify microscopic defects such as grain boundaries and dislocations, which are essential for understanding the mechanical properties and stability of these materials [144, 145]. In addition, SEM was employed to examine the surface morphology and pore structure of nanomaterials with the objective of evaluating their catalytic activity [146]. As with TEM, SEM can also be employed to assess the homogeneity and agglomeration of nanomaterials by observing their size, morphology, and distribution.

##### TEM and SEM characterization of GDY biomaterials

As TEM was employed for morphological characterization in the majority of GDY biomaterials studies, we present here the more typical TEM characterization results. In a study of prepared B-doped and ketone-rich GDY nano-enzymes, high-resolution TEM was utilized to observe the bulk structure of GDY and the lattice spacing of 0.41 nm (Figure S5A) [26]. While Wang C et al. observed the prepared nanodiamond/GDY nanoisomers by TEM and demonstrated that these nanoparticles were spherical in structure with a size of approximately 5 nm, and lattice spacings of 0.19 nm and 0.34 nm from nanodiamond and GDY were also observed (Figure S5B) [30]. In addition, Xing E et al. obtained morphology images of the prepared GDY nano-delivery system by TEM and analyzed the particle size distribution of the GDY nano-drug-carrying system by TEM images (Figure S5C) [102]. Additionally, for the study of GDQDs, Min H et al. observed that GDYO QDs had a particle size of about 5 nm and a lattice spacing of 0.42 nm by high-resolution TEM (Figure S5D) [13]. It can be observed that, regardless of the structural composition of GDY biomaterials and the specific applications for which they are employed, the most frequently utilized techniques for characterizing the morphology of GDY biomaterials are those based on TEM.

In contrast, SEM has been used to observe the surface structure of GDY biomaterials. In the study of GDY sensors for real-time analysis of organophosphorus pesticide content, SEM was employed to obtain a morphology image of the GDY sensors, and mesoporous structures were observed on their surfaces (Figure S6A) [27]. Yan et al. prepared N-doped GDY nanosheets by high-temperature carbonization and observed the folded 2D morphology and 3D porous network structure of the material by SEM. This provided sufficient interfaces and capacity for loading of target analytes (Figure S6B) [31]. And in the study of Hou et al. SEM was applied to observe the distribution of AuNPs on the surface of GDY nanosheets (Figure S6C) [147]. Similarly, in another study, SEM was employed to observe the binding and distribution of AuNPs on the GDY surface (Figure S6D) [148]. Consequently, TEM and SEM were utilized as technological complements in the characterization of GDY biomaterials, serving as pivotal methods for observing and analyzing the morphology, size, dispersion, and surface structure of GDY biomaterials.

#### Atomic force microscopy (AFM)

##### Application of AFM in nanomaterials research

The AFM is an analytical device that provides high-resolution imaging of the sample surface, obtaining surface morphology information through the interaction force between the probe and the sample surface [149]. AFM can provide 3D surface morphology information of nanomaterials with sub-nanometer resolution [150]. With regard to the microstructure, particle size, and distribution of 2D materials or layer-structured materials, such as graphene/black phosphorus, AFM enables the observation of the single- and multilayer structure of the material, the measurement of the height difference between the layers, and the study of the surface defects and edge structure of the material [151, 152]. In addition, AFM can be employed to ascertain the mechanical properties of nanomaterials, including hardness, elastic modulus, and viscoelasticity, through the nanoindentation technique. This technique utilizes an AFM probe to apply a min load to the material surface, and then measures the relationship between the indentation depth and the load [153]. Furthermore, when AFM is used in conjunction with magnetic force microscopy, it is possible to observe the magnetic domain structure and domain wall motion of ferromagnetic nanomaterials, and to study the effect of the applied magnetic field on the magnetic properties of the materials, which is crucial for developing magnetic sensors or magnetically targeted nanomaterials [154].

##### AFM characterization of GDY biomaterials

The AFM technique is generally used to determine the thickness of GDY biomaterials as a reflection of the morphology of GDY materials. Chang et al. characterized the thickness of the prepared GDY nanosheets to be 0.9 nm by AFM (Figure S7A) [28]. Zheng et al. used AFM to characterize the prepared GDY film-like sensor platform, and the data showed that the thickness of the GDY film was about 3–4 nm (Figure S7B) [155]. While GDY electrodes modified with AuNPs were prepared in another study, AFM analysis revealed a thickness of 3.6 nm for GDY nanosheets (Figure S7C) [147]. Xu et al. in contrast, used AFM to examine the GDY sandwich structure prepared for detecting tumor markers, and the AFM results showed a GDY thickness of 10.4 nm for the sandwich structure (Figure S7D) [148]. Thus, it is clear that AFM is most often used to study flaky GDY, while AFM is rarely used to study GDY in other shapes.

### Size, dispersion and zeta potential

Compared to morphology detection techniques, there is a single method for detecting the size and dispersion of nanomaterials. In addition to the statistical analysis of size by TEM, SEM and AFM images, the most commonly used device is the laser particle size analyzer, a device that uses a laser light source to measure the size and distribution of powder particles by the principle of light scattering, which has the advantages of a wide range of measurements, rapid analysis of results and good reproducibility [156]. When the laser light emitted from the instrument into the sample passes through an inhomogeneous medium, it undergoes absorption, reflection, refraction, transmission and diffraction, which cause the light to deviate from its original path. By measuring the intensity and angle of this scattered light, the size and distribution of particles is calculated based on dynamic light scattering (DLS) [157].

The characterization of nanomaterials by laser particle size analyzer typically yields three metrics: particle size, polydispersity index (PDI), and zeta potential. Among the aforementioned metrics, the particle size, which is typically determined by a laser particle sizer in a liquid medium, is generally understood to refer to the hydrodynamic radius. This is the effective particle size of the nanoparticles, which are formed together with the water molecule layer (solvent molecule layer) carried by the nanoparticles when they are moving in solution. This value is generally slightly larger than the actual particle size of the nanomaterials obtained by TEM, SEM, or AFM [158]. In addition, PDI is a dimensionless value that measures the uniformity of particle size distribution in a particle sample and is often used to characterize the particle size distribution of nanomaterials. The value of PDI ranges from 0 to 1. A smaller PDI value indicates greater uniformity in particle size and a more concentrated distribution of particle sizes. In theory, a PDI value of 0 signifies that all particles have an identical size. However, in practice, this is a challenging scenario to achieve [159]. Furthermore, zeta potential is a physical quantity that describes the surface charge state of nanoparticles in solution. It reflects the charge distribution between the particle surface and the surrounding solution. It is therefore an important parameter in the measurement of surface charge and the stability of the particles. The absolute value of the zeta potential represents the strength of the surface charge. The larger the value, the stronger the surface charge. A positive value indicates that the surface is positively charged, which implies that negatively charged ions are adsorbed on the surface of the particles, and vice versa [160]. In nanomaterials research, zeta potential is correlated with the interaction of nanoparticles in solution and can be employed to anticipate the stability of particles. In general, high absolute values of zeta potential are typically associated with good dispersion and stability [161, 162].

Thus far, it has been observed that the particle size, PDI, and zeta potential are closely related. The data from these three indexes can not only reveal the characteristics of the nanomaterials, but moreover, can reflect the authenticity of the data. For instance, when the absolute value of the zeta potential is approximately 0, the PDI value and particle size are frequently considerable, and the nanomaterials present in the analyzed samples are prone to agglomeration [163, 164]. If this is not the relationship between the three metrics, it is important to investigate further the characterization of the nanomaterials or to question the authenticity of the results. Furthermore, the reliability of the study is more effectively demonstrated when particle size, PDI, and zeta potential are analyzed in conjunction with TEM, SEM, and AFM data.

The data pertaining to particle size, PDI, and zeta potential appear to be of particular importance in the context of studies on GDY biomaterials, particularly those pertaining to the delivery of nanoparticles. In a study applying GDY-coated Fe_3_O_4_@UIO-66-NH_2_ (FUGY), the hydrodynamic radius of FUGY was determined to be approximately 272.5 nm ± 63.5 nm with a PDI of 0.362, which is greater than that of Fe_3_O_4_@UIO-66-NH_2_ (148.3 nm ± 32.1 nm, PDI of 0.171). This indicates that GDY capping increased the size of the nanoplatform and negatively modulated its dispersion (Figure S8A) [165]. Xing et al. prepared GDYO-cisplatin (CDDP)/DOX@1,2-Distearoyl-sn-glycero-3-phosphorylethanolamine-PEG-methotrexate (MTX) (GCDM) loaded with DOX and MTX on CDDP-modified GDYO nanosheets (GDYO-CDDP). The combination of the TEM images revealed that the GDYO-CDDP was a lamellar structure with an average hydrodynamic radius of 342 nm, while the GCDM was a regularly shaped spherical particle with an average hydrodynamic radius of 250 nm. Furthermore, the surface potential exhibited an increase from −34.5 mV (GDYO-CDDP) to −8.7 mV (GCDM) following the loading of positively charged DOX (Figure S8B) [102]. It is of significant importance to note that Wang et al. conducted a comparative analysis between GDYO and GO, with a particular focus on the size and potential changes observed in both materials under varying pH conditions and dispersion times. The findings indicated that the zeta potentials of both GDYO and GO were susceptible to pH alterations. However, at a specific pH, the zeta potential of GDYO was observed to be less pronounced than that of GO. Moreover, in both saline and cell culture medium, the hydrodynamic radius of GO exhibited an increase over the course of 120 min, while the hydrodynamic radius of GDYO remained constant. This study also demonstrated the superiority of GDYO over GO for biological applications (Figure S8C) [166].

### Characterization techniques for modifications

Modifications of nanomaterials confer additional properties and functions, and thus, the detection and characterization of the modifying elements are important data for ensuring the success of the engineered nanomaterial preparation process. These modifying elements may be either inorganic or organic. Inorganic elements may include metals, transition metals, nonmetals, or organic elements such as those containing C=O, C–H, or C–*R* structures, or polymers. With the development of specialized equipment and techniques, researchers have more ways to accurately characterize and identify the modifications of nanomaterials.

#### Energy dispersive spectroscopy (EDS)

##### Application of EDS in nanomaterials research

EDS is a technique based on X-ray spectroscopy that employs an electron beam to bombard the surface of a sample, thereby enabling the collection of characteristic X-rays emitted by excited atoms in the sample. This approach is utilized for the analysis of the elemental composition of a substance, often in conjunction with SEM or TEM. EDS can provide information on the elemental composition and elemental distribution of specific regions on and within the surface of nanomaterials through point analysis, line scanning, and surface scanning. The technique is employed to ascertain the presence of the targeted elements in the nanomaterials and to determine the elemental composition and distribution within the nanomaterials. This is done to assess the efficiency of the nanomaterial preparation process [167, 168]. Furthermore, studies employing EDS have been conducted to examine the active components on the catalyst surface and the composition of the post-reaction products, with the objective of optimizing the design and performance of the catalysts [169], or analyzing the potential causes of the degraded performance of the nanomaterials, including impurity contamination and elemental migration [170, 171].

##### EDS characterization of GDY biomaterials

The GDY/hemoglobin chloride complexes prepared by Hao et al. for real-time monitoring of nitric oxide levels were analyzed for the distribution of the elements N, O, and Fe on the material by applying the EDS technique of TEM (Figure S9A) [29]. In the study of Liu et al. the distribution of O and Fe elements on GDY nanosheets was also analyzed using EDS (Figure S9B) [10]. In the study of Au nanoparticles/GDY-modified electrodes, EDS analysis was employed to ascertain the distribution of Au and C on the electrode (Figure S9C) [172]. In B-doped GDY nanoenzymes, the authors employed EDS to investigate the binding and distribution of B on GDY (Figure S9D) [26]. It is evident that TEM combined with EDS analysis has been employed in GDY biomaterials research to assess the distribution of modifiers in GDY materials. Based on the EDS results, X-ray photoelectron spectroscopy and Raman spectroscopy have been conducted to provide supplementary evidence to ascertain the efficiency of modifications in GDY biomaterials.

#### X-ray photoelectron spectroscopy (XPS)

##### Application of XPS in nanomaterials research

In contrast to the X-rays emitted from an EDS-captured sample, XPS employs X-rays to irradiate the surface of the sample, thereby exciting the atoms on the surface of the sample to produce photoelectrons. These photoelectrons carry information related to the binding energies of the atoms in which they would otherwise be located. This information reflects the elemental species, chemical state, bonding, and valence of the elements in the sample [173, 174]. Consequently, XPS is most frequently employed in the analysis of nanomaterials comprising elements such as Fe, Cu, O, and S, with the objective of determining the valence states of these elements in response to material characterization or activity [175, 176]. In addition, it has been reported to analyze the film thickness and layer structure of nanomaterials by combined XPS and ion sputtering techniques [177, 178], or alternatively, by progressively removing the surface material of the specimen in layers and recording the photoelectron spectrum of each, the depth distribution of the composition can be ascertained [179].

##### XPS characterization of GDY biomaterials

XPS characterization is generally employed to identify the valence and chemical bonding states of elements on GDY biomaterials. The work of Wang et al. utilized XPS to analyze GDY nanosystem loaded with Hemin and demonstrated that Hemin was immobilized on GDY by the distribution of peaks in the spectra of N(*1 s*), Fe(*2p*), and C(*1 s*).The oxidation states of Fe were identified as Fe^3+^ and Fe^2+^, while the carbon atoms were found to be in the following states: C=O, C–O, C≡C(*sp*), and C=C(*sp*^*2*^) (Figure S10A) [24]. Similarly, in another nanosensor study constructed from Hemin and GDYO, XPS was applied to analyze whether Hemin was successfully loaded onto GDYO. The authors concluded that the oxygen-carrying capacity of GDYO was much higher than that of GDY based on the XPS mapping (Figure S10B) [128]. Moreover, the study revealed that the binding energies of Zn(*2p*_*3/2*_) and Zn(*2p*_*1/2*_) in the XPS spectra of ZnO nanorods@GDY nanosheets (ZnO@GDY NR) were 0.15 eV lower compared to ZnO. This indicated a strong interaction between ZnO and GDY, as evidenced by the XPS data (Figure S10C) [180]. Surprisingly, Guo et al. analyzed GDY and GDYO QDs by XPS and found that the carbon/oxygen (C/O) ratio decreased from 5.48 in GDY to 3.03 in GDYO QDs, indicating the increase of oxygen-containing groups in GDYO QDs. Also, the high-resolution C(*1 s*) spectra of GDYO QDs and GDY demonstrated that GDYO QDs still have the backbone structure of GDY (Figure S10D) [112]. These endeavors have positioned XPS as a pivotal player in the characterization of GDY biomaterials. Its contributions include the analysis of changes in elemental valence, chemical bonding, and binding energies.

#### X-ray diffraction (XRD)

##### Application of XRD in nanomaterials research

XRD is a technique employed to analyze the structure of crystalline materials. It is based on the fundamental principle of Bragg’s law, which states that when X-rays are exposed to a crystalline material, the arrangement of the atoms in the crystal causes diffraction of the incident X-rays [181]. XRD is a technique most often utilized in nanoscience to ascertain the crystalline phase composition of nanomaterials. This process provides data on their crystal structure, including lattice parameters and symmetry [182, 183]. In brief, the most prevalent application of XRD technology is to ascertain whether the nanomaterials in question are crystalline, and to determine the crystal structure and associated characteristics. For those nanocrystalline structures, XRD is additionally able to determine the size and morphology, as well as to conduct investigations into phase transitions, thermal stability, stress analysis, and defect analysis [184–187]. Here, the concept of crystal is a source of confusion for non-specialized researchers. In a broad sense, the term crystal structure encompasses not only 3D nanomaterials, such as nanorods and nanowires, but also 1D and 2D materials, such as graphene, which also possess a crystal structure [183, 188, 189]. For instance, the XRD results of graphene and GO demonstrate that graphite exhibits a characteristic peak at 26.6°, while the peak of GO appears around 10.7°. The displacement of the diffraction peak of graphite from 26.6° to 10.7° implies that the distance between the graphene layers increases, which should result in the formation of spaces between the layers due to oxidation [189]. By analyzing the XRD data, it can also be clarified to some extent whether the nanomaterials have modifications present or not.

##### XRD characterization of GDY biomaterials

XRD has been employed to a lesser extent in the field of GDY biomaterials research. However, XRD has played a pivotal role in the advancement of GDY materials, including the analysis of their crystalline structure, number of layers, size, and modifications [190, 191]. Bai et al. employed XRD to characterize the crystalline structure of the prepared CuS@GDY cubes. Their findings indicated that the XRD patterns of CuS@GDY were consistent with hexagonal CuS, exhibiting diffraction peaks at 28.0°, 29.3°, 31.7°, and 47.9° corresponding to (101), (102), (103), and (110) crystalline facets, respectively. Notably, the original GDY (002) crystal face did not appear in CuS@GDY (Figure S11A). The authors postulated that this phenomenon may be attributed to the lower degree of crystallinity exhibited by GDY on the CuS surface [191]. Furthermore, XRD was also utilized to analyze the structures of nanodiamonds (NDs) and hydrogen-substituted GDY (HsGDY) isomers (HsGDY@NDs). The broad peak at 26.5° in the XRD pattern of HsGDY corresponds to an interlayer distance of 0.34 nm, and two peaks, 26.5° and 43.8° (the diamond planar characteristic peaks) were obtained simultaneously in the diffraction pattern of HsGDY@ND hybrids, indicating that the HsGDY and NDs were fully integrated (Figure S11B) [30]. Moreover, research examined the state of Fe^2+^ in Fe-GDY nanosheets. The conclusion that Fe^2+^ was adsorbed on the surface of GDY rather than forming a crystal structure was reached based on the observation that the XRD pattern of Fe-GDY did not exhibit specific peaks from the Fe lattice surface (Figure S11C) [10]. Consequently, via an analysis of the reports pertaining to GDY biomaterials, it was found that XRD is a more frequently utilized technique for the analysis of the integration of GDY modifiers in a combined application with XPS and Raman.

#### Raman

##### Application of Raman in nanomaterials research

Raman spectroscopy is an analytical method based on the interaction of light with matter. It is widely used in nanomaterials research, where it is employed to measure Raman scattering spectra of molecular vibrations, rotations, and other low-frequency modes of a sample. This enables the determination of the molecular structure and properties of matter [192]. Indeed, Raman spectroscopy is most frequently employed in nanoscience to provide information on the molecular fingerprints of specific materials, which are then used to identify and characterize the chemical composition and structure of nanomaterials [193]. For instance, in the structural characterization of GDY materials, the graphitization degree, defects, and impurities of the materials are determined by the analysis of characteristic peaks, such as the D and G bands [194]. In addition, Raman spectroscopy is capable of analyzing the crystal structure and phase transition information of nanomaterials. This enables the identification of the crystalline phases of the materials through the Raman peak shifts and intensity changes of different phases. Furthermore, Raman spectroscopy has been utilized by researchers to identify and monitor the states and phase transition processes of the two phases, anatase and rutile, in TiO_2_ nanomaterials [195]. Moreover, Raman spectroscopy is highly sensitive to stress and strain, and the displacement of Raman peaks can be employed to detect the state of stress and strain in nanomaterials, as well as to analyze the mechanical properties of materials during preparation and application. This can be achieved by measuring the stress distribution in semiconductor nanowires and nanofilms [196, 197]. Furthermore, Raman spectroscopy enables the real-time monitoring of intermediate and final products during chemical reactions, providing invaluable insights into reaction kinetics and mechanisms. This is particularly advantageous in photocatalytic and electrocatalytic reactions, where it allows for the real-time monitoring of reactants and products in catalytic reactions [198, 199]. It is crucial to highlight that Raman spectroscopy is exceptionally adept at probing into the surfaces and interfaces of nanomaterials. This enables the determination of information such as surface adsorption, chemical modifications, and other related characteristics. This data is instrumental in the characterization of the molecular structure of surface-modified nanomaterials and the interactions at the interfaces of different phases within nanocomposites, offering invaluable insights into their structural and compositional properties [200, 201]. In recent years, Raman spectroscopy has been employed to investigate the optical properties of quantum dots and nanocrystals, including their photonic and photonic interactions [202–204].

##### Raman characterization of GDY biomaterials

In GDY biomaterial applications, most of Raman data has been employed to ascertain the graphite content or GDY structure within the material. In the case of N-doped GDY material, the Raman pattern exhibited two peaks at 1369 cm^−1^ (corresponding to *sp*^*3*^ carbon respiratory vibration D band) and 1571 cm^−1^ (associated with *sp*^*2*^ in-phase tensile vibration order scattering E2g mode G band). Moreover, the impact of N-elemental doping on the graphite content within the material was evaluated based on the intensity ratio of the D/G band (Figure S12A) [31]. Similarly, Niu et al. observed two characteristic peaks at 1388 cm^−1^ and 1573 cm^−1^ in the prepared material by Raman spectroscopy, corresponding to the D band and G band, respectively. The inferred higher structural regularity of the GDY network in the material was indicated by a D/G ratio of 0.6 (Figure S12B) [27]. In addition, Wang et al. employed Raman spectroscopy to characterize three prepared samples, which exhibited two distinct peaks at 1370 cm^−1^and 1597 cm^−1^. These peaks correspond to the D and G bands of the carbon vibrational domain in the GDY structure, respectively (Figure S12C) [30]. In general, Raman data of Hemin-modified GDY materials exhibited a broader D band and G band than that of the primary GDY. This suggested that an electron transfer occurs between GDY and Hemin, and that Hemin is loaded on the GDY surface (Figure S12D) [24]. The aforementioned studies demonstrate the utility of Raman spectroscopy in characterizing graphite content, GDY structural integrity, and modifier binding in GDY biomaterials.

#### Fourier transform infrared spectroscopy (FTIR)

##### Application of FTIR in nanomaterials research

FTIR is a spectroscopic technique for analyzing the molecular composition and structure of a sample by measuring the interaction of infrared light with substances. FTIR spectrometers are widely used in materials science research by generating interferograms through an interferometer, which are then converted to infrared spectra through a fourier transform [205, 206]. Based on this property, FTIR is used to detect functional groups such as carboxyl, hydroxyl, and carbonyl after surface functionalization in carbon-based nanomaterials such as carbon nanotubes, graphene, and other carbon-based nanomaterials [207, 208], as well as in surface modification studies of metal nanoparticles to verify the successful bonding of organic molecules such as thiols and amine groups to the surface of nanoparticles [209, 210]. It is noteworthy that FTIR is capable of analyzing chemical interactions at the interface of nanomaterials with other materials. This includes the detection of bonding at the interface of polymers and carbon nanotubes, which is of particular importance for the characterization of biomaterials [211]. Furthermore, the molecular alterations on the surface of nanomaterials, as observed through FTIR analysis, have been utilized in the investigation of the crystalline phase structure and phase transitions of nanomaterials [212, 213].

##### FTIR characterization of GDY biomaterials

In the field of GDY biomaterial applications, FTIR was employed to analyze the modification of GDY materials. Bahari et al. utilized FTIR to identify peaks at 3422.29 cm^−1^ and 3454.36 cm^−1^, which correspond to NH_2_ groups. This demonstrated that the Metal–organic framework (MOF) were successfully modified onto GDY nanodots (Figure S13A) [32]. Chauhan et al. employed FTIR to ascertain the characteristic peaks of Triethynylbenzene and the prepared HsGDY material. Their findings revealed that the distinctive ≡C–H peak at 3271 cm^−1^ was present exclusively in triethynylbenzene and was not detected in the HsGDY material. This observation indicated the successful preparation of the material (Figure S13B) [214]. In an antitumor application of GDY biomaterials, FTIR analysis results showed benzene ring stretching vibrations at 1449 cm^−1^ and 1603 cm^−1^, and C≡C stretching vibrations at 2122 cm^−1^ and 2207 cm^−1^ of GDY (Figure S13C) [165]. In contrast, another study performed FITR analysis on the most commonly applied GDY and GDYO, which showed the presence of the characteristic C=O absorption peak at 1720 cm^−1^ for GDYO, suggesting that the GDYO material had undergone oxidation in comparison to the GDY material (Figure S13D) [102]. In summary, the FTIR technique was used in the characterization of GDY biomaterials to study whether the final product was successfully synthesized by analyzing the characteristic peaks of chemical bonding.

#### UV–Vis

##### Application of UV–Vis in nanomaterials research

UV–Vis is a technique employed for the analysis of the composition and properties of samples, whereby their absorption spectra are measured in the ultraviolet and visible regions. This process provides information regarding the molecular structure, electronic state, and concentration of the samples [215–217]. The use of UV–Vis is pervasive in nanoscience, where it is employed to infer structural or compositional alterations in materials by comparing spectral data between different samples. One of the most prevalent applications is the study of interfacial and surface modifications, where the presence and distribution of modified molecules are determined by analyzing changes in the characteristic absorption peaks [218, 219]. In addition, the optical properties of nanomaterials, such as absorption peak positions and shapes, are closely related to their sizes and morphologies. Consequently, some studies have employed UV–Vis analysis to infer the sizes and morphologies of nanoparticles by examining their plasma resonance peaks [220, 221], thereby assessing the stability and aggregation of nanomaterials [222, 223]. It has also been proposed that the absorption peaks observed in the NIR indicate that the nanomaterials may possess NIR-triggered photothermal conversion property [116].

##### UV–Vis characterization of GDY biomaterials

The modification of GDY biomaterials is frequently analyzed through the use of UV–Vis studies, which are then used as evidence to speculate on the binding mechanisms of the modifier to GDY. In the GDY material loaded with Hemin, the study employed UV–Vis spectroscopy to ascertain that the Q bands of Hemin in the Hemin-GDY material exhibited a 4 nm shift relative to free Hemin. This observation led to the hypothesis that Hemin forms a π–π linkage with GDY through its Fe^2+^ (Figure S14A) [24]. Similarly, in another study, UV–Vis data demonstrated that the characteristic peaks of Hemin exhibited red-shifted values of 400 nm and 655 nm when loaded with Hemin on GDYO, indicating an increase in the conjugation system and a decrease in the transition energy. This further corroborated the π–π stacking interaction between Hemin and GDYO (Figure S14B) [128]. In contrast, Chauhan et al. observed a broad band centered at 548 nm by UV–Vis spectra of HsGDY and calculated a band gap of 1.98 eV after Kubelka–Munk conversion, indicating that HsGDY exhibits semiconducting properties (Figure S14C) [214]. Moreover, the study employed UV–Vis spectroscopy to compare GDYO, Fe_3_O_4_ nanoparticles, and GDYO-Fe_3_O_4_ nanoplatforms. The results indicated that the absorbance of GDYO-Fe_3_O_4_ at NIR was significantly enhanced, which was attributed to the reduction of GDYO during the solvothermal synthesis of GDYO-Fe_3_O_4_ (Figure S14D) [96]. Furthermore, UV–Vis spectroscopy is frequently employed in the investigation of catalytic processes associated with GDY biomaterials. This analytical technique is utilized to assess the catalytic efficiency of the system by monitoring the change in absorbance following the addition of the substrate [10, 180].

## Research strategy for GDY biomedical products

**Fig. 6:**
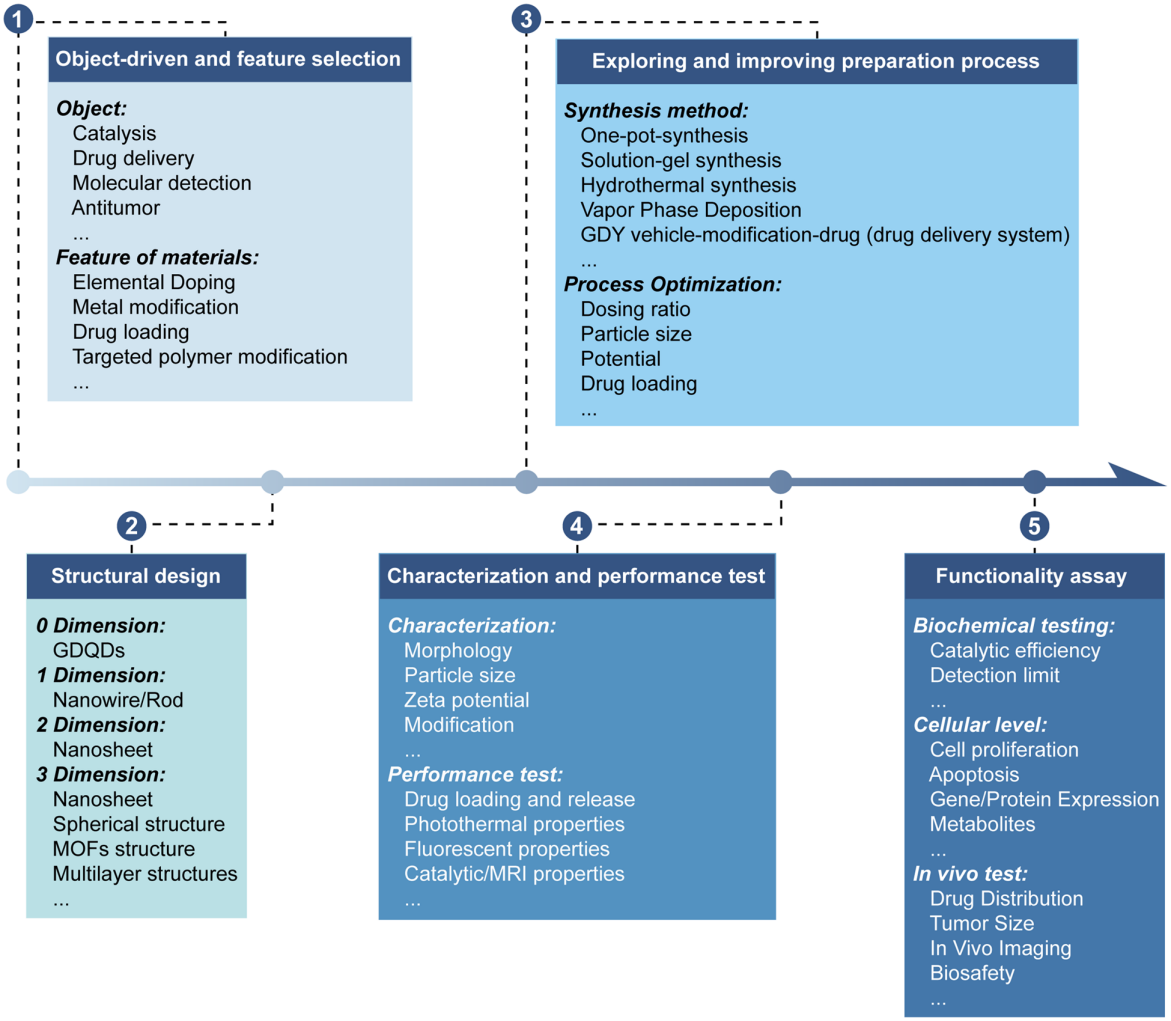
Research strategy for GDY biomedical products. We suggest a more general research strategy, which starts with object-driven and feature selection, and proceeds through structural design, exploring and improving preparation process, characterization and performance test, and functionality assay to comprehensively assess the applicability of GDY biomaterials

The preparation of GDY biomaterials follows the following strategies and processes: setting research objectives, determining material functionality, structural design, process scheming, preparation procedure exploration and refinement, material characterization and performance testing, in vitro/cellular functionality testing, and in vivo/animal experiments. Many metrics, parameters, and data need to be acquired in each step of the process (Fig. 6).

### Object-driven and feature selection

Firstly, it is evident that the majority of applied research is goal-oriented, and this is also true of biomedical applications of GDY. The initial step in any research project is to define the research objectives, which are then followed by the determination of the functions that the GDY biomaterials must possess. Currently, the biomedical applications of GDY materials are focused on achieving their catalytic, drug delivery, detection, and antitumor effects [25, 26, 224, 225]. A variety of modifications and transformations of GDY biomaterials are employed for diverse research purposes. For instance, the loading or modification of metal ions, proteases, and other components on the surface of GDY is investigated to ascertain its suitability for biocatalytic applications [10, 24]. And for drug delivery and anti-tumor purpose, GDY was utilized to load chemotherapeutic drugs and targeted polymers to achieve tumor tissue targeting and therapeutic efficacy [12, 102]. Accordingly, the initial and closely related work steps are the definition of the research objectives of GDY biomaterials and the determination of their intended functions.

### Structural design

The fundamental process in the preparation of GDY biomaterials is the design of their structures, with different structures often exhibiting distinct functional properties. Although the research and variety of GDY biomaterials are gradually increasing, the structural design of GDY biomaterials can be broadly categorized into three types: GDQDs, 1D nanowires/rods made of GDY, 2D nanosheets made of GDY, and 3D nanostructures made of GDY. GDQDs have been used in studies of enzyme catalysis, molecular detection, and bio-imaging [13, 112, 226], while 2D-structured GDY nanosheets have been widely employed for drug delivery, catalysis, molecular detection and anti-tumor applications due to their large surface area [12, 24, 123]. In contrast, GDY 3D nanomaterials generally utilize GDY as a raw material to form 3D structures in conjunction with other nanomaterials, such as MOF and metal nanoparticles [30, 165]. The structural determination of GDY biomaterials serves as the foundation for the realization of the biological functions and research objectives of GDY.

### Exploring and improving preparation process

Subsequently, the central step in demonstrating the innovativeness of the research is to describe the method used to prepare the desired GDY material. In this context, various research groups have developed distinct preparation methodologies. One example of a common approach in catalysis research is the one-pot synthesis method, which involves the direct conversion of the raw materials into the final product within a single reaction system [180, 227]. In contrast, in drug delivery studies, it is typically necessary to modify the targeting polymer on the GDY, load the drug, and finally obtain the targeted drug delivery system [12, 228]. In essence, this aspect of the work necessitates that researchers delineate the requisite scheme for the fabrication of GDY biomaterials. Additionally, the solution-gel synthesis method and vapor phase deposition method are employed in the current GDY biomaterial preparation process [96, 229].

Then, the preparation process of GDY biomaterials must also be adjusted for optimization. Similarly, liposome products with highly developed processes also necessitate adjustment of the feeding ratio to obtain liposomes with suitable conditions, such as particle size, potential, or drug loading, in laboratory studies [230]. The optimization of GDY biomaterial preparation processes is often reflected in the study of nano-delivery systems. In these systems, the size and drug loading of GDY nano-delivery systems are controlled by controlling conditions such as sonication time, type of modification, and drug/GDY nanosheet ratio [12, 25].

Regrettably, with the exception of nano-delivery studies, the other studies often showed only the optimal preparation parameters and conditions for GDY biomaterials and did not adequately elaborate the effects of different parameters or conditions on the preparation of GDY biomaterials [128, 231]. Indeed, this section is of paramount importance for future researchers engaged in GDY studies. While the key indices of the prepared materials are essential, the optimal preparation process, which is contingent upon continuous exploration and adjustment, is a process that warrants mention in the article.

### Characterization and performance test

The characterization and performance testing of the prepared GDY materials are conducted to ascertain the function that they are intended to achieve. In this context, data pertaining to the morphology, particle size, and zeta potential of the materials are typically required [102, 165]. In addition, in drug delivery studies, drug loading/release assays are required [12, 25]. For photothermal studies, temperature curves need to be plotted under light [102, 123]. It is of paramount importance to note that if the prepared GDY materials have modifiers, characterization of these modifications is also essential. Material characterization and performance testing provide data support to ensure the functionality of the prepared GDY materials and represent one of the core focuses of the research.

### Functionality assay

Functionality experiments can be divided into two categories: in vitro and in vivo. The in vitro level encompasses cellular and biochemical experiments, while the in vivo level studies are conducted in living organisms. This final stage of the work is crucial for verifying the functionality of the prepared GDY materials. Firstly, the catalytic, detection, and anti-tumor effects of GDY should be determined by cellular or biochemical experiments. Several indexes, including catalytic efficiency, detection limit, cell proliferation, apoptosis, gene/protein expression, and metabolites, should be tested [11, 112, 232]. Subsequently, the validation of effects such as drug delivery, imaging, and antitumor at the in vivo level is required. In this context, data on drug distribution, drug metabolism, imaging effects, tumor size changes, and related gene/protein expression, as well as biochemical indicators, must be collected [12, 102, 233]. In addition, biosafety experiments are also important assessment indicators for biomedical applications of GDY materials. These are usually obtained through hemolysis experiments, pathological staining of organs, and liver and kidney function tests [25, 96, 165]. In general, functional and biosafety experiments represent the final stages of GDY biomedical application research. By integrating the aforementioned characterization results and performance data, the research objectives initially proposed at the outset of the project can be ultimately achieved, thereby facilitating the advancement and advancement of GDY materials for biomedical applications.

## Biomedical applications of GDY

**Fig. 7:**
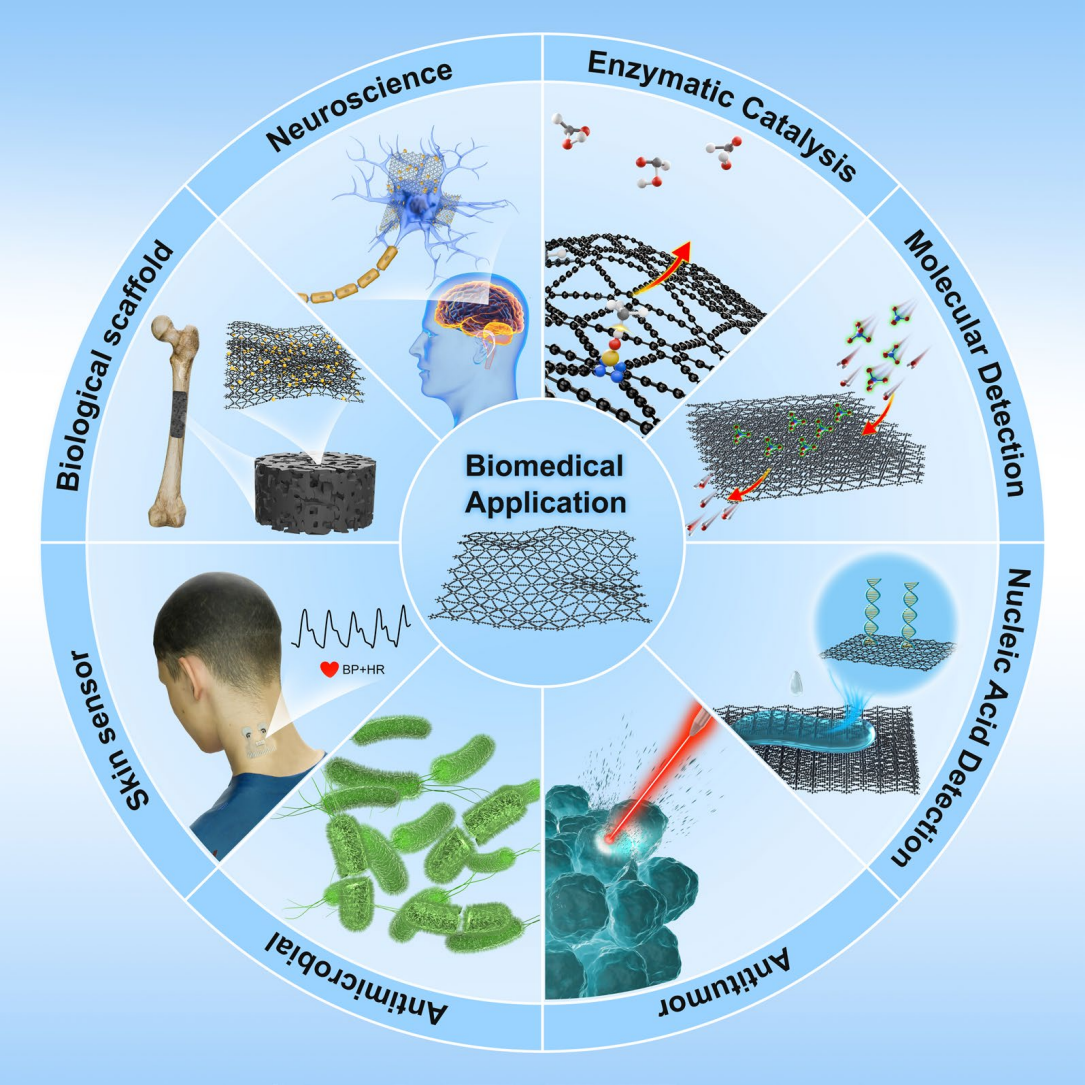
Biomedical applications of GDY. GDY biomaterials are used in enzyme catalysis, molecular detection, nucleic acid detection, antitumor, antimicrobial and sensor applications

With the advancement of research into GDY in photoelectrocatalysis and energy storage, investigators have initiated preliminary investigations into the potential of GDY for biomedical applications. The fully conjugated structure and the substantial hydrophobicity of GDY suggest the possibility of employing GDY for doping of ions and drug-carrying agents. The robust affinity for transition metal atoms and the reactive alkyne bonding structure enables GDY to be utilized as a highly sensitive detection system in a variety of fields. Moreover, GDY’s robust light absorption at NIR and exemplary photothermal conversion efficiency render it a promising candidate for the field of PTT for tumors [20–22, 25]. After a comprehensive examination of the characterization techniques and research strategies associated with GDY materials, a summary of the biomedical applications of GDY materials is presented, providing researchers with a reference point for future endeavors (Fig. 7 and Table 1).

**Table 1:** Biomedical applications of GDY

Type	Name	Modification	Application	References
Enzymatic catalysis	Hemin-GDY; H/GDYO	Hemin	Degradation of organic contaminants, blood glucose test	[24, 128]
	B-GDY	B	Glucose test	[26]
	ZnO@GDY NR	ZnO	skin wound sterilization	[180]
	GDYO QDs	N/A	N/A	[112]
	Ru@GDYO	Ru	N/A	[227]
	Fe-GDY/GOx	Fe^2+^ and GOx	One-step blood glucose test	[10]
Molecular detection	GDY-IL	Au electrode	Rapid detection of rutin	[234]
	GDY/HEM	Hematin	Nitric oxide detection	[29]
	NUGDY	N	6,7-dihydroxycoumarin detection	[31]
	GDY-Au	Au	7-hydroxy-6-methoxy-coumarin detection	[11]
	N/A	FAM-labeled chloramphenicol binding aptamer	Chloramphenicol detection	[225]
	Cu@GDY	Cu quantum dot	Hydroquinone and glucose detection	[27]
	Fe-MOF@GDY	Fe-based MOF	Chloramphenicol detection	[235]
	GDY-NH_2_	–NH_2_ group	Acetamide, benzamide, thioacetamide, and thiobenzamide detection (DFT calculations)	[236]
	GDY/Au oxide nanoparticles/luminol nanocomposite	Au oxide nanoparticles and luminol	IMP detection in meat products	[237]
	HsGDY@NDs	Nanodiamonds	Myoglobin and cardiac troponin I detection	[30]
	AuNPs/GDY; GDY@PANI/CdTe@ZnS QD; N/A	AuNPs; PANI and CdTe@ZnS QD; Cu^2+^	α-Syn detection	[172, 238, 239]
	GDQDs; N/A; N-doped GDY	N/A; carboxyl group; N	Dopamine detection	[226, 240–242]
	GDY@PEDOT:PSS	PEDOT:PSS	Uric acid detection	[243]
	CuPdPt NW-GDY	holothurian-shaped AuPd nanoparticles, TDNs and Cu-Pd–Pt nanowire networks	MCF-7 cell detection	[244]
Nucleic acid detection	GDYO; GDY nanosheet	FAM-labeled DNA	Fluorescence detection of DNA	[28, 80, 231]
	N/A	dCas9 and sgRNA	Genes analyzed in clinical samples from patients with Duchenne muscular dystrophy	[155]
	AuNPs-GDY; N/A	AuNPs; CRISPR/Cas12a	miRNA let-7a detection	[245, 246]
	N/A	AuNPs and GOx; AuNPs and aptamers	miRNA-21 and miRNA-141 detection	[147, 148]
	S-GDY; N/A	AuNPs and bovine serum albumin; AuNPs and bilirubin oxidase	miRNA-141 detection	[248, 249]
	N/A; AuNPs/GDY; GDY-Gr; AuNPs/GDY; g-C3N4/PDY; N/A	T7 exonuclease; AuNPs; graphene; AuNPs; g-C3N4 and porphyrin; AuNPs	miRNA-21 detection	[250–255]
	N/A	Ru@MOF, Ru-complex amine-rich nitrogen-doped carbon nanodots and Sm_2_S QD	CA19-9 detection (protein)	[32]
	HsGDY	ANXA2 antibody	ANXA2 detection (protein)	[214, 260]
	N/A	molybdenum disulfide, AuNPs and aptamer	HER2 detection (protein)	[261]
Antitumor	GDY/DOX	DOX and PEG	Chemotherapy and PTT	[25]
	FUGY	Fe_3_O_4_@UIO-66-NH_2_ and DOX	Magnetic targeting ability, imaging and chemotherapy	[165]
	BPFG/DOX	BP, FUGY and DOX	Photodynamic therapy and PTT	[228]
	GCDM	DOX, CDDP, and MTX	Imaging, photodynamic therapy and PTT	[102]
	GFR	RAP and FIN56	Ferroptosis and PTT	[12]
	GDYO	N/A	Inhibited lymphoma growth and reduced expression of inflammatory cytokines in CAFs; inhibited melanoma growth and activated cytotoxic T cells; promoted T cell secretion of inflammatory cytokines	[233, 262, 263]
	GDYO-STAT3	STAT3 protein crowns	Induction of immunosuppression of macrophage regeneration	[264]
	GDYO@i-RBM	i-RBM	Photodynamic therapy	[123]
	TTIS	Fe_3_O_4_	PAI, MRI, PTT and ferroptosis	[96]
	BN-GDY	B and N	Ferroptosis and apoptosis	[265]
	GDYO	N/A	Disrupted the actin cytoskeleton and caused apoptosis	[166]
	Na_2_S-GDY	Na_2_S	Ferroptosis and apoptosis	[266]
	DCM@GDY-CuMOF@DOX	DCM, CuMOF and DOX	Cuproptosis	[267]
	GDY-Cu	Cu^2+^	Reduced acetyl-CoA carboxylase and cytoplasmic acetyl-CoA synthetase levels	[232]
	CuO@GDY	CuO	Alleviated tumor hypoxia and caused Fenton-like reactions	[124]
	GDY-CeO_2_	CeO_2_ and miR181a	Alleviated tumor hypoxia and radiotherapy sensitization	[268]
Antimicrobial	GDY-modified TiO_2_ nanofibers	TiO_2_ nanofibers	Promoted bone tissue regeneration in drug-resistant bacterial infection	[224]
	AuAg/GDY@PEG	AuAg and PEG	Photothermal property, broad-spectrum antimicrobial activity, detection of *Salmonella typhi*	[272]
	ZnO@GDY NR	ZnO	Anti *Staphylococcus aureus* and *Pseudomonas aeruginosa*	[180]
	N-GDQDs/AuAg	N and AuAg	Antimicrobial efficacies against methicillin-resistant *Staphylococcus aureus* and *Escherichia coli*	[273]
	GDY@Ag nanoparticles	Ag	Antimicrobial activity *Bacillus subtilis* and *Escherichia coli*	[274]
	B-GDY	B	Antimicrobial activity against *Staphylococcus aureus* and *Escherichia coli*	[275]
	GDYO-loaded 3D-printed osteogenic scaffolds	N/A	Antimicrobial activity against *Staphylococcus aureus*	[271]
	PDMS@GDY@Cu	PDMS and Cu	Antimicrobial activity against *Escherichia coli* and *Streptococcus mutants*	[276]
Other biomedical applications	GDY-PEG	PEG	Enhanced the osteogenic differentiation of BMSCs	[279]
	GAS	Au, lithium and Na^+^	Integrate and output information from motor neurons	[280]
	N/A	minocycline	Crossed the blood–brain barrier in vivo, ameliorated behavioral deficits in dopaminergic mice, and restored the number of dopaminergic neurons to normal levels	[281]
	PEG-modified GDY	PEG	TRPV1 targeting, promoted neurotransmitter release and neural firing regulation	[282]
	GLA ointment	l-cysteine, Ag, methacryloyl gelatin and sodium alginate	Inhibited plaque biofilm and induced rapid remineralization of enamel	[283]
	MGMN	Manganese oxide nanoclusters	Eliminated pathogens, prevented biofilm formation, reduced inflammation, alleviated ocular hypoxia and promoted repair of corneal epithelial damage	[284]
	HsGDY	N/A	Skin patch sensor	[285]
	GDYO-modified sensing coil chip	N/A	Monitored respiratory patterns in epileptic rats in vivo	[286]
	GDY	N/A	Patch detection of blood pressure	[287]
	N/A	Polyhydroxyalkanoates and polyethylene glycol diacrylates	Promoted blood patency as a vascular graft	[288]
	Ag@GDY	Ag	Constructed ureteral stents with PLGA	[289]
	GDY/PCL	PCL	Stimulated the recovery of neuroelectric signals and promoted the regeneration of axonal myelin sheaths and vasculature	[290]

Modification and mode roles of GDY biomaterials in enzyme catalysis, molecular detection, nucleic acid detection, antitumor, antimicrobial, and sensor applications

### Enzymatic catalysis

Researchers prepared GDY nanoenzymes with catalase activity by modifying GDY. Wang et al. prepared a Hemin-GDY catalytic system by immobilizing Hemin on the surface of GDY. Hemin-GDY efficiently catalyzed the decomposition of H_2_O_2_ to produce hydroxyl radicals and superoxide anions. Moreover, the catalytic activity of Hemin-GDY was found to be 2.3-fold greater than that of immobilized Hemin on graphene, thereby demonstrating the superiority of GDY over graphene in terms of catalytic properties (Figure S15A) [24]. Zhu et al. similarly synthesized H/GDYO for the detection of H_2_O_2_ and glucose. The peroxidase activity of H/GDYO was found to be sixfold higher than that of Hemin (Figure S15B) [128]. Qi et al. prepared B-doped and keto group-rich GDY nanoenzymes with peroxidase activity. The results demonstrated that the catalytic activity of the prepared GDY nanoenzymes was approximately 4.2-fold higher than that of the B-free GDY. Furthermore, the GDY nanoenzymes produced in the study exhibited excellent sensitivity and selectivity for glucose [26]. Bai et al. synthesized a ZnO@GDY NR system with pressure-sensitive catalytic enzyme activity. This system possesses both intrinsic peroxidase activity and a strong piezoelectric response, and efficiently promotes the decomposition of H_2_O_2_ and the generation of reactive oxygen species (ROS) under ultrasound irradiation (Figure S15C) [180]. In comparison to the majority of carbon-based nanoenzymes, GDYO QDs exhibited a markedly elevated peroxidase activity, which can be attributed to the conjugated structure, oxygen-containing moiety, and smaller size of GDYO QDs. The linear range of GDYO QDs for H_2_O_2_ was 5–500 μM with a detection limit of 1.5 μM, and the linear range for cysteine was 0–90 μM with a detection limit of 0.48 μM [112]. Liu et al. immobilized Fe^2+^ and GOx on GDY nanosheets to proposed a GDY-based composite material (Fe-GDY/GOx) with dual enzyme activity. GDY demonstrated a robust adsorption capacity for GOx, and the adsorbed enzyme exhibited minimal structural alterations, yet the catalytic activity remained uncompromised (Figure S15D) [10]. This study also demonstrated that GDY exhibits a greater adsorption capacity for Fe^2+^ than graphene. This is attributed to the uneven energy distribution and *sp*-hybridized carbon atoms on the surface of GDY, which prevent Fe^2+^ from being oxidized and maintain the high catalytic activity of Fe^2+^.The prepared Fe-GDY/GOx was successfully utilized for one-step blood glucose detection, providing new insights into the application of GDY adsorption of metal ions and enzymes. Furthermore, the most recent study prepared Ru nanoparticles@GDYO (Ru@GDYO) with peroxidase activity by growing Ru nanoparticles in situ on GDYO nanosheets. Through characterization data, it was found that the charge transfer between Ru and GDYO in Ru@GDYO was limited, and the Ru nanoparticles deposited electroless on GDYO had a partially oxidized electronic structure, which contributed to the intrinsic physiological pH preference of peroxidase-mimicking nanoenzymes. It is of note that the activity of Ru@GDYO was considerably higher than that of Ru nanoparticles deposited on GO. This provides further evidence for the distinctive advantages of GDY materials [227].

### Molecular detection

Yan et al. developed an immediate detection method based on GDY for the rapid detection of rutin. The study prepared GDY-ionic liquid (GDY-IL) by ultrasonic preparation process, and then modified it on the surface of Au electrode by casting method. The results of the cyclic voltammetry test demonstrated that the GDY-IL composite on the electrode surface can effectively increase the electron transfer rate, which implies that the GDY-IL composite has a high conductivity with a large surface area. The modified electrode exhibited good detection performance for rutin with a wide linear range and low detection limit [234]. Hao et al. employed the property of GDY enabling strong π–π interactions and atomic dispersion of Fe sites with Hematin (HEM), while avoiding the formation of catalytically inactive dimers by HEM, to prepare GDY/HEM molecularly assembled materials. GDY/HEM exhibited an ultrafast response time of 0.95 s, a low detection limit of 7 nM and a wide linear range of 151.38 μM for nitric oxide [29]. Yan et al. prepared N-doped ultrathin GDY (NUGDY) electrodes for the detection of 6,7-dihydroxycoumarin using GDYO and melamine. The results demonstrated that NUGDY enhanced the electrode performance, and the electrochemical sensor designed in the study exhibited a high detection sensitivity for 6,7-dihydroxycoumarin [31]. In the most recent study, GDY-AuNPs nanocomposites were prepared using AuNPs grown on the surface of GDY and in between the layers. The linear detection of 7-hydroxy-6-methoxy-coumarin isolated from noni juice was found to be in the range of 10.0 nM to 1.0 mM, with a detection limit of 3.22 nM [11].

For the detection of environmental pollutants and food toxins, Yang et al. developed a fluorescence sensing platform for the detection of chloramphenicol, a food toxin, which employs the signal amplification property of RecJ(f) exonuclease and the adsorption capacity of nucleic acids of GDY material. The detection range for chloramphenicol was 10–80 μg/L, with a detection limit of 43.6 ng/L. The recoveries of chloramphenicol in food samples were 93.60–107.90% [225]. Niu et al. prepared Cu quantum dot-loaded GDY nanosheets (Cu@GDY) for the detection of organophosphorus pesticides. The results demonstrated that the Cu@GDY nanocomposites exhibited high sensitivity to organophosphorus pesticides, with a detection limit of 1 μg/L for hydroquinone. Furthermore, the Cu@GDY-based sensor exhibited high catalytic activity and excellent selectivity for the detection of glucose in an alkaline dispersed solution (Figure S16A) [27]. Besides, Zhang et al. prepared Fe-based MOF@GDY nanocomposites (Fe-MOF@GDY) with strong interfacial interactions. Fe-MOF@GDY exhibited a linear range of 1 pM to 24 mM for chloramphenicol, with a detection limit of 0.54 pM. Fe-MOF@GDY presented excellent reproducibility and stability, retaining 98.4% of its initial response after 3 weeks. A portable electrochemical microarray based on Fe-MOF@GDY was employed to accurately detect chloramphenicol in lake water, with recoveries ranging from 97.2 to 104.7% [235]. In contrast, Allangawi et al. analyzed the ability of the aminated GDY material (GDY-NH_2_) to form stable complexes with acetamide, benzamide, thioacetamide, and thiobenzamide by density functional theory (DFT) calculations yielded interaction energies of − 10.64, − 11.92, − 11.19, and − 13.28 kcal/mol, respectively, indicating the potential application of GDY-NH_2_ in toxicant detection [236]. Furthermore, researchers have developed a GDY/Au oxide nanoparticles/luminol nanocomposite for the detection of inosine monophosphate (IMP) in meat products. The linear detection range of GDY/Au oxide nanoparticles/luminol for IMP was from 0.01 g/L to 20 g/L, with a detection limit of 0.0013 g/L [237].

For protein detection, Wang et al. prepared a HsGDY@NDs nanocarrier system for myoglobin and cardiac troponin I detection. The data demonstrated that HsGDY@NDs exhibited high sensitivity for myoglobin and cardiac troponin I. The detection limits for myoglobin and cardiac troponin I were 6.29 and 9.04 fg/mL, respectively [30]. Studies have also developed several GDY biomaterials for α-Synuclein (α-Syn) detection [172, 238, 239]. Yao et al. prepared photoelectrochemical probes using a dopamine/4-mercaptophenylboronic acid/WSe_2_ (DA/MBA/WSe_2_) composite. Subsequently, α-Syn oligomer was converted into false-target DNA (FT), which formed a hairpin structure with triple-stranded DNA on AuNPs/GDY electrodes to recruit the photoelectrochemical probes and thus generate the corresponding photocurrent response [172]. The most recent report prepared N-doped GDY@polyaniline/CdTe@ZnS (GDY@PANI/CdTe@ZnS) QD sensing platform. The platform demonstrated a linear range of detection from 0.2 fM to 8 nM and a detection limit of 0.02 fM (S/N = 3) for α-Syn oligomers. Furthermore, it was capable of detecting α-Syn oligomers in human serum and dopaminergic neuronal cell lysates (Figure S16B) [238]. In contrast to the strategy of direct α-Syn detection, Jiang et al. employed the exceptional adsorption capacity of GDY for Cu^2+^ to achieve highly selective Cu^2+^ detection, which was utilized to assess the progression of Parkinson’s disease. The electrochemical sensor prepared in the study, based on GDY, effectively avoids interference from amino acids, metal ions, and neurotransmitters. It showed a high sensitivity of 9.77 μA/μM/cm^2^ for Cu^2+^, with a minimum detection concentration of 200 nM [239].

Interestingly, a portion of the research focused on the application of GDY materials to detect dopamine levels [226, 240–242]. Ahmed et al. demonstrated that GDQDs exhibit peroxidase activity. The detection limits for H_2_O_2_ and dopamine were obtained by colorimetric method as 0.13 μM and 8.65 μM, respectively [226]. In a separate study, a composite photovoltaic material comprising carboxylated GDY and TiO_2_ was synthesized. This sensor exhibited a wide linear detection range of 0.0005 mM to 1.05 mM and a low detection limit (1.36 × 10^–4^ mM) for dopamine [241]. Moreover, Cui et al. employed N-doped GDY nanostructures to achieve highly selective and sensitive dopamine detection with a linear range from 1 to 550 μM and a detection limit of 0.46 μM (Figure S16C) [242].

A recent study has reported the development of a graphdiyne@poly 3,4-ethylenedioxythiophene: poly styrene sulfonate (GDY@PEDOT:PSS) heterostructure that has been shown to achieve the lowest detection limit (6 nM), the widest detection range (0.03 µM-7 mM), and the longest stability (98.1% for 35 days) for uric acid detection performance. The authors posited that this enhanced detection performance could be attributed to the oxidative degradation resistance of GDY, which prevents the polymer from becoming less conductive due to water swelling and the π-π interactions that enhance the adsorption of uric acid (Figure S16D) [243]. Guo et al. innovatively applied holothurian-shaped AuPd nanoparticles, tetrahedral DNA nanostructures (TDNs), and interwoven Cu-Pd–Pt nanowire networks with graphdiyne sheet (CuPdPt NW-GDY) to design a platform capable of detecting MCF-7 breast cancer cells. The TDNs were found to be capable of accurately capturing circulating MCF-7 cells and could be coupled with CuPdPt NW-GDY coupling. The novel sensor demonstrated a linear range for MCF-7 cells of 10–10^6^ cells/mL, with an ultra-low detection limit of 7 cells/mL [244].

The molecular detection applications of GDY biomaterials encompass a vast array of studies at nearly every level, including molecular compounds, nucleic acids, proteins, and cells. The assays to be treated exhibit a remarkable linear range and low detection limits, which collectively illustrate the immense potential of GDY biomaterials for molecular detection applications. In light of the remarkable advancements made by GDY biomaterials in the field of nucleic acid detection, we will now delve into this subject in greater detail.

### Nucleic acid detection

Researchers have developed a series of sensors using GDY for the detection of nucleic acid molecules. Wang et al. co-incubated GDYO with FAM-labeled single-stranded DNA molecules based on the resonance energy transfer effect between GDYO and aromatic hydrocarbons. This resulted in the adsorption of GDYO and DNA molecules due to π-π interactions, which in turn caused fluorescence quenching of the FAM molecules labeled on the DNA. This was attributed to the energy resonance transfer effect. The addition of DNA complementary to single-stranded DNA to the incubation system resulted in a weakening of the base complementary pairing between the DNA molecules, attenuation of the energy resonance transfer effect, and restoration of the fluorescence of FAM molecules. Furthermore, GDYO exhibited superior sensitivity and a lower detection limit compared to GO on single-stranded DNA [80]. In addition, Parvin et al. demonstrated that GDY nanosheets with high fluorescence bursting ability and distinct affinities for double-stranded and single-stranded DNA enable in situ detection of DNA in homogeneous liquids [231]. Based on the aforementioned studies, Chang et al. successfully synthesized few-layer GDY nanosheets with a notable fluorescence burst effect for real-time detection of DNA by electrochemical doping with lithium. The GDY nanosheets exhibited high specificity, multiplicity, and ultra-high sensitivity. The researchers applied the sensing system for further detection of *mycobacterium tuberculosis* and identification of drug-resistant mutants in clinical samples, providing evidence for the clinical translational application of GDY [28]. Zheng et al. prepared a surface plasmon resonance sensor, CRISPR-SPR-Chip, by applying a GDY membrane conjugated with catalytically inactivated CRISPR-associated protein 9 (dCas9). The dCas9 protein and sgRNA complexes were immobilized on the surface of the GDY membrane and used to recognize and detect target sequences within the genomic DNA. Subsequently, the study employed the CRISPR-SPR-Chip sensor to analyze clinical samples from patients with Duchenne muscular dystrophy. The CRISPR-SPR-Chip yielded a clear positive result within 5 min, with 2 exon deletions of the target gene detected in the patient samples (Figure S17A) [155].

With the advancement of technology for the detection of single- and double-stranded DNA with GDY materials, researchers have expanded the scope of their work to include the detection of microRNA (miRNA), which have been identified as potential tumor markers. Li et al. employed the hole-electron pair generated by the natural band gap structure of GDY and the plasmon resonance effect of AuNPs to synthesize a photoactive GDY material loaded with AuNPs (AuNPs-GDY) for the highly sensitive detection of the tumor marker, miRNA let-7a [245]. Additionally, another study employed GDY as a foundation for a 3D DNA walkers-mediated CRISPR/Cas12a system, which significantly enhanced the sensitivity of the sensor. The linear range of miRNA let-7a detection by this system was 0.0001–10000 pM, with a detection limit of 8.11 aM and a signal to noise ratio (S/N) of 3 [246].

Notably, miRNA-21 and miRNA-141 were the most frequently detected as tumor markers [148, 247, 248]. Hou et al. developed a novel self-powered biosensing system for miRNA detection by using GDY as a substrate material for enzyme biofuel cell (EBFC). The results demonstrated that the detection limit of miRNA was 0.034 fM in the linear range of 0.1–100,000 fM [147]. In contrast, another study employed a sandwich GDY (S-GDY) material within the EBFC to enhance its performance. An external energy conversion device was utilized to coordinate signal amplification. The findings demonstrated that the EBFC exhibited a detection limit for miRNA-141 as low as 0.16 fM (S/N = 3) (Figure S17B) [248]. Xu et al. developed a self-powered biosensor for tumor marker detection by combining GDY and specific recognition of inducers. The results demonstrated that the biosensor exhibited detection limits of 0.15 fM and 0.30 fM for miRNA-21 and miRNA-141, respectively, in the linear ranges of 0.05–10000 fM and 1–10,000 fM [148]. In a surprising development, the study employed the DNA walker strategy to design GDY-based sensors for the detection of miRNA-21 or miRNA-141. The study employed a 3D DNA walker-mediated CRISPR/Cas12a cascade signal amplification strategy to construct a GDY-involved high-sensitivity EBFC, which is a platform with a detection line of 0.5–10,000 fM and a detection limit of 0.14 fM for miRNA-141 [249]. Additionally, study developed an ultrasensitive self-powered biosensing platform by applying GDY as the substrate material for electrodes. T7 exonuclease and a 3D DNA walker were then applied to trigger the rolling circle amplification reaction, which significantly improved the selectivity and sensitivity of the biosensor. The results demonstrated that the detection limits of miRNA-21 were 0.15 fM and 33 fM (S/N = 3) in electrochemical/colorimetric dual mode, respectively [250]. Similarly, another study designed a novel self-powered biosensor for more sensitive miRNA detection by integrating a DNAzyme walker and an AuNPs/GDY biosensing interface. The results demonstrated that the biosensor exhibited a detection limit of 0.015 fM (S/N = 3) for miRNA-21 over a linear range of 0.05 fM-10 pM (Figure S17C) [251].

Based on these findings, Xu et al. developed a self-powered biosensing platform by combining graphene/GDY/graphene (GDY-Gr) heterostructure with multiple signal amplification strategies. This approach was designed to further improve the detection sensitivity and obtain quantitative data using a smartphone. The results demonstrated that the detection limit of miRNA-21 was 0.03 fM in electrochemical mode. In colorimetric visualization mode, semi-quantitative visual in situ detection was achieved by analyzing gray scale values, with a detection limit of 32 fM. The sensor employed a multiple signal amplification strategy with 310-fold sensitivity enhancement, enabling highly sensitive quantitative and qualitative detection of liver cancer markers [252]. Shi et al. constructed a novel dual-mode self-powered biosensing platform based on the signal amplification strategy of AuNPs/GDY combined with DNA nanorings, which enabled the ultra-sensitive and specific detection of miRNA-21. The results demonstrated that the linear ranges of electrochemical and colorimetric detection modes of the biosensor were 0.1 fM-100 pM and 0.1 fM-10 nM, respectively, and the detection limits of miRNA-21 were 35.1 aM and 61.6 aM (S/N = 3) [253]. The most recent work developed a novel g-C3N4/porphyrin-based GDY (g-C3N4/PDY) photovoltaic nanocomposite which exhibited a 2.54-fold increase in the photoelectrochemical response compared to g-C3N4. It was demonstrated that g-C3N4/PDY was capable of detecting miRNA-21 with a linear range of 0.01 fM-100 pM and a limit of detection of 0.005 fM [254]. Moreover, Song et al. employed GDY and AuNPs-modified carbon paper as a substrate for EBFC, utilizing DNAzyme-mediated amplification of double-stranded displacement to demonstrate a linear relationship between 0.0001 pM and 10,000 pM for the detection of miRNA-21, with a sensitivity as low as 32.3 aM [255].

Nucleic acid detection technology has emerged as a pivotal instrument in various fields, such as the environment, biomedicine, pharmaceuticals, agriculture, and forensics. Advancements in materials science have led to improvements in nucleic acid detection technology towards augmented throughput, heightened resolution, and diminished cost [256, 257]. These innovations have engendered the identification of novel detection principles and the fabrication of new instrumentation, thereby propelling the application of nucleic acid detection [258, 259].

In addition to the detection of nucleic acid-based tumor markers, the study employed GDY to develop sensors for protein-based tumor markers. Bahari et al. developed a nano luminescent system based on GDY that is suitable for the detection of the tumor marker glycan antigen CA19-9. This provides a new idea for the detection of tumor markers [32]. Chauhan et al. prepared HsGDY nanosheets by conjugating Annexin A2 (ANXA2) antibody to the GDY surface. The linear detection of ANXA2 by HsGDY ranged from 0.01 fg/mL to 1000 ng/mL, and the electrochemical results obtained demonstrated a high degree of correlation with the concentrations of ANXA2 cancer biomarkers in patients obtained by enzyme-linked immunosorbent assay [214]. A subsequent study by this author utilized HsGDY loaded with ANXA2 antibody to successfully adjust the lower limit of detection of ANXA2 to 100 fg/mL. The immunoassay demonstrated high accuracy for the detection of ANXA2 in serum samples from hepatocellular carcinoma patients, with a wide range of ANXA2 concentrations (100 fg/mL-100 ng/mL) covered (Figure S17D) [260]. Furthermore, heterostructures of GDY and tetragonal molybdenum disulfide were prepared for the highly sensitive detection of HER2. These heterostructures exhibited an extended linear response range of 0.1–10,000 pg/mL and a low detection limit of 0.03 pg/mL (S/N = 3) [261].

### Antitumor

The considerable specific surface area and fully conjugated structure confer upon GDY a remarkable capacity for drug carriage. The feasibility of constructing a nanodelivery system using GDY, and the antitumor effects and molecular mechanisms of the GDY nanodelivery system were investigated. In the initial GDY material antitumor study, Jin et al. developed a drug delivery system, GDY/DOX, based on GDY nanosheets using DOX as a model drug for combined photothermal/drug therapy in living mice. The aromatic hydrocarbon structure of DOX caused it to be adsorbed on the surface of GDY nanosheets, and GDY/DOX exhibited a high photothermal conversion ability and drug release efficiency under 808 nm laser irradiation, which demonstrated a more pronounced inhibition effect on tumor cell growth. Furthermore, histopathological examinations and serum biochemical analyses demonstrated that GDY exhibited excellent biocompatibility and did not elicit any discernible adverse effects [25]. Xue et al. developed a FUGY tumor-targeting drug delivery system with magnetic targeting ability. The authors then loaded DOX onto FUGY since DOX can be used both as an anticancer agent and as a fluorescent probe to determine the location of FUGY. The results demonstrated that FUGY, with a drug loading capacity of up to 43.8%, was capable of effectively releasing DOX around tumor cells. Furthermore, the FUGY delivery system exhibited superior biocompatibility, sustained drug release, and targeting compared to the direct administration of free DOX in vitro and in vivo [165]. Furthermore, Lin et al. prepared BODIPY-PEG-Fe_3_O_4_@UIO-66-NH_2_/DOX (BPFG/DOX) nanoparticles by encapsulating DOX-loaded FUGY in phosphatidylcholine and fluorescent organic polymer BODIPY-PEG (BP). The π-π interaction between GDYO and BP in this BPFG/DOX nanoparticle enhanced the efficacy of photodynamic therapy. Following irradiation with an 808 nm laser, the viability of MCF-7 cells treated with approximately 10 μg/mL of BPFG/DOX was reduced to a level approaching 90% [228]. Xing et al. prepared a multifunctional nanomedicine GCDM, for diagnostic and targeted cancer photochemistry synergistic therapy. This was achieved by loading DOX, CDDP, and MTX on the GDYO surface. GCDM demonstrated synergistic antitumor effects and reduced tumor resistance to three conventional anticancer drugs, exhibiting photothermal conversion efficiency (47%) and good photodynamic effects under NIR irradiation. Furthermore, GCDM exhibited good biocompatibility, in vivo targeting, long-term retention, sustained drug release, good fluorescence imaging capability, and significant photochemical synergistic therapeutic effects [102]. In our previous work, we encapsulated a receptor-associated protein (RAP) polymeric peptide and the ferroptosis inducer FIN56 on GDY to design a GDY-FIN56-RAP (GFR) nanoplatform with a PTT effect for GBM therapy. The GFR nanoplatform combined a precise drug delivery system, the photothermal properties of GDY, and the ferroptosis sensitivity of GBM cells, offering a promising approach to the treatment of GBM (Figure S18A) [12].

In their study of tumor immune microenvironment regulation, Li et al. investigated the regulatory effects of GDYO on the tumor microenvironment (TME) of lymphoma. Their findings revealed that GDYO not only directly inhibited the growth of numerous types of lymphoma cells, but also reduced the expression of inflammatory cytokines in cancer-associated fibroblasts (CAFs). The study found that GDYO inhibits Mif-Ackr3 signaling in tumor cells and CAFs, reducing the number of regulatory T cells and tumor stem cells in TME, and ultimately inhibiting lymphoma growth (Figure S18B) [262]. A further study demonstrated that GDYO not only resulted in a direct reduction in tumor growth in a mouse model of melanoma, but also activated cytotoxic T-cells, either directly or indirectly via macrophages. This enhanced the response to checkpoint inhibitors in a breast cancer model [263]. Besides, research also revealed that GDYO enhanced the mechanical stiffness of oral cancer cells under laser irradiation in vitro, augmented the cytotoxicity of T cells and the secretion of inflammatory cytokines, and exhibited an inhibitory effect on oral cancer. These findings offer a novel perspective on the antitumor therapy of GDYO [233]. Guo et al. prepared GDYO nanosheets-STAT3 protein crowns nanocomposite (GDYO-STAT3) to induce regeneration of immunosuppressive macrophages. Furthermore, the GDYO-STAT3 interaction was observed to elicit immune responses within the TME, thereby facilitating cancer immunotherapy. This finding offers a novel approach for the utilization of GDY materials in tumor immunotherapy [264].

The unsaturated carbon atoms in GDY facilitate covalent and non-covalent modification. GDY derivatives with special physicochemical properties can be prepared by doping heteroatoms (e.g., O, B, or N, etc.) or anchoring metal atoms (e.g., Fe or Ni, etc.) to exert antitumor effects. It was demonstrated that GDYO nanosheets could catalyze the oxidation of water, resulting in the release of O_2_, and combining with NIR irradiation to produce ^1^O_2_. Additionally, GDYO nanosheets exhibited excellent photothermal conversion performance, with a photothermal conversion efficiency of 60.8%. Accordingly, biomimetic GDYO@iRGD peptide-modified red blood cell membranes (i-RBM) nanosheets (GDYO@i-RBM) targeting tumors were prepared by coating i-RBM on GDYO nanosheets. The resulting GDYO@i-RBM nanosheets demonstrated the capacity to enhance tumor reoxygenation and perfusion in photodynamic therapy [123]. Hydroxyl radicals represent another form of ROS, and Fe^2+^/Fe^3+^ can facilitate the catalyze endogenous H_2_O_2_ to hydroxyl radicals in the Fenton reaction. Nevertheless, the efficiency of the Fenton reaction in tumor tissues is frequently constrained by the influence of the TME. Min et al. devised a TTIS nanocomposite with high affinity for Fe. TTIS was accumulated in tumor tissues through tumor-targeting polymer modifications, which enables tumor PAI and MRI. TTIS has an excellent photothermal conversion efficiency (37.5%), which makes it a highly efficient PTT agent. Moreover, the heat generated during the PTT process accelerated the release of Fe from TTIS, thereby enhancing the efficiency of the Fenton reaction and enabling PTT and Fenton-mediated tumor combination therapy (Figure S18C) [96]. Zhang et al. reported a B and N conjugated GDY nanosheets (BN-GDY) with a highly efficient GSH depletion capacity. BN-GDY induced ferroptosis by inhibiting GPX4 activity and induced apoptosis by down-regulating Bcl2 in colorectal cancer cells [265]. Wang et al. discovered that genes involved in cell adhesion were significantly enriched in DNMT3A-mutated acute myeloid leukemia cells. Furthermore, GDYO demonstrated specific cytotoxicity against DNMT3A-mutated cells, exerting an anti-leukemic effect. Mechanistically, GDYO directly interacted with integrin β−2 and c-type mannose receptors to promote GDYO adsorption and cellular uptake. In addition, GDYO bound to actin and prevented actin polymerization, thereby disrupting the actin cytoskeleton and ultimately leading to apoptosis [166]. Wang et al. synthesized metal-free doped Na_2_S-GDY nanoenzymes with GDY and Na_2_S with piezoelectric properties. Ultrasonic excitation enhanced peroxidase mimetic activity, and the Na_2_S-GDY nanoenzymes converted H_2_O_2_ into hydroxyl radicals and induced apoptosis in 4T1 cells. Furthermore, the ultrasound-enhanced nanoenzymes disrupted the redox balance by promoting GSH depletion and GPX4 inactivation, inducing ferroptosis in 4T1 cells [266].

The most recent study developed a DU145 cell membrane (DCM)@GDY-Cu-based MOF (CuMOF)@DOX biomimetic nanoplatform (DCM@GDY-CuMOF@DOX) for targeting prostate cancer based on GDY and CuMOF by loading DOX and coating DCM on the surface. The platform released DOX and generated Cu^+^ in the presence of GSH, effectively inhibiting tumor growth in vivo by generating lethal ROS and mediating cuproptosis (Figure S18D) [267]. Similarly, another study capitalized on the toxicity of Cu ions by utilizing GDY as a template for immobilizing Cu ions. The resulting nanocomposite, GDY-Cu, demonstrated inhibitory effects on the synthesis of fatty acids and lipid metabolism by reducing acetyl-CoA carboxylase and cytoplasmic acetyl-CoA synthetase levels in DU145 cells. This resulted in the inhibition of tumor growth [232].

The malignant process of tumors and the ischemia caused by antitumor therapy exacerbate the hypoxia of solid tumors, thereby increasing the difficulty of treatment. Therefore, researchers constructed copper oxide@GDY (CuO@GDY) nanocatalysts that exhibited efficient and controllable photocatalytic oxygen production under NIR laser irradiation. This approach has the potential to alleviate tumor hypoxia. Furthermore, the CuO@GDY nanocatalysts catalytically generated ROS in response to endogenous H_2_O_2_-accelerated Fenton-like reactions via X-ray excitation, enabling the selective killing of tumor cells over normal cells [124]. Zhou et al. immobilized CeO_2_ nanoparticles on the surface of GDY and designed a GDY-CeO_2_ nanocomposite with sensitization for radiotherapy. The GDY-CeO_2_ nanocomposite decomposed endogenous H_2_O_2_ into O_2_, alleviating tumor hypoxia and promoting radiation-induced DNA damage. This ultimately inhibited esophageal tumor growth in vivo. Furthermore, the authors loaded miR181a onto GDY-CeO_2_ and delivered miR181a to tumor tissues via iRGD-targeted polymer modification. miR181a exerted radiotherapy sensitization by directly targeting the cell cycle checkpoint protein RAD17 and regulating the DNA damage checkpoint kinase Chk2 pathway [268]. A comprehensive examination of GDY has revealed that materials designed using the photothermal and readily modifiable properties of GDY offer a valuable reference point for more effective solid tumor treatment.

The research of GDY materials in tumor diagnosis and treatment primarily employed its remarkable drug-carrying capacity and photothermal properties. However, the strong affinity of GDY for transition metal ions has not been extensively utilized. Additionally, although it has been demonstrated that GDYO can serve as a novel type of carbon nanoenzymes that mimic peroxidases, there are only a limited number of studies on metal-doped GDY materials in this field [269]. Moreover, the tumor-targeting and responsiveness properties of the materials represented significant limitations to their application and development. The key to promoting the application of GDY in tumor diagnosis and treatment is to design GDY and its derivatives to enhance the therapeutic effect and expand the therapeutic modalities and targets.

### Antimicrobial

In summary, the antimicrobial properties of GDY biomaterials are contingent upon their peroxidase activity, ROS generation, or photothermal properties [224, 270, 271]. The capacity of GDY to impede the proliferation of a diverse range of bacteria has been demonstrated to render GDY-modified TiO_2_ nanofibers an optimal choice for use in biomedical applications. These nanofibers exhibit excellent biocompatibility and osteoinductive properties, facilitating cell adhesion and differentiation, thereby contributing to the process of bone tissue regeneration in implant infections induced by drug-resistant bacteria [224]. Bai et al. prepared AuAg nanocages/GDY@PEG (AuAg/GDY@PEG) composites by combining AuAg and GDY. The AuAg/GDY@PEG composites exhibited a strong photothermal effect and demonstrated excellent broad-spectrum antimicrobial activity in vivo, with a killing rate of greater than 99.999%. Furthermore, the study established a simple photothermal immunoassay for pathogenic bacteria based on the photothermal conversion ability of AuAg/GDY@PEG, with a detection limit of 10^3^ CFU/mL for *Salmonella typhi* in the range of 10^3^–10^7^ CFU/mL. This assay can be used for the on-site detection of pathogenic bacteria in food (Figure S19A) [272]. Moreover, another work by this author resulted in the preparation of ZnO@GDY NR, which demonstrated remarkable antimicrobial efficacy in vitro and in vivo against multidrug-resistant pathogens, including *Staphylococcus aureus* and *Pseudomonas aeruginosa*. The objective of this study was to apply the prepared ZnO@GDY NR to develop a nano-enzymatic skin patch with favorable hemocompatibility and cytocompatibility for expeditious skin wound disinfection [180]. Similarly, another study prepared hollow cube-like N-doped GDYO quantum dots/AuAg (N-GDQDs/AuAg) nanocage heterostructures by loading N-doped GDYO quantum dots on AuAg nanocages. This significantly enhanced their peroxidase-like activity under 808 nm irradiation, and their antimicrobial efficacies against methicillin-resistant *Staphylococcus aureus*, *Staphylococcus aureus*, and *Escherichia coli* at greater than 99% (Figure S19B) [273]. Furthermore, the antibacterial potential of GDY materials has been investigated by synthesizing GDY@Ag nanoparticles without the use of reducing agents. The results demonstrated that GDY@Ag nanoparticles kill bacteria by disrupting membranes and generating ROS, and exhibits high stability and broad-spectrum antimicrobial activity against both Gram-positive and Gram-negative bacteria, without inducing bacterial resistance [274]. In addition to the metal-doped structures, study also constructed B-doped GDY nanosheets (B-GDY), which greatly improved the bactericidal effect against Gram-positive and Gram-negative bacteria by promoting the decomposition of H_2_O_2_ to produce ROS (Figure S19C) [275]. Wang et al. devised an innovative method for preparing GDYO-loaded 3D-printed osteogenic scaffolds. These scaffolds exhibited 90.18% antimicrobial activity against *Staphylococcus aureus* following NIR laser irradiation, while maintaining good tissue-organ compatibility (Figure S19D) [271]. Additionally, Hu et al. prepared antimicrobial sponges polydimethylsiloxane (PDMS)@GDY@Cu (PDMS@GDY@Cu) through the in situ generation of GDY on Cu sponge, followed by the modification of PDMS. The tight coverage of GDY on Cu sponge significantly enhanced its anticorrosive property and antimicrobial activity. The self-cleaning behavior and photothermal-assisted antimicrobial property of PDMS@GDY@Cu were well maintained under prolonged bacterial attack [276].

### Other biomedical applications

In addition to the aforementioned applications, GDY materials are being utilized in neuroscience, wearable technology, and implant research. In these studies, data on the safety of GDY materials are also discussed in detail. Studies have demonstrated that GDY at concentrations up to 100 μg/ml can exert cytotoxic effects on HUVEC, whereas low doses of GDY have no significant impact on cell viability [33]. Zheng et al. demonstrated that GDYO was more readily soluble in a variety of media than GO. Furthermore, no significant kinetic aggregation was observed when its concentration was increased by 24 h incubation in saline, phosphate buffer, and cell culture medium. Furthermore, GO nanoparticles adhered and aggregated on the surface of human liver cell membranes, resulting in the occurrence of cell membrane ruffling and apoptosis and inducing ROS production by the cells. However, GDYO did not adhere to the surface of human liver cell membranes to produce similar effects. Additionally, there was no erythrocyte killing effect of both GDYO and GO in the experimental conditions. This study establishes the foundation for carrying out in vivo and in vitro applications of GDY materials [277]. Subsequently, Bengt et al. demonstrated that GDYO has minimal cytotoxicity against classically activated (M1) and alternatively activated (M2) macrophages using human primary monocyte-derived macrophages as a model. Additionally, GDYO reprogrammed M2 macrophages into M1 macrophages and GDYO was degraded in M1 macrophages in an inducible nitric oxide synthase-dependent manner. This study provides new insights into the interaction between GDYO and human macrophages, offering new avenues for tumor immunotherapy [278]. Moreover, Li et al. discovered that a low concentration of GDY-PEG markedly enhanced the osteogenic differentiation of bone marrow mesenchymal stem cells (BMSCs). The optimal concentration of GDY-PEG treatment was identified as 1 μg/mL. Additionally, the regulatory impact of GDY-PEG on the osteogenic differentiation of BMSCs may be associated with the Wnt/β-catenin signaling pathway [279]. A recent study of GDY biosafety compared the developmental toxicity of GO and GDY to zebrafish larvae. The results demonstrated that exposure of zebrafish embryos to GO and GDY for up to five days post-fertilization resulted in reduced hatching rates, morphological malformations, and hyperactivity of the larvae. However, there was no significant effect on blood flow rate. Notably, GO induced greater toxic effects than GDY at the same dose, suggesting a potential biocompatibility advantage of GDY over GO [34].

Based on the excellent electrical signaling properties of the GDY material, Wei et al. developed a GDY-based artificial synapse (GAS), which is capable of processing signals transmitted from multiple preneurons in parallel. To simulate artificial neural loops, the researchers connected the GAS to artificial muscles. The data demonstrate that this GAS-simulated nerve is capable of integrating information from preneurons and outputting information from motor neurons, which presents a novel solution for neural robotics and bio-hybrid systems for brain-machine interfaces [280]. Li et al. prepared a GDY nanodelivery platform loaded with minocycline, achieving approximately 90% drug loading and 30% NIR-induced drug release. Furthermore, the platform demonstrated the ability to traverse the blood–brain barrier in vivo, thus enabling the correction of behavioral deficits in dopaminergic mice and the restoration of dopaminergic neuron counts to normal levels [281]. A recent study used PEG-modified GDY to create a nanosensor targeting the temperature-sensitive TRPV1 channel on neuronal cell surfaces. The sensor demonstrated high photothermal conversion efficiency in NIR and strong TRPV1 targeting. Photothermal activation of TRPV1 led to neurotransmitter release and nerve firing modulation in live mice (Figure S20A) [282].

Liao et al. employed a ligand reduction strategy driven by L-cysteine and GDY to anchor low-dose Ag nanoparticles/Ag^+^ onto GDY nanosheets, thereby obtaining GDY/L-cysteine/Ag (GLA) nanoenzymes. GLA exhibited acidic plaque biofilm-activated peroxidase-like activity, which can be employed in a synergistic manner to inhibit plaque biofilm formation on human teeth by concurrently enhancing the release of Ag^+^ from acidic plaque biofilms. It is also noteworthy that the authors encapsulated GLA into an ointment composed of methacryloyl gelatin and sodium alginate, resulting in a biodegradable and viscous GLA ointment that can act as a template nucleation site, cross-linking with abundant Ca^2+^, thereby attracting salivary PO_4_^3−^ and inducing the growth of hydroxyapatite on the enamel, promoting rapid remineralization [283]. In another study, manganese oxide nanoclusters/GDY/hyaluronic acid and polymethylmethacrylate-based ophthalmic microneedles (MGMN) were prepared. The MGMN’s polymerase-like activity period has been demonstrated to exert antimicrobial and anti-inflammatory effects in a series of in vitro, in vivo, and ex vivo experiments. These effects include the elimination of pathogens, prevention of biofilm formation, reduction of inflammation, alleviation of ocular hypoxia, and promotion of repair of corneal epithelial damage (Figure S20B) [284].

Furthermore, Cai et al. developed a HsGDY nanofilm with a conjugated porous structure and intrinsic softness for use as a skin patch sensor. The engineered HsGDY sensor demonstrated consistent and precise results with high sensitivity, rapid response, and long-term durability under minimal skin deformations, offering a novel concept for wearable organic bioelectronic devices [285]. In the most recent study, the researchers reported the development of an electrochemical sensor that is capable of accurately tracking respiratory patterns in a small animal model. This is achieved by wirelessly coupling a GDYO-modified sensing coil chip to a reader coil chip. The sensor perturbed the proton transport at the GDYO interface of the sensing coil chip by applying an alternating current through the reader coil chip in electrochemical impedance measurement mode. The high-frequency perturbation condition significantly reduced the interfacial resistance to proton transport by 5 orders of magnitude at 95% relative humidity and improved the low humidity response with a detection limit as low as 0.2% relative humidity, enabling precise in vivo profiling of respiratory patterns in epileptic rats [286]. Besides, Lu et al. employed the out-of-plane ion transfer facilitated by the triangular pores in the GDY structure to fabricate a flexible sensor with exceptional performance, resulting in a pore utilization rate of 99.3%. This enables precise patch detection of blood pressure (Figure S20C) [287].

Given the extensive range of potential applications and significant potential of hydrogels in the biomedical field, Hou et al. prepared amphiphilic and fatigue-resistant organhydrogels by combining polyhydroxyalkanoates, polyethylene glycol diacrylates, and GDY. The organhydrogels demonstrated remarkable stability in body fluids, retaining their tensile modulus over 2000 cycles of stretching. Following transplantation into the body, the vascular grafts demonstrated notable cellular infiltration and tissue regeneration. Additionally, the blood patency rate was observed to be higher than that of the control group at the three-month mark [288]. Zhang et al. synthesized Ag nanoparticles@GDY (Ag@GDY) by in situ reduction method. A biodegradable ureteral scaffold was constructed by homogeneous mixing of the synthesized and purified Ag@GDY with polylactic acid-glycolic acid (PLGA) as an antimicrobial agent using electrostatic spinning. Results demonstrated that the scaffold exhibited effective anti-biofilm properties, variable mechanical properties, and satisfactory biocompatibility [289]. Li et al. prepared a GDY-loaded polycaprolactone (PCL) composite scaffold (GDY/PCL) using electrostatic spinning technology and evaluated the biocompatibility of the scaffold in a peripheral nerve injury model. The results demonstrated that GDY/PCL could promote the proliferation and differentiation of neuronal cells, stimulate the recovery of neuroelectric signals, and promote the regeneration of axonal myelin sheaths and vasculature, which ultimately led to good nerve regeneration and motor function. Furthermore, the results indicated that GDY/PCL exhibited good histocompatibility. This study demonstrates the significant potential of GDY nanomaterial scaffolds in the field of nerve regeneration, with promising clinical translation prospects (Figure S20D) [290].

## Challenges and opportunities for GDY biomedical applications

Although researchers have made significant strides in the fields of drug delivery, biosensing, enzyme catalysis, antimicrobial, and cancer research using GDY biomaterials, there are still challenges and opportunities for improvement. A review of the cases presented above reveals that further investigation is required in order to ascertain the stability of GDY biomaterials in the in vivo environment and the controllability of drug release in drug delivery studies. In addition to the detection of drug release under different temperatures, pH, and other conditions [99–101], it is also necessary to conduct in vivo pharmacokinetic analyses. These analyses should include not only the pharmacokinetic study of the GDY-loaded cargo, but also the GDY material metabolism analysis of the GDY material itself. This is necessary in order to ensure the safety and efficacy of GDY biomaterials in practical applications. In addition, the laboratory conditions established by the authors for the study of sensors often prove challenging to replicate in complex biological environments [27, 31, 128, 239]. The methods for maintaining the stability and selectivity of sensors in such environments require further optimization.

Moreover, all studies on living organisms should analyze in great depth both the biocompatibility and long-term safety of GDY biomaterials in vivo, with the aim of preventing potential side effects. It would be a mistake to limit the scope of such studies to detecting the observation of pathological damage to major organs and the assessment of hepatic and renal functions after short-term administrations of treatments [25, 96, 165]. It is important to note that in combination therapy programs, especially those involving PTT, it is crucial to monitor temperature alterations with greater precision within the lesion. In contrast, the current study merely observed temperature fluctuations at the surface of the skin [12, 96, 265]. Although 0.5, 1, or 2 W/cm^2^ of NIR excitation is frequently employed in the literature to stimulate the photothermal properties of GDY biomaterials [102, 124, 265], there is currently a lack of data to assess whether the depth and range of light exposure can be controlled, or whether there is any damage to normal tissues. Consequently, it is of paramount importance that future research focuses on the targeted and efficient delivery of PTT, whilst simultaneously reducing damage to normal tissues. This will necessitate the implementation of more rigorous screening procedures to identify disease-specific targets.

It is noteworthy that GDY biomaterials, and indeed other nanomaterials, encounter a significant obstacle in their transition from laboratory to clinical applications, as the complexity of drug development and the sophistication of drug review systems render this process increasingly challenging [291, 292]. Even drugs currently in use in clinical settings are continually uncovering new therapeutic mechanisms and identifying novel diseases for which they can be used [293]. Nanomaterial drugs, which are permitted for use in therapy due to their physical and chemical properties of being “not drugs” but “materials” necessitate further investigation. However, this does not mean that nanomaterial drug research is useless, because the multitude of unsolvable diseases in living organisms makes all drug development of the most fundamental importance, and we cannot know whether the drugs that will cure diseases in the future will be “drugs” or “non-drugs”.

Therefore, the use of GDY for non-invasive body applications such as molecular detection, enzyme catalysis, and antimicrobials seems more feasible currently. Even in the future, GDY may also adopt a similar development model to graphene, including skin care, topical heat therapy, glucometers and wearable devices [294–296]. However, in comparison to graphene, GDY’s intricate preparation process and comparatively limited mass production have constrained its advancement. Consequently, to date, GDY has been most extensively utilized in the domains of energy, catalysis, water treatment, humidity sensors, and related fields [297–299]. It is of interest to ascertain whether the biomedical applications of GDY will experience a surge in prominence or remain relatively dormant in the future.

## Conclusions and perspectives

This review highlights the significant progress made in the biomedical applications of GDY materials, and the characterization techniques and research strategies for GDY biomaterials are similarly highlighted in comparison to similar review articles. The detailed description of the characterization techniques for GDY biomedical applications not only guides the researchers to choose the methodologies quickly and accurately, but also provides a guarantee of the accuracy of the data for GDY biomaterials. It is important to note that the characterization techniques discussed in this review are not isolated or unrelated to one another. In fact, studies often or must apply more than two methods to characterize the physicochemical properties of GDY biomaterials. This is exemplified by the correlation between morphology, particle size, dispersibility, and zeta potential data, which is discussed in the text. The researcher employs these data to substantiate the reproducibility of the GDY biomaterial preparation, whereas the reader or reviewer is tasked with evaluating the authenticity and reliability of the study based on the comparison between multiple data sets. This is also true of modified characterization techniques. Consequently, our work aims to provide researchers and readers or reviewers with a baseline standard to ensure the authenticity of current and future GDY biomaterial development processes.

It is evident that biomedical researchers frequently lack a certain degree of research foundation in materials science or engineering. Consequently, the design and preparation of GDY biomaterials, as well as the planning of research content to achieve research objectives, represent key limitations to biomedical research. Consequently, we present here research strategies with general reference value, which are intended to guide researchers in the design of reasonable preparation strategies for GDY biomaterials and the selection of appropriate characterization techniques. These strategies are based on typical cases that have been reported in order to explore the potential of GDY for biomedical applications. It is also noteworthy that numerous studies exhibit considerable overlap. For instance, in the enzyme catalysis study of GDY materials, numerous heterostructures were prepared with peroxidase activity and subsequently employed for antimicrobial or molecular detection [180, 226, 275]. This suggests that researchers need not restrict their work to a single area, such as enzyme catalysis or antimicrobial, and that it is worthwhile to advocate for the exploration of GDY applications in multiple directions. In fact, the characterization techniques and preparation strategies of GDY biomaterials described in this review are also applicable to other 2D sheet materials, such as graphene, black phosphorus, some perovskites, and B nitride nanosheets, etc. Researchers may find this article useful as a source of inspiration and guidance according to the purpose of their research.

In conclusion, this review details the material properties of GDY, analyzes specific parameters in GDY biomedical applications, and innovatively summarizes characterization techniques used in GDY biomedical applications. It also proposes biomedical research strategies for GDY and its derived materials. This integrated guide to biomedical research on GDY and its derived materials provides a comprehensive overview of the subject matter. It is of significant importance to note that further multidisciplinary cross-sectional studies are necessary not only for GDY materials but also for the advancement of various fields in biomedicine.

## Supplementary Information

Supplementary Material 1.

## Data Availability

No datasets were generated or analysed during the current study.

## Abbreviations

AFM: Atomic force microscopy
Ag: Silver
Ag@GDY: Ag nanoparticles@graphdiyne
ANXA2: Annexin A2
AuAg/GDY@PEG: AuAg nanocages/graphdiyne@polyethylene glycol
AuNPs: Gold nanoparticles
AuNPs-GDY: Graphdiyne material loaded with gold nanoparticles
B: Boron
B-GDY: B-doped graphdiyne nanosheets
BMSCs: Bone marrow mesenchymal stem cells
BN-GDY: B and N conjugated graphdiyne nanosheets
BP: BODIPY- polyethylene glycol
BPFG/DOX: BODIPY-PEG-Fe_3_O_4_@UIO-66-NH_2_/doxorubicin
C/O: Carbon/oxygen ratio
CAFs: Cancer-associated fibroblasts
CDDP: Cisplatin
Cu@GDY: Cu quantum dot-loaded graphdiyne nanosheets
CuMOF: Cu-based metal–organic framework
CuO@GDY: Copper oxide@graphdiyne
CuPdPt NW-GDY: Interwoven Cu-Pd–Pt nanowire networks with graphdiyne sheet
DA/MBA/WSe_2_: Dopamine/4-mercaptophenylboronic acid/WSe_2_
dCas9: CRISPR-associated protein 9
DCM: DU145 cell membrane
DCM@GDY-CuMOF@DOX: DU145 cell membrane@graphdiyne-Cu-based MOF@doxorubicin biomimetic nanoplatform
DFT: Density functional theory
DLS: Dynamic light scattering
DOX: Doxorubicin
EBFC: Enzyme biofuel cell
EDS: Energy dispersive spectroscopy
EE%: Encapsulation efficiency
F: Fluorine
Fe: Iron
Fe-MOF@GDY: Fe-based metal–organic framework@graphdiyne nanocomposites
FNA: Framework nucleic acid
FP: Fluorescence polarization
FT: False-target
FTIR: Fourier transform infrared spectroscopy
FUGY: Graphdiyne-coated Fe_3_O_4_@UIO-66-NH_2_
GAS: Graphdiyne-based artificial synapse
GBM: Glioblastoma
GCDM: Graphdiyne oxide-cisplatin/doxorubicin@1,2-Distearoyl-sn-glycero-3-phosphorylethanolamine-polyethylene glycol-methotrexate
GDQDs: Graphdiyne quantum dots
GDY: Graphdiyne
GDY@PEDOT:PSS: N-doped graphdiyne@polyaniline/CdTe@ZnS
GDY@PANI/CdTe@ZnS: Graphdiyne@poly 3,4-ethylenedioxythiophene: poly styrene sulfonate
GDY/GO/Ag/Ag: Graphdiyne crosslinked with graphene oxide and hybridized with silver/silver bromide
GDY/PCL: Graphdiyne-loaded polycaprolactone composite scaffold
GDY/Pt: Graphdiyne and Pt nanoparticles
GDY-Gr: Graphene/graphdiyne/graphene
GDY-IL: Graphdiyne-ionic liquid
GDY-NH_2_: Aminated graphdiyne material
GDY-PEG: Polyethylene glycol-modified graphdiyne
GDYO: Graphdiyne oxide
GDYO QDs: Graphdiyne oxide quantum dots
GDYO@i-RBM: Graphdiyne oxide@iRGD peptide-modified red blood cell membranes nanosheets
GDYO-CDDP: Cisplatin-modified graphdiyne oxide nanosheets
GDYO-STAT3: Graphdiyne oxide nanosheets-STAT3 protein crowns nanocomposite
GFR: Graphdiyne-FIN56-receptor-associated protein
GLA: Graphdiyne/l-cysteine/Ag
GNTs: Graphyne nanotubes
GO: Graphene oxide
GOx: Glucose oxidase
H: Hydrogen
H/GDYO: Hemin/graphdiyne oxide
HEM: Hematin
HsGDY: Hydrogen-substituted graphdiyne
HsGDY@NDs: Nanodiamonds and hydrogen-substituted graphdiyne isomers
HUVEC: Human umbilical vein endothelial cells
ICP-MS: Inductively coupled plasma mass spectrometry
IMP: Inosine monophosphate
Ir: Iridium
i-RBM: IRGD peptide-modified red blood cell membranes
LC%: Loading content
LC–MS: Liquid chromatography-mass spectrometry
LSPR: Localized surface plasmon resonance
MGMN: Manganese oxide nanoclusters/graphdiyne/hyaluronic acid and polymethylmethacrylate-based ophthalmic microneedles
miRNA: microRNA
MOF: Metal–organic framework
MRI: Magnetic resonance imaging
MTX: Methotrexate
N: Nitrogen
NDs: Nanodiamonds
N-GDQDs/AuAg: N-doped graphdiyne oxide quantum dots/AuAg
Ni: Nickel
NIR: Near-infrared region
NUGDY: N-doped ultrathin graphdiyne
O: Oxygen
P: Phosphorus
PAI: Photoacoustic imaging
PCL: Polycaprolactone
Pd: Palladium
PDI: Polydispersity index
PDMS: Polydimethylsiloxane
PDMS@GDY@Cu: Polydimethylsiloxane@graphdiyne@Cu
g-C3N4/PDY: G-C3N4/porphyrin-based graphdiyne
PEG: Polyethylene glycol
PLGA: Polylactic acid-glycolic acid
Pt: Platinum
PTT: Photothermal therapy
QY: Quantum yield
RAP: Receptor-associated protein
Rh: Rhodium
ROS: Reactive oxygen species
Ru: Ruthenium
Ru@GDYO: Ru nanoparticles@graphdiyne oxide
S: Sulfur
S/N: Signal to noise ratio
SEM: Scanning electron microscope
S-GDY: Sandwich graphdiyne
TBAF: Tetrabutylammonium fluoride
TDNs: Tetrahedral DNA nanostructures
TdT: Terminal deoxynucleotidyl transferase
TEM: Transmission electron microscopy
THF: Tetrahydrofuran
TiO_2_: Titanium dioxide
TMB: Tetramethylbenzidine
TME: Tumor microenvironment
TTIS: Tumor-targeting iron sponge
UV–Vis: Ultraviolet–visible spectrophotometry
UV–Vis-NIR: Ultraviolet–visible-near-infrared region absorption spectroscopy
VPQDs: Violet-phosphorus quantum dots
XPS: X-ray photoelectron spectroscopy
XRD: X-ray diffraction
ZnO: Zinc oxide
ZnO@GDY NR: ZnO nanorods@graphdiyne nanosheets
1D: 1-Dimensional
2D: 2-Dimensional
3D: 3-Dimensional
α-Syn: α-Synuclein
