# Supplementary material for: Graphdiyne biomaterials: from characterization to properties and applications
Source: J Nanobiotechnology. 2025 Mar 4;23:169. doi: 10.1186/s12951-025-03227-y (PMC11881411; doi:10.1186/s12951-025-03227-y)
Supplement: Supplementary file 1 — Supplementary Material 1. [file 12951_2025_3227_MOESM1_ESM.docx]

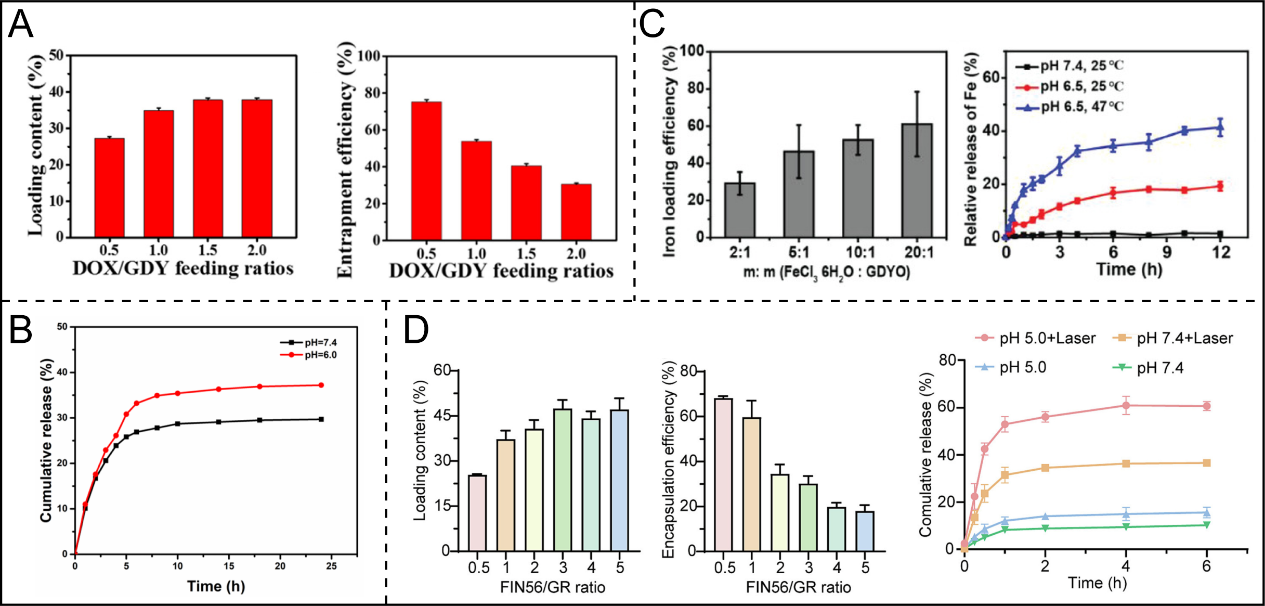


**Figure S1. Drug loading and release of GDY biomaterials.** (A) LC and EE of GDY nanodelivery platform. LC gradually raised up to 38% with feeding ratio increase, while EE decreased from 75% to 30% ^[25]^. Adapted with permission from ref. ^[25]^. Copyright 2018, American Chemical Society. (B) DOX release curve from the GDYO nanoplatform over 24 h. At pH 6.0, 37.2% of DOX was released, while at pH 7.4, 29.7% of DOX was released ^[102]^. Adapted with permission from ref. ^[102]^. Copyright 2021, John Wiley and Sons. (C) Fe content of rGDYO-loaded and Fe release curves. rGDYO loaded Fe up to a maximum of >60%. rGDYO released less than 5% of Fe at pH 7.4 and 25 ℃, and ≈20% at pH 6.5 and 25 ℃. The total Fe release increased at pH 6.5 and 47 ℃ ^[96]^. Adapted with permission from ref. ^[96]^. Copyright 2020, John Wiley and Sons. (D) LC and EE of GDY nanodelivery platform and drug release curve under laser and pH conditions. The LC gradually increased up to 47.166 ± 3.18 % and EE decreased from 68.25 ± 0.72 % to 18 ± 2.28 %. The drug release under different conditions was 10.24 ± 1.02 % (pH 7.4), 15.65 ± 1.87 % (pH 5.0), 36.62 ± 0.57 % (pH 7.4 with laser) and 60.76 ± 1.53 % (pH 5.0 with laser), respectively ^[12]^. Adapted with permission from ref. ^[12]^. Copyright 2023, Elsevier.


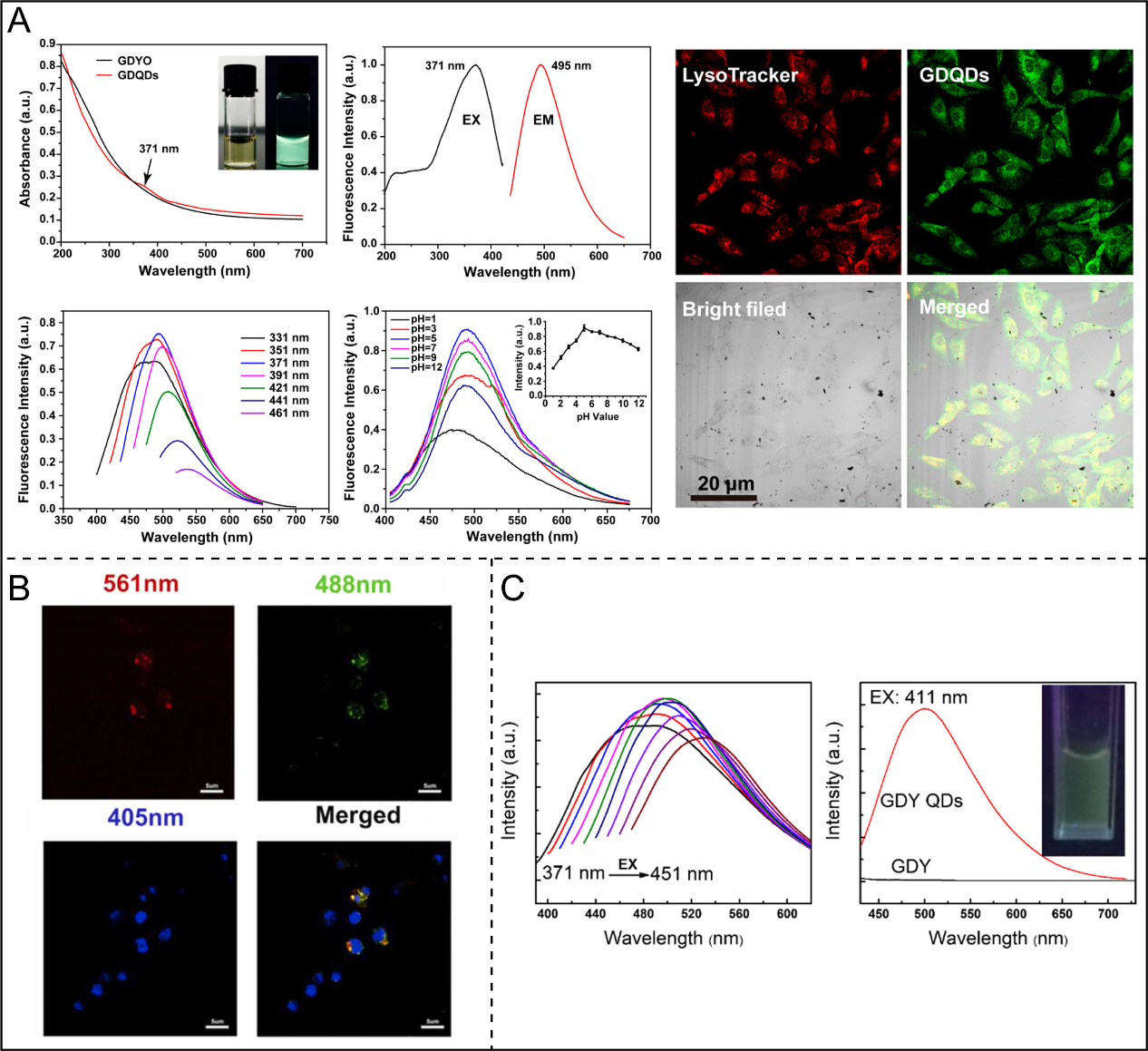


**Figure S2. Fluorescent property of GDY biomaterials.** (A) UV-Vis/fluorescence spectra and fluorescence images of GDYO and GDQDs. The GDQDs exhibited an absorption band at approximately 371 nm, with the fluorescence emission wavelength of the GDQDs demonstrating a maximum of 495 nm under excitation of 371 nm. The emission peak of the GDQDs reached its maximum intensity at pH 5. Localization of the GDQDs to the lysosome in the HUVEC cell was observed ^[13]^. Adapted with permission from ref. ^[13]^. Copyright 2019, American Chemical Society. (B) Fluorescence imaging of peritoneal fluid cells. The green signal could be derived from GDQDs ^[111]^. Adapted with permission from ref. ^[111]^. Copyright 2023, Elsevier. (C) Fluorescence spectra of GDYO QDs. The emission wavelength is red-shifted as the excitation wavelength changes from 371 nm to 451 nm, with the strongest excitation peak at 411 nm ^[112]^. Adapted with permission from ref. ^[112]^. Copyright 2022, American Chemical Society.


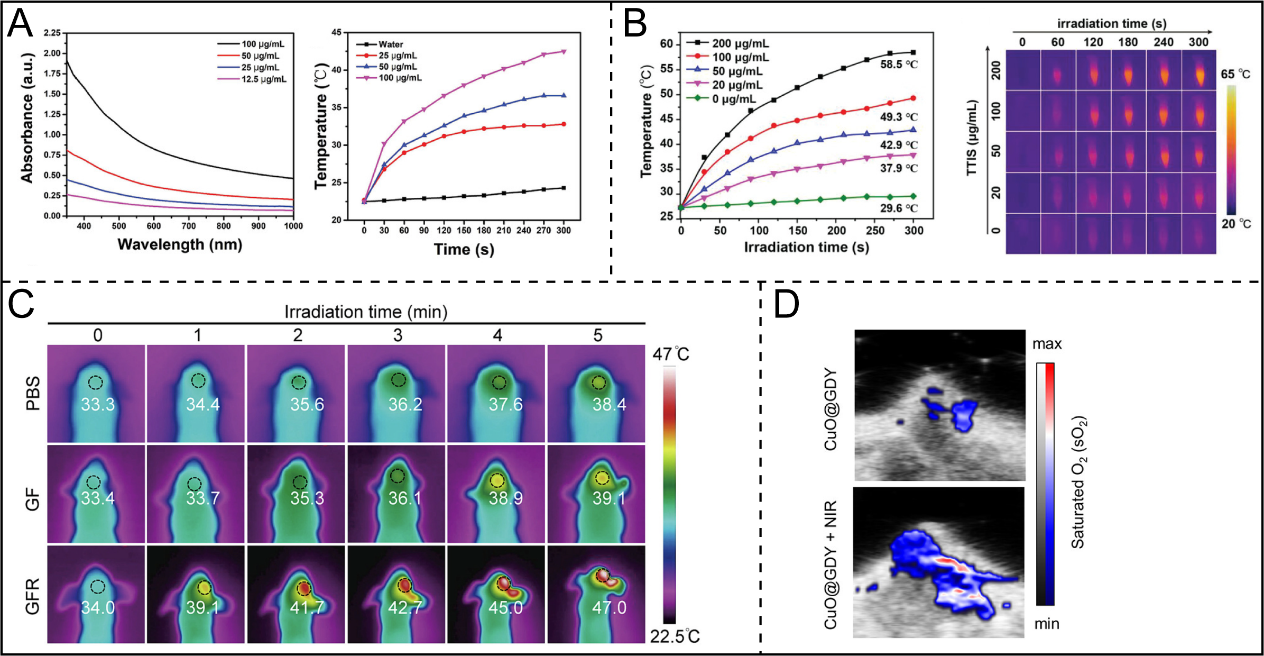


**Figure S3. Photothermal property of GDY biomaterials.** (A) UV-Vis-NIR spectra of different concentrations of GDYO dispersion and temperature change curves under NIR irradiation. The GDYO materials have absorption peaks in NIR and the temperature increase is concentration dependent ^[102]^. Adapted with permission from ref. ^[102]^. Copyright 2021, John Wiley and Sons. (B) Temperature curves and infrared images of different concentrations of GDYO dispersion under NIR irradiation. The temperature of the GDYO materials under NIR irradiation increased with respect to the concentration, and the temperature of the dispersed system with 200 µg/mL increased up to 58.5°C ^[96]^. Adapted with permission from ref. ^[96]^. Copyright 2020, John Wiley and Sons. (C) Under 808 nm (1 W/cm2) irradiation, the GDY nanoplatform (GFR) with targeted property induced a temperature increase of 13°C at the GBM site ^[12]^. Adapted with permission from ref. ^[12]^. Copyright 2023, Elsevier. (D) PAI images of saturated oxygen levels at the tumor site after treatment with GDY material and NIR laser ^[124]^. Adapted with permission from ref. ^[124]^. Copyright 2022, American Chemical Society.


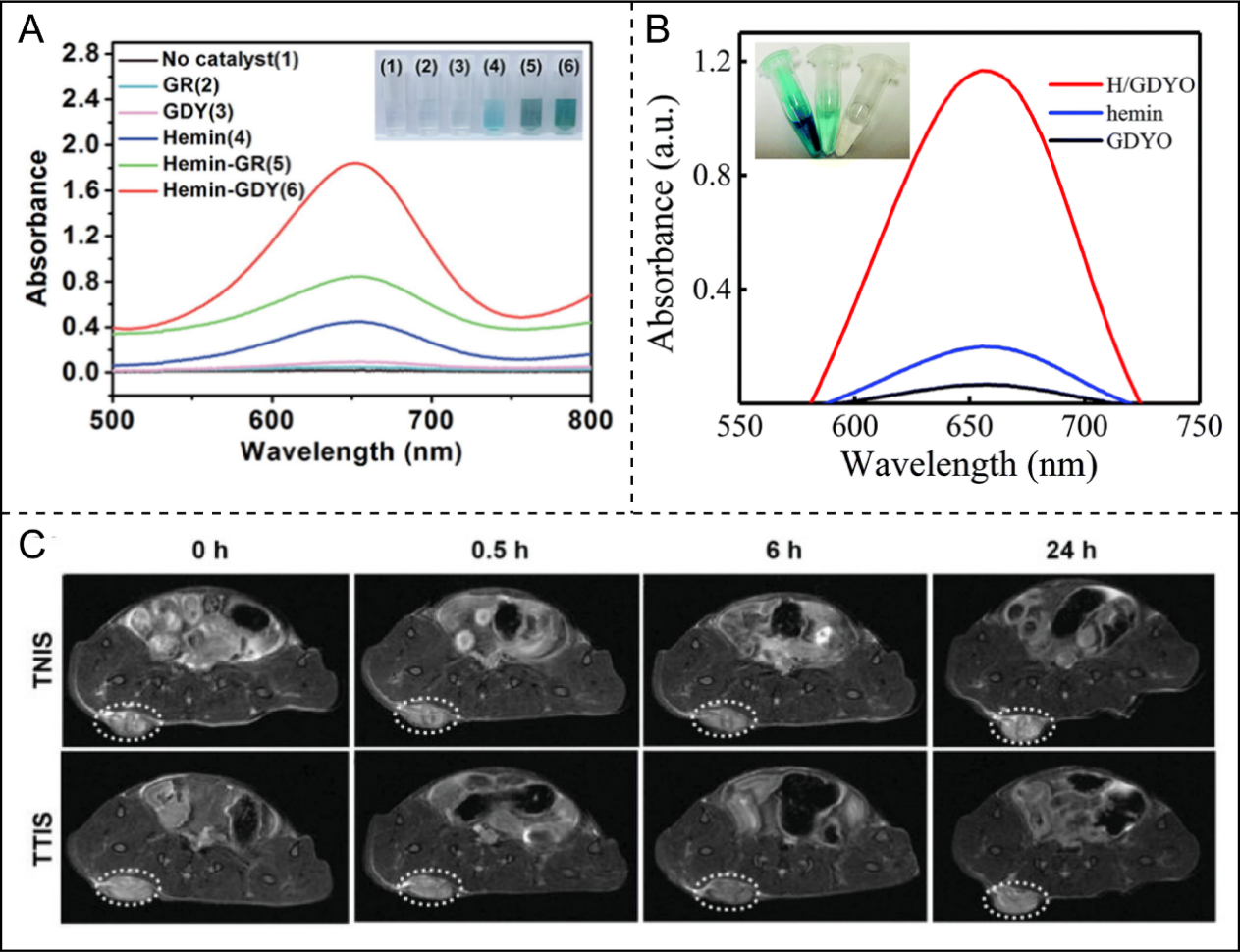


**Figure S4. Catalytic and MRI properties of GDY biomaterials.** (A, B) Mixtures of H_2_O_2_ and TMB incubated with Hemin-GDY and H/GDYO exhibiting peroxidase properties yielded blue products with significant absorption peaks at 620-650 nm ^[24, 128]^. Adapted with permission from ref. ^[24]^. Copyright 2022, Elsevier, and ref. ^[128]^. Copyright 2021, Royal Society of Chemistry. (C) TTIS showed that the T_2_-weighted MRI image weakens with increasing injection time, indicating that TTIS can be used as an effective contrast agent for T_2_-weighted MRI ^[96]^. Adapted with permission from ref. ^[96]^. Copyright 2020, John Wiley and Sons.


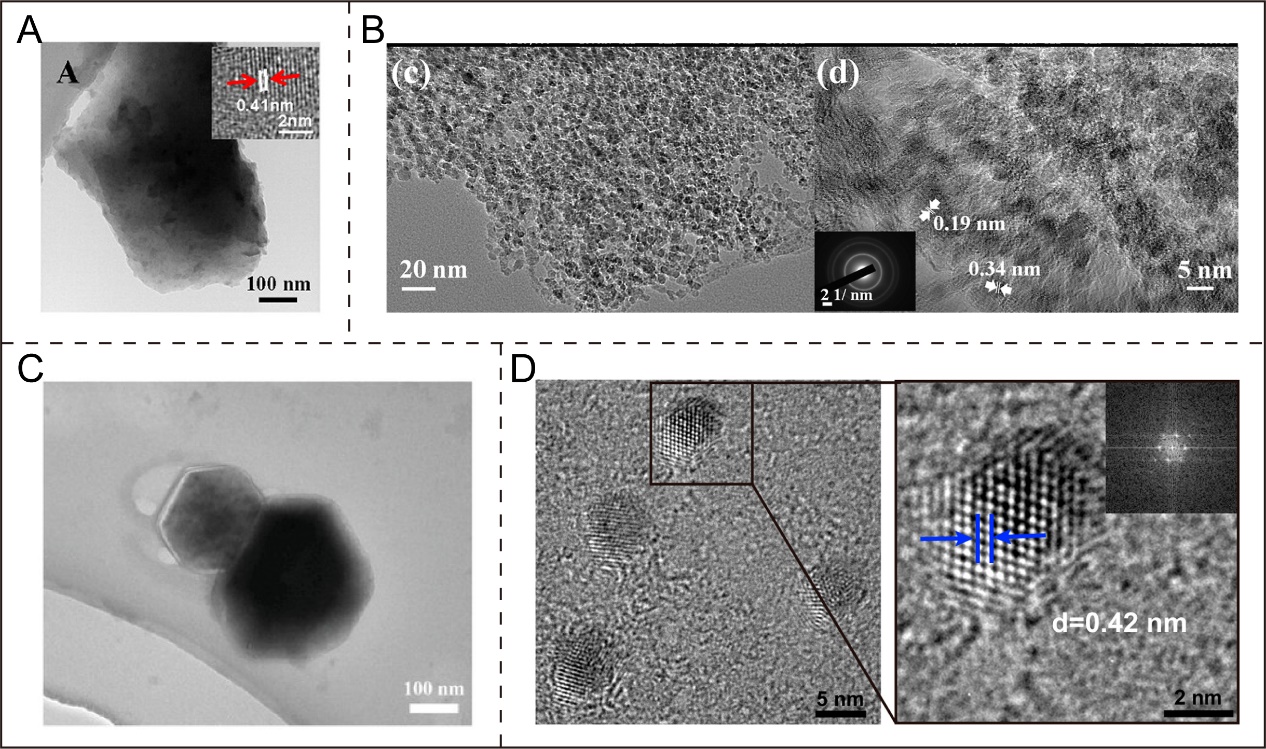


**Figure S5.TEM characterization of GDY biomaterials.** (A) TEM images of B-doped and ketone-rich GDY nanoenzymes. the GDY material is bulk structured with a lattice spacing of 0.41 nm ^[26]^. Adapted with permission from ref. ^[26]^. Copyright 2022, American Chemical Society. (B) TEM images of nanodiamond/GDY nanoisomers. The prepared nanoparticles have a spherical structure with a size of about 5 nm. The lattice spacing of nanodiamond and GDY is 0.19 nm and 0.34 nm, respectively ^[30]^. Adapted with permission from ref. ^[30]^. Copyright 2021, Elsevier. (C) TEM image of the prepared GDY nanodelivery system ^[102]^. Adapted with permission from ref. ^[102]^. Copyright 2021, John Wiley and Sons. (D) High-resolution TEM image of GDYO QDs. the particle size of GDYO QDs is about 5 nm and the lattice spacing is 0.42 nm ^[13]^. Adapted with permission from ref. ^[13]^. Copyright 2019, American Chemical Society.


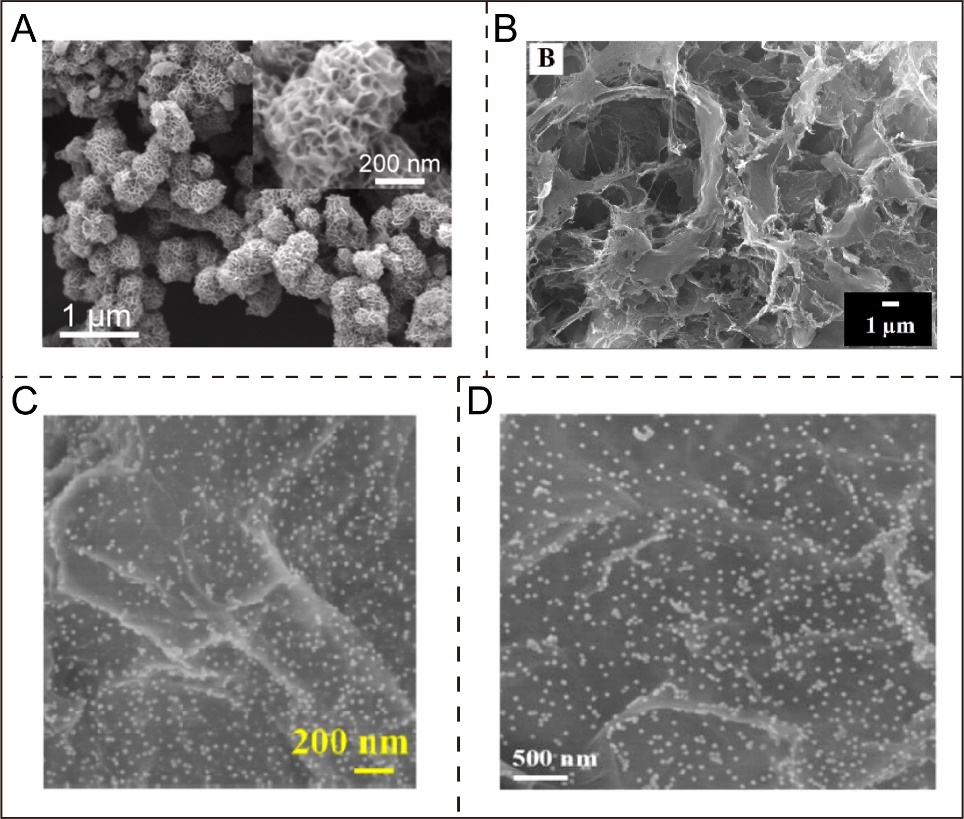


**Figure S6.SEM characterization of GDY biomaterials.** (A) SEM image of the GDY sensor ^[27]^. Adapted with permission from ref. ^[27]^. Copyright 2022, Elsevier. (B) SEM images of N-doped GDY nanosheets ^[31]^. Adapted with permission from ref. ^[31]^. Copyright 2022, Elsevier. (C) SEM image of AuNPs distribution on the surface of GDY nanosheets ^[147]^. Adapted with permission from ref. ^[147]^. Copyright 2021, American Chemical Society. (D) SEM image of the distribution of AuNPs on the GDY surface ^[148]^. Adapted with permission from ref. ^[148]^. Copyright 2022, American Chemical Society.


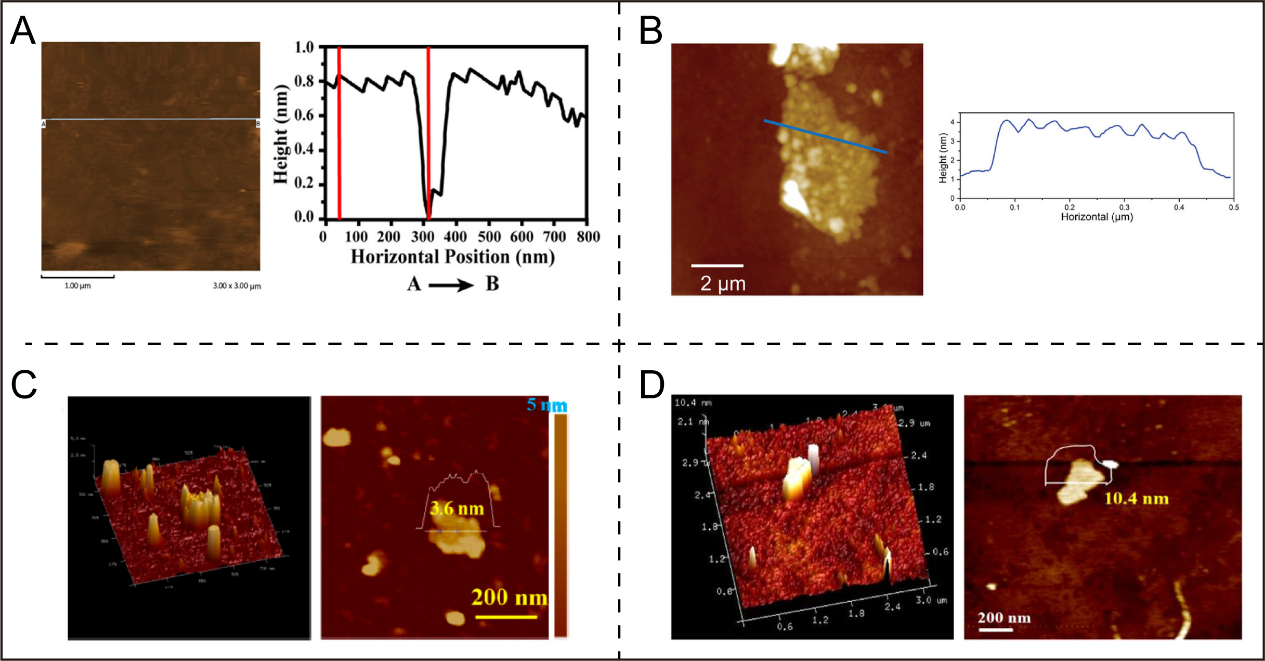


**Figure S7. AFM characterization of GDY biomaterials.** (A) AFM images and thickness analysis of GDY nanosheets. The thickness of the prepared GDY nanosheets was 0.9 nm ^[28]^. Adapted with permission from ref. ^[28]^. Copyright 2019, American Chemical Society. (B) AFM images and thickness analysis of GDY film. The thickness of GDY film is about 3-4 nm ^[155]^. Adapted with permission from ref. ^[155]^. The reproduced content is open access. (C) AFM images and thickness analysis of nanogold-modified GDY nanosheets. the thickness of GDY nanosheets is 3.6 nm ^[147]^. Adapted with permission from ref. ^[147]^. Copyright 2021, American Chemical Society. (D) AFM images and thickness analysis of the prepared GDY sandwich structure. The GDY thickness of the sandwich structure is 10.4 nm ^[148]^. Adapted with permission from ref. ^[148]^. Copyright 2022, American Chemical Society.


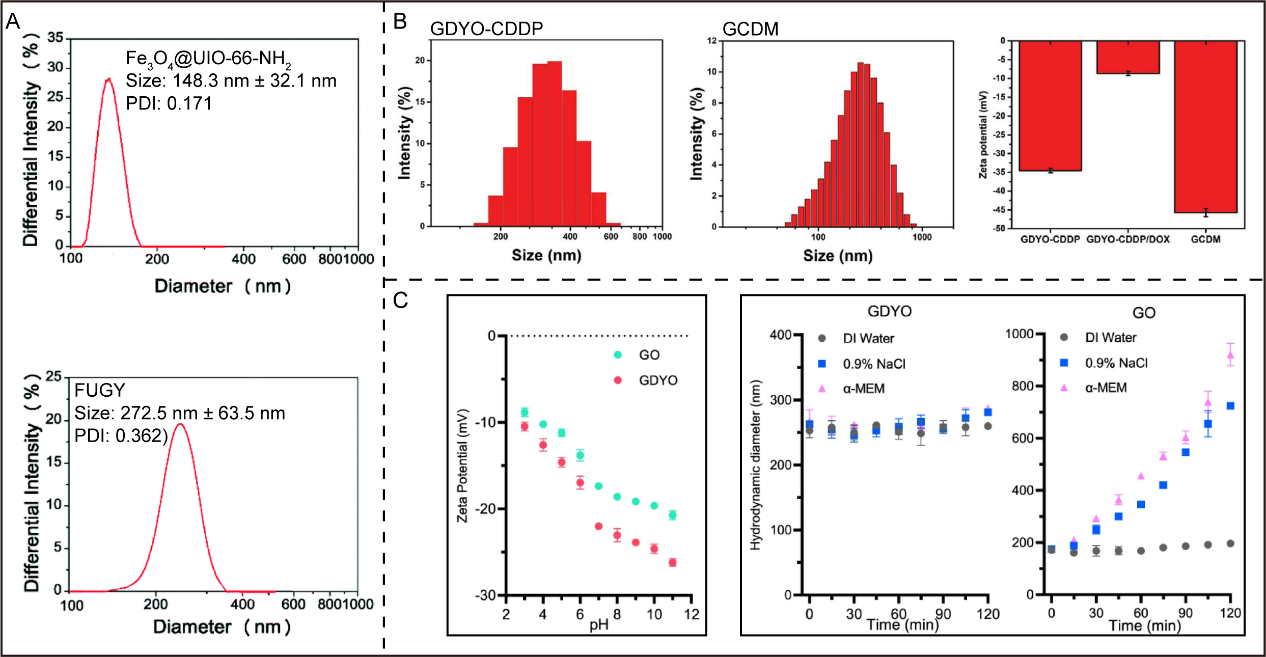


**Figure S8. Size, dispersion and zeta potential characterization of GDY biomaterials.** (A) Hydrodynamic radius and PDI of Fe_3_O_4_@UIO-66-NH_2_ and FUGY. The wrapping effect of the GDY layer increased the size and PDI of the nanomaterials ^[165]^. Adapted with permission from ref. ^[165]^. Copyright 2019, Royal Society of Chemistry. (B) Size distributions of GDYO-CDDP and GCDM analyzed by TEM, and zeta potentials of GDYO-CDDP, GDYO-CDDP/DOX, and GCDM. The average hydrodynamic radius were 342 nm (GDYO-CDDP) and 250 nm (GCDM), and the zeta potentials were -34.5 mV (GDYO-CDDP/DOX) and -8.7 mV (GDYO-CDDP/DOX), respectively ^[102]^. Adapted with permission from ref. ^[102]^. Copyright 2021, John Wiley and Sons. (C) Zeta potential of GO and GDYO nanosheets at different pH values, and the hydrodynamic diameter of GO and GDYO nanosheets in different dispersion solutions ^[166]^. Adapted with permission from ref. ^[166]^. The reproduced content is open access.


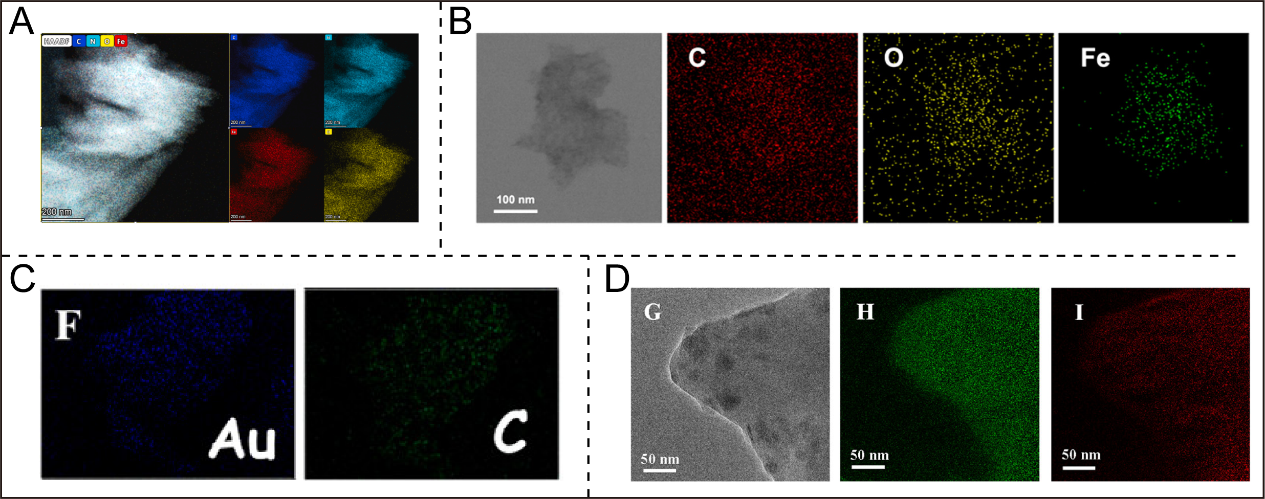


**Figure S9. EDS characterization of GDY biomaterials.** (A) TEM-EDS images of GDY/Hemin chloride complexes. The EDS results show the distribution of N, O, and Fe elements on the material ^[29]^. Adapted with permission from ref. ^[29]^. Copyright 2022, Elsevier. (B) Distribution images of O and Fe elements on GDY nanosheets obtained by EDS analysis ^[10]^. Adapted with permission from ref. ^[10]^. Copyright 2019, American Chemical Society. (C) Image of Au and C distribution on AuNPs /GDY modified electrode ^[172]^. Adapted with permission from ref. ^[172]^. Copyright 2021, American Chemical Society. (D) Distribution of B on B-doped GDY nano-enzyme materials. Green refers to element B and red to element C ^[26]^. Adapted with permission from ref. ^[26]^. Copyright 2022, American Chemical Society.


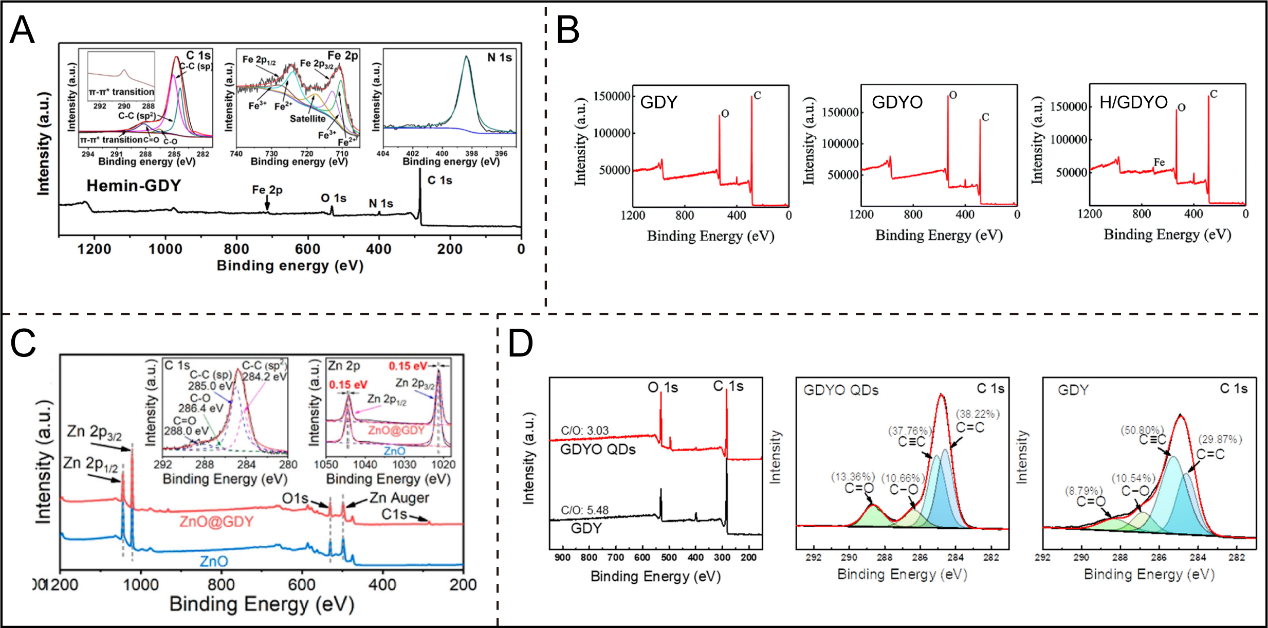


**Figure S10. XPS characterization of GDY biomaterials.** (A) XPS mapping of Hemin-GDY. The results showed that the oxidation states of Fe in Hemin-GDY were Fe^3+^ and Fe^2+^, and the states of C were C=O, C-O, C≡C(*sp*) and C=C(*sp^2^*) ^[24]^. Adapted with permission from ref. ^[24]^. Copyright 2022, Elsevier. (B) XPS spectra of GDY, GDYO and Hemin/GDYO (H/GDYO). The Fe characteristic peaks in the H/GDYO spectra proved that Hemin was loaded onto GDYO ^[128]^. Adapted with permission from ref. ^[128]^. Copyright 2021, Royal Society of Chemistry. (C) XPS spectra of ZnO@GDY nanosheets. The binding energies of Zn 2p_3/2_ and Zn 2p_1/2_ in the XPS spectra of ZnO@GDY are 0.15 eV lower than those in ZnO ^[180]^. Adapted with permission from ref. ^[180]^. Copyright 2022, American Chemical Society. (D) XPS spectra of GDY and GDYO QDs. the C/O ratios of GDY and GDYO ODs are 5.48 and 3.03, respectively ^[112]^. Adapted with permission from ref. ^[112]^. Copyright 2022, American Chemical Society.


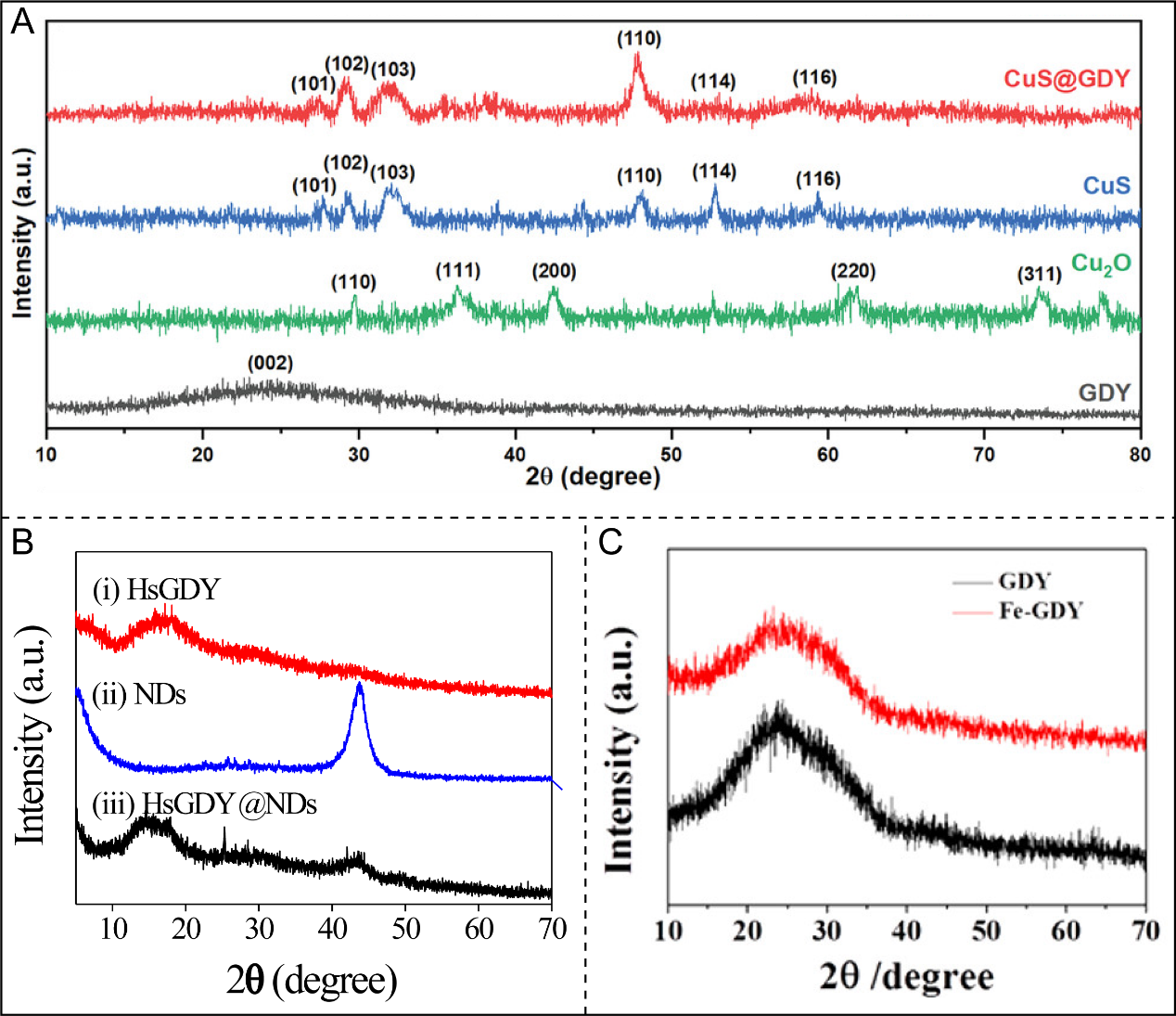


**Figure S11. XRD characterization of GDY biomaterials.** (A) XRD spectra of CuS@GDY cubes. The diffraction peaks of hexagonal CuS at 28.0°, 29.3°, 31.7° and 47.9° did not appear in the original GDY ^[191]^. Adapted with permission from ref. ^[191]^. Copyright 2022, John Wiley and Sons. (B) XRD spectra of HsGDY isomers. The diffraction pattern of the HsGDY@ND hybrid showed a characteristic diamond plane peak at 43.8° ^[30]^. Adapted with permission from ref. ^[30]^. Copyright 2021, Elsevier. (C) XRD spectra of Fe-GDY. The Fe-GDY and GDY spectra shared similar characteristic peak positions and did not show to specific peaks from the Fe lattice plane ^[10]^. Adapted with permission from ref. ^[10]^. Copyright 2019, American Chemical Society.


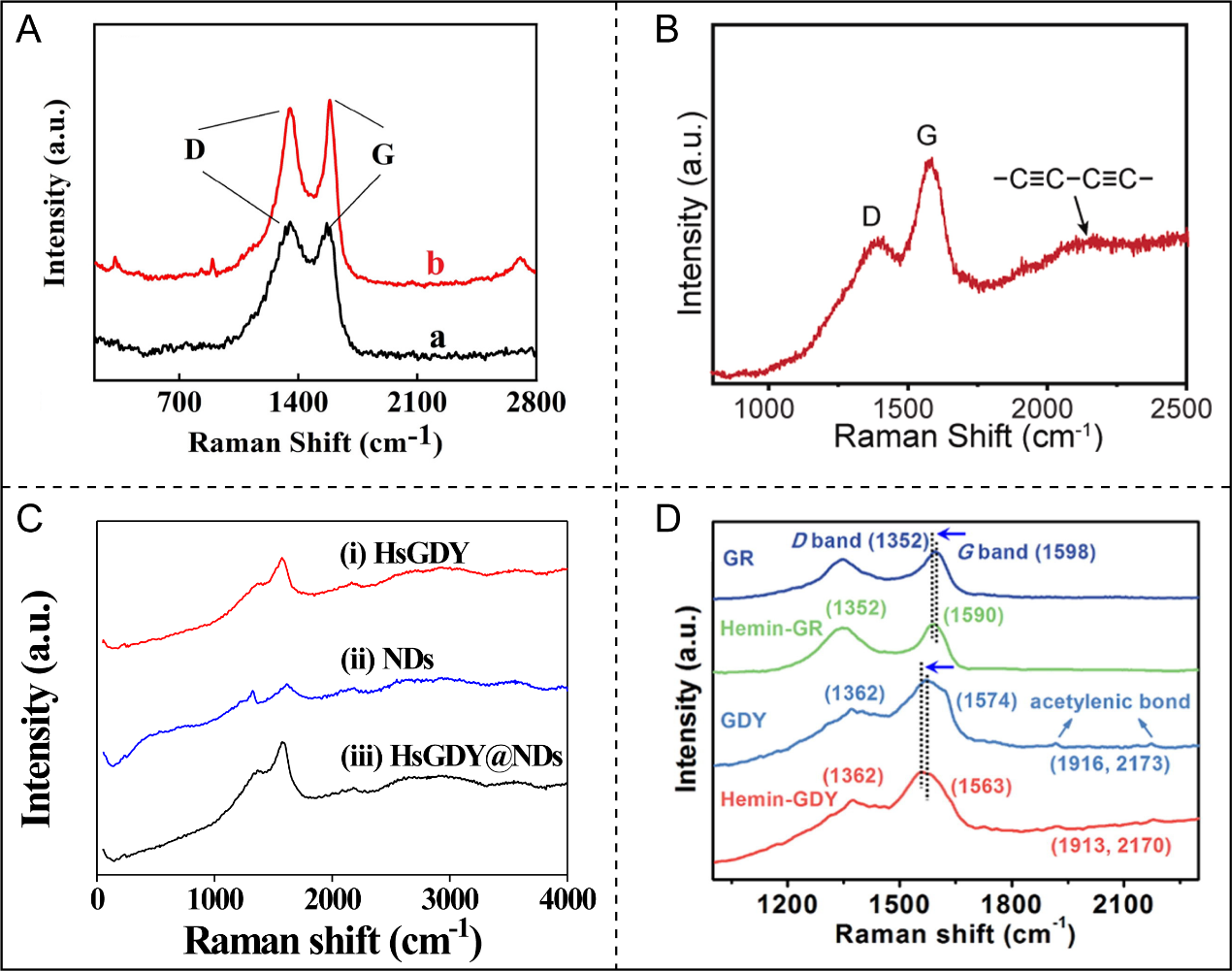


**Figure S12. Raman characterization of GDY biomaterials.** (A) Raman plots of GDY and N-doped GDY. Black indicates GDY. both data show characteristic peaks at 1369 cm^-1^ and 1571 cm^-1^ ^[31]^. Adapted with permission from ref. ^[31]^. Copyright 2022, Elsevier. (B) Raman spectra of GDY biomaterials. The data showed two characteristic peaks at 1388 cm^-1^ and 1573 cm^-1^ ^[27]^. Adapted with permission from ref. ^[27]^. Copyright 2022, Elsevier. (C) Raman spectra of three GDY materials. Two peaks at 1370 cm^-1^ and 1597 cm^-1^ were detected in the Raman data for all three materials ^[30]^. Adapted with permission from ref. ^[30]^. Copyright 2021, Elsevier. (D) Raman spectra of Hemin-modified GDY materials. Hemin modification broadened the D band and G band of GDY biomaterials ^[24]^. Adapted with permission from ref. ^[24]^. Copyright 2022, Elsevier.


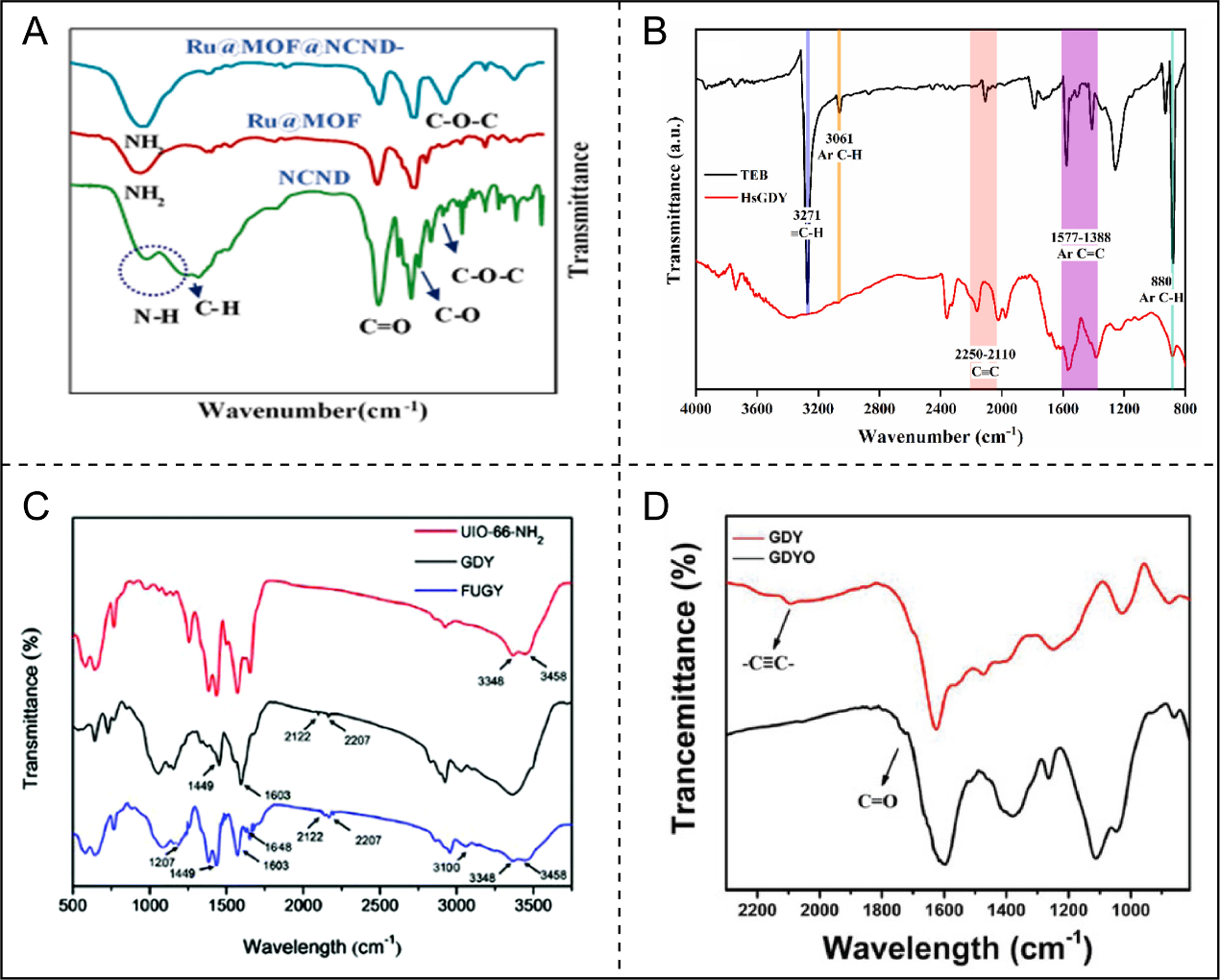


**Figure S13. FTIR characterization of GDY biomaterials.** (A) FTIR spectra of MOF-modified GDY nanodots. Peaks at 3422.29 cm^-1^ and 3454.36 cm^-1^, corresponding to NH_2_ groups in the MOF ^[32]^. Adapted with permission from ref. ^[32]^. Copyright 2022, Elsevier. (B) FTIR spectra of Triethynylbenzene and HsGDY. The characteristic peak of Triethynylbenzene at 3271 cm^-1^ was not detected in HsGDY ^[214]^. Adapted with permission from ref. ^[214]^. Copyright 2022, Elsevier. (C) FTIR spectra of FUGY. The results for the GDY and FUGY materials showed benzene ring stretching vibrations at 1449 cm^-1^ and 1603 cm^-1^, and C≡C stretching vibrations at 2122 cm^-1^ and 2207 cm^-1^ ^[165]^. Adapted with permission from ref. ^[165]^. Copyright 2019, Royal Society of Chemistry. (D) FTIR spectra of GDY and GDYO. GDYO data showed the presence of the characteristic C=O absorption peak at 1720 cm^-1^ ^[102]^. Adapted with permission from ref. ^[102]^. Copyright 2021, John Wiley and Sons.


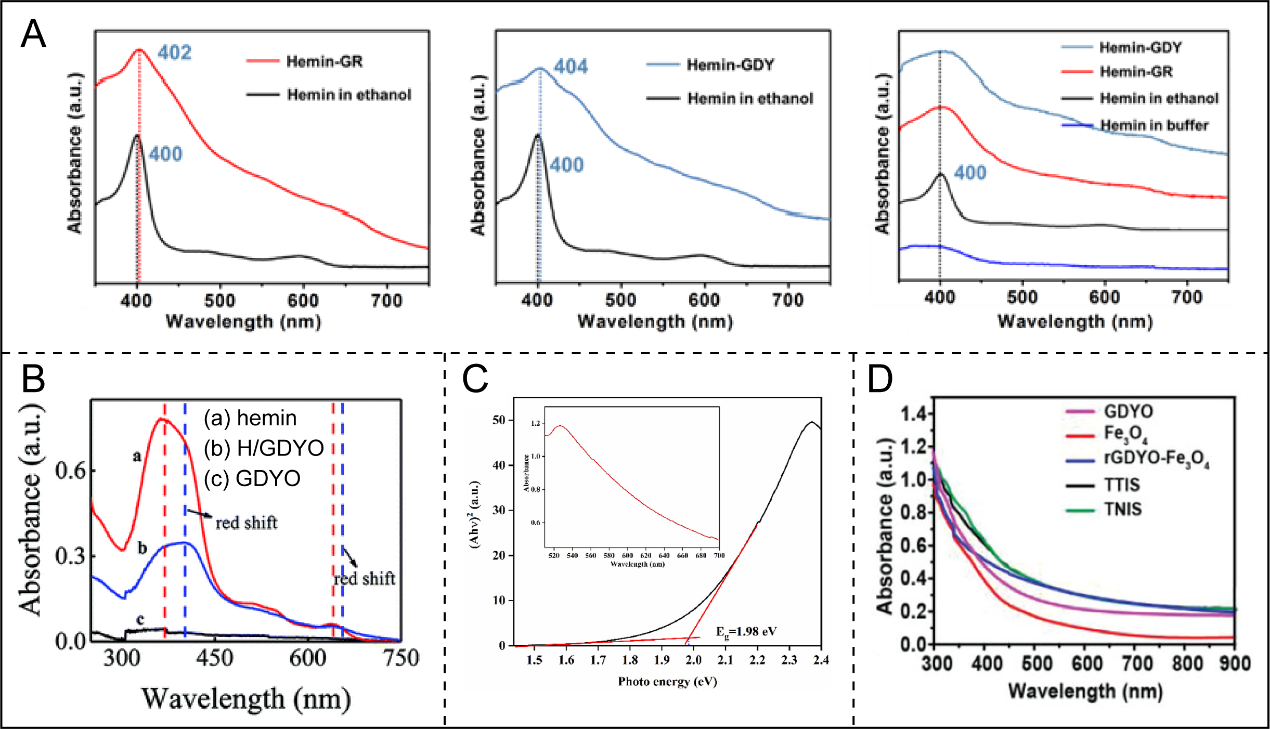


**Figure S14. UV-Vis characterization of GDY biomaterials.** (A) UV-Vis spectra of Hemin-loaded Graphene (Hemin-GR) and Hemin-GDY in different dispersion systems. Absorption peaks characteristic of free Hemin were detected in both Hemin-GR and Hemin-GDY. However, the Q bands of Hemin in Hemin-GDY material underwent a 4 nm shift (larger than its 2 nm shift in Hemin-GR) ^[24]^. Adapted with permission from ref. ^[24]^. Copyright 2022, Elsevier. (B) UV-Vis spectra of H/GDYO. The data showed that the characteristic Hemin peaks in H/GDYO were red-shifted to 400 nm and 655 nm, indicating an increase in the conjugated system ^[128]^. Adapted with permission from ref. ^[128]^. Copyright 2021, Royal Society of Chemistry. (C) Tauc plot obtained from UV-Vis spectra of HsGDY, inset: UV–Vis absorption spectrum of HsGDY. The data showed a broadband centered at 548 nm with a bandgap of 1.98 eV ^[214]^. Adapted with permission from ref. ^[214]^. Copyright 2022, Elsevier. (D) UV-Vis spectra of GDYO-Fe_3_O_4_ nanoplatforms. The absorbance of GDYO-Fe3O4 in NIR was significantly enhanced ^[96]^. Adapted with permission from ref. ^[96]^. Copyright 2020, John Wiley and Sons.


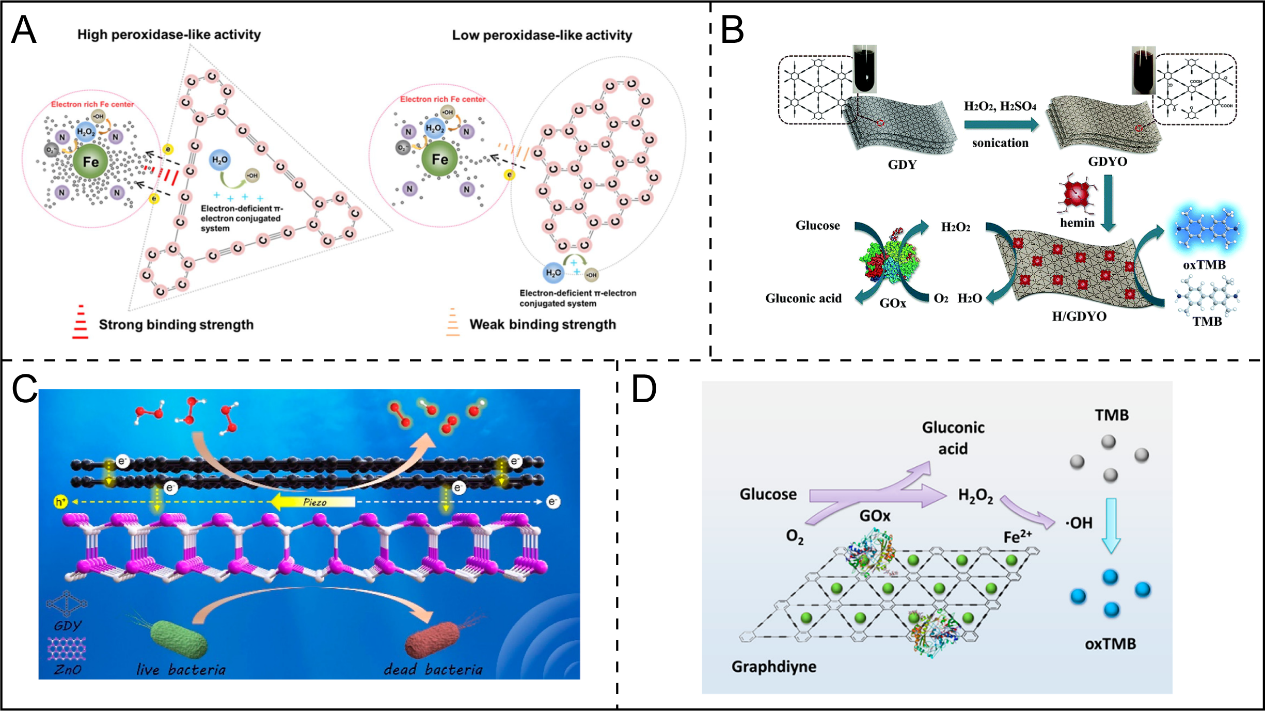


**Figure S15. GDY biomaterials in catalysis.** (A) Schematic diagram of the immobilization of Hemin on the surface of GDY to catalyze the decomposition of H_2_O_2_ to produce hydroxyl radicals and superoxide anions ^[24]^. Adapted with permission from ref. ^[24]^. Copyright 2022, Elsevier. (B) Schematic diagram of H/GDYO prepared by immobilizing Hemin on the surface of GDYO for detecting H_2_O_2_ and glucose ^[128]^. Adapted with permission from ref. ^[128]^. Copyright 2021, Royal Society of Chemistry. (C) Schematic diagram of the antimicrobial function of ZnO@GDY NR under ultrasound irradiation by promoting H_2_O_2_ decomposition and ROS generation ^[180]^. Adapted with permission from ref. ^[180]^. Copyright 2022, American Chemical Society. (D) Schematic diagram of Fe-GDY/GOx prepared by immobilizing Fe^2+^ and GOx on GDY nanosheets for one-step blood glucose detection ^[10]^. Adapted with permission from ref. ^[10]^. Copyright 2019, American Chemical Society.


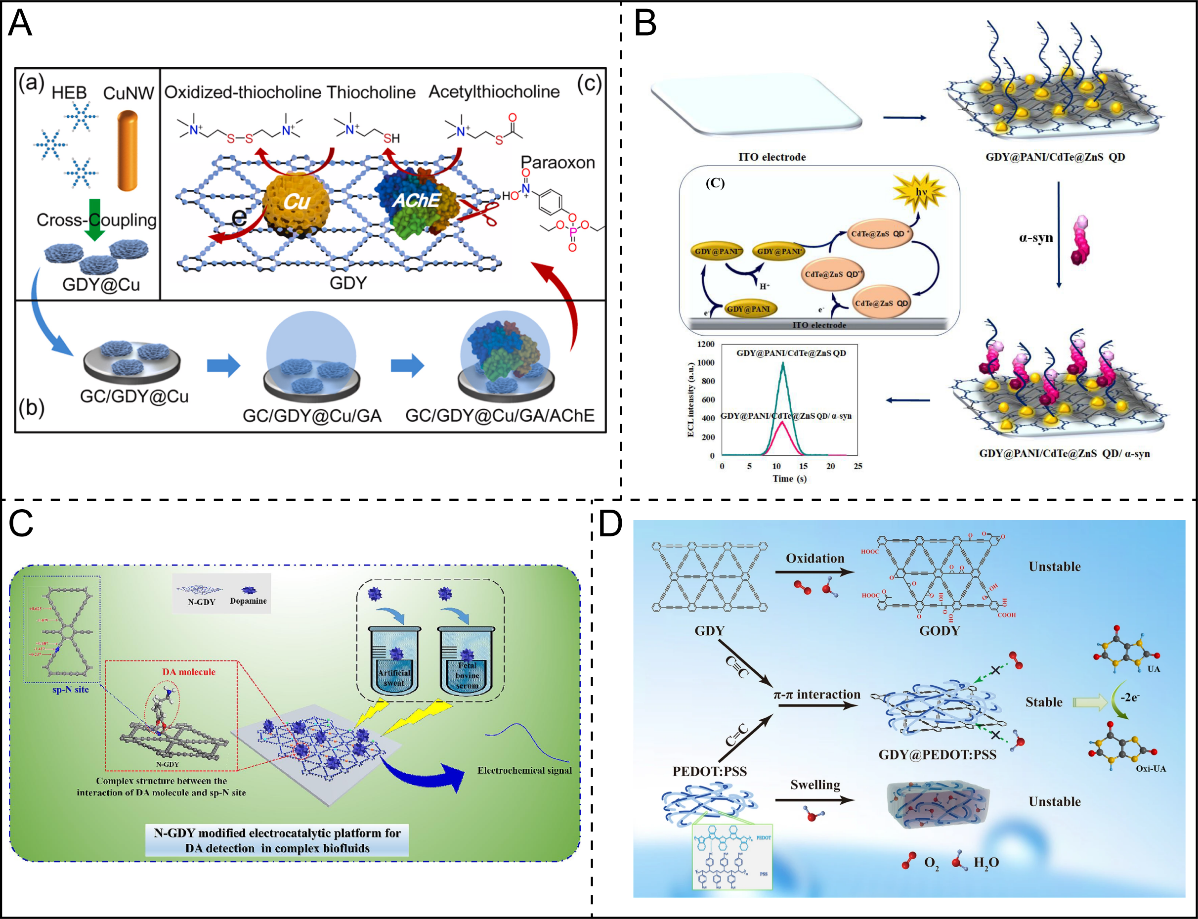


**Figure S16. Molecular detection applications of GDY biomaterials.** (A) Schematic diagram of Cu@GDY preparation process and detection of organophosphorus pesticides ^[27]^. Adapted with permission from ref. ^[27]^. Copyright 2022, Elsevier. (B) Schematic diagram of the GDY@PANI/CdTe@ZnS QD sensing platform prepared using N-doped GDY@PANI and CdTe@ZnS QDs for α-Syn detection ^[238]^. Adapted with permission from ref. ^[238]^. Copyright 2024, John Wiley and Sons. (C) Schematic of the highly selective and sensitive detection of dopamine using N-GDY ^[242]^. Adapted with permission from ref. ^[242]^. Copyright 2023, Elsevier. (D) Schematic of GDY@PEDOT:PSS heterostructure for detection of uric acid ^[243]^. Adapted with permission from ref. ^[243]^. Copyright 2024, Springer Nature.


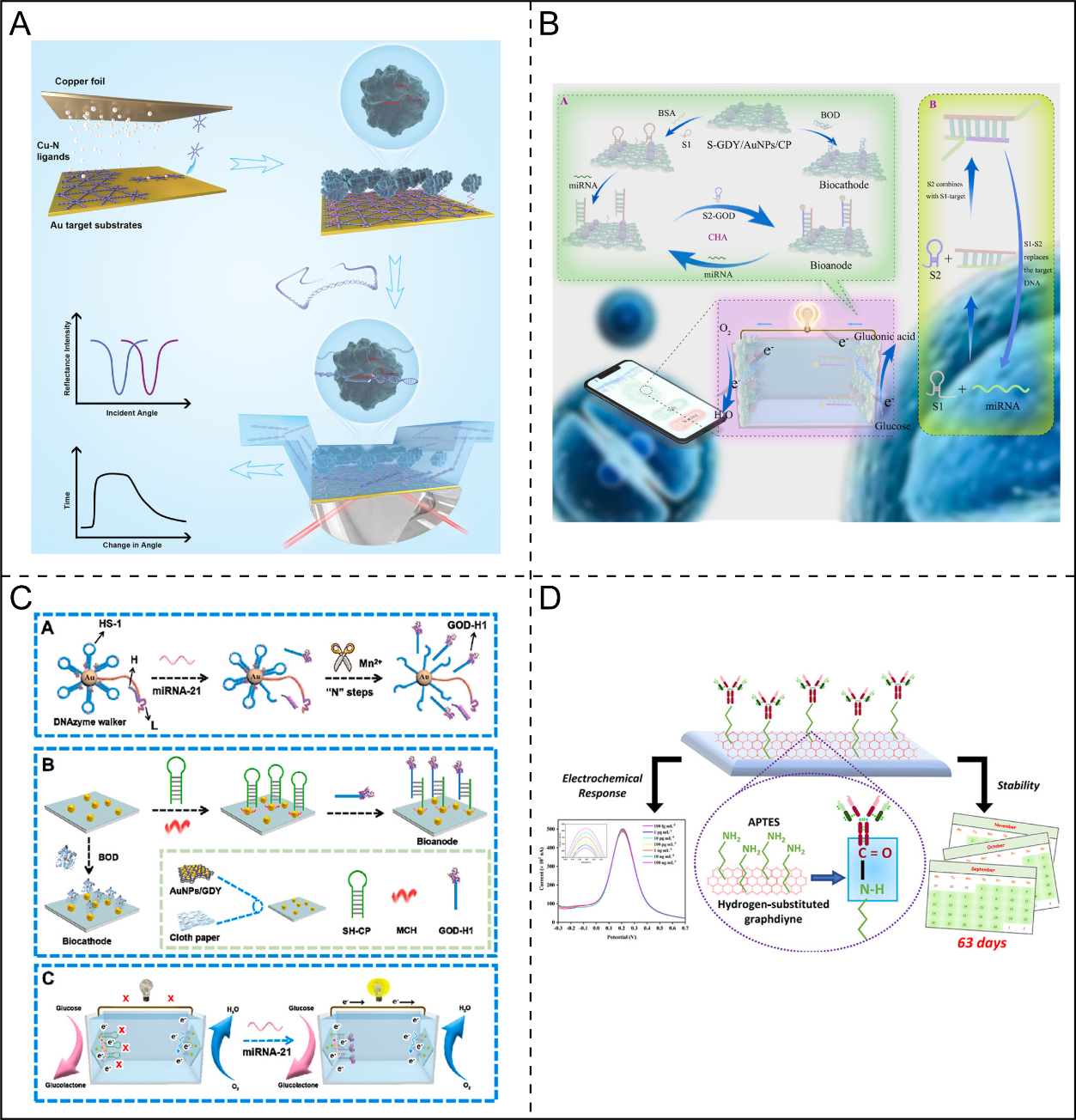


**Figure S17. Nucleic acid detection applications of GDY biomaterials.** (A) Schematic diagram of the application of GDY film with dCas9 to prepare a CRISPR-SPR-Chip sensor for recognizing and detecting genomic DNA ^[155]^. Adapted with permission from ref. ^[155]^. The reproduced content is open access. (B) Schematic diagram of the preparation of electrical biosensor using an EBFC containing S-GDY inside to detect the tumor marker miRNA-141 ^[248]^. Adapted with permission from ref. ^[248]^. Copyright 2023, Elsevier. (C) Schematic diagram of the self-powered biosensor integrating DNAzyme walker and AuNPs/GDY for miRNA-21 detection ^[251]^. Adapted with permission from ref. ^[251]^. Copyright 2023, Elsevier. (D) Schematic diagram of HsGDY detection of the liver cancer marker ANXA2 ^[260]^. Adapted with permission from ref. ^[260]^. Copyright 2023, American Chemical Society.


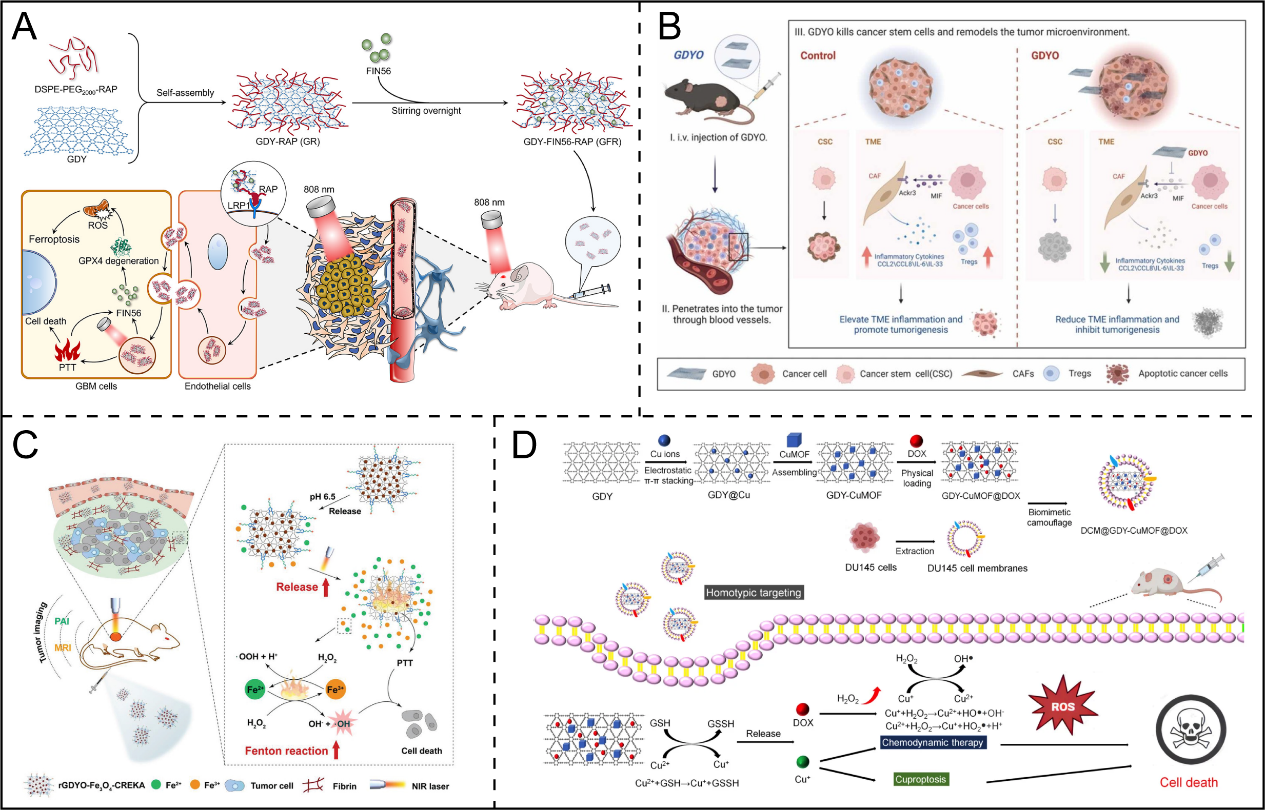


**Figure S18. Antitumor applications of GDY biomaterials.** (A) Schematic diagram of GDY nanoplatform loading FIN56 for GBM therapy via ferroptosis and PTT ^[12]^. Adapted with permission from ref. ^[12]^. Copyright 2023, Elsevier. (B) GDYO reduced the expression of inflammatory cytokines, decreased Treg cells and tumor stem cells in the TME, and ultimately inhibited lymphoma growth ^[262]^. Adapted with permission from ref. ^[262]^. Copyright 2022, Elsevier. (C) Tumor-targeting nanocomposites prepared using GDY facilitated the release of Fe under PTT and enhanced the efficiency of the Fenton reaction, thereby enabling PTT and Fenton reaction-mediated tumor combination therapy ^[96]^. Adapted with permission from ref. ^[96]^. Copyright 2020, John Wiley and Sons. (D) Schematic diagram of the DCM@GDY-CuMOF@DOX nanoplatform for prostate cancer cuproptosis therapy ^[267]^. Adapted with permission from ref. ^[267]^. The reproduced content is open access.


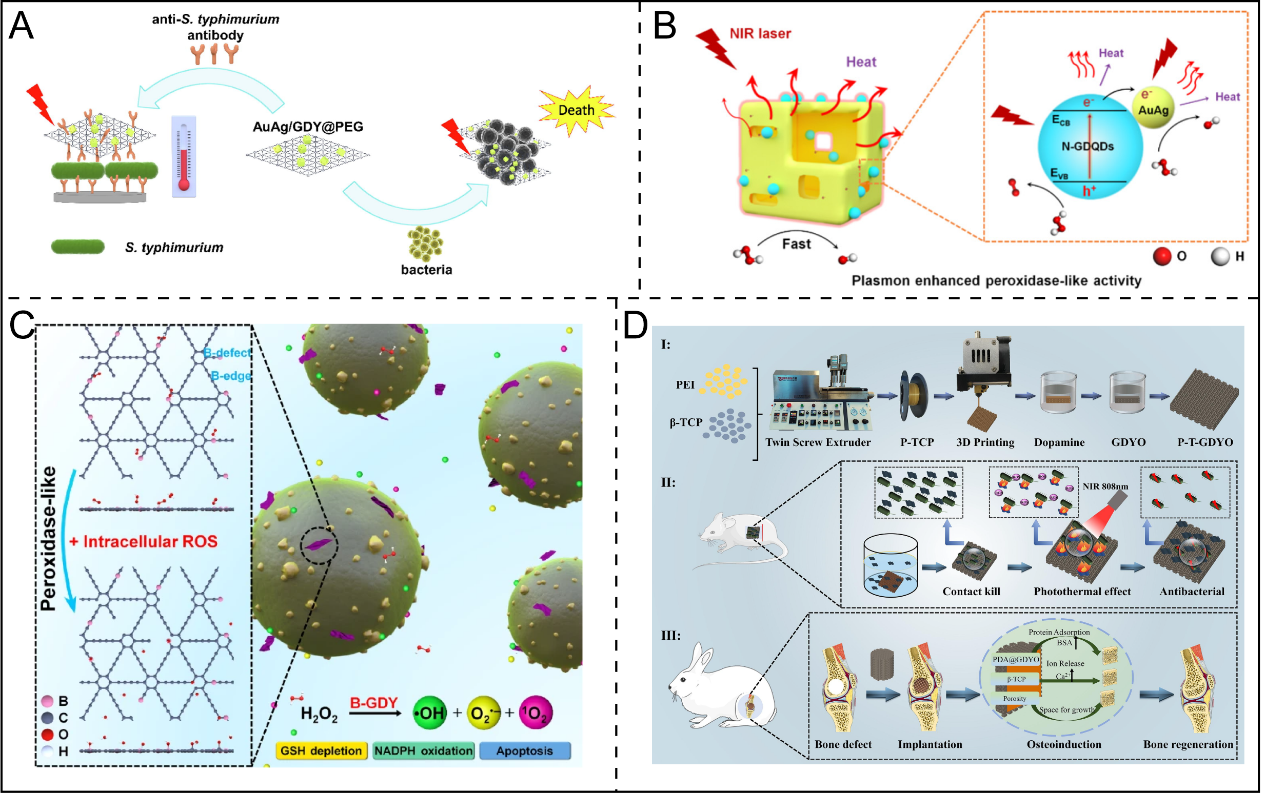


**Figure S19. Antimicrobial applications of GDY biomaterials.** (A) AuAg/GDY@PEG composites were prepared using AuAg and GDY to achieve antimicrobial activity under laser irradiation ^[272]^. Adapted with permission from ref. ^[272]^. Copyright 2022, Elsevier. (B) N-GDQDs loading on AuAg nanocages to prepare hollow cube-like N- GDQDs/AuAg nanocages to achieve antimicrobial property ^[273]^. Adapted with permission from ref. ^[273]^. Copyright 2022, American Chemical Society. (C) B-doped GDY nanosheets for bactericidal applications by promoting H_2_O_2_ decomposition to generate ROS ^[275]^. Adapted with permission from ref. ^[275]^. Copyright 2021, Springer Nature. (D) 3D printed osteogenic scaffolds loaded with GDYO realized antimicrobial effect under NIR laser irradiation. Bone regeneration was achieved after implantation of the scaffold in the bone defect model ^[271]^. Adapted with permission from ref. ^[271]^. The reproduced content is open access.


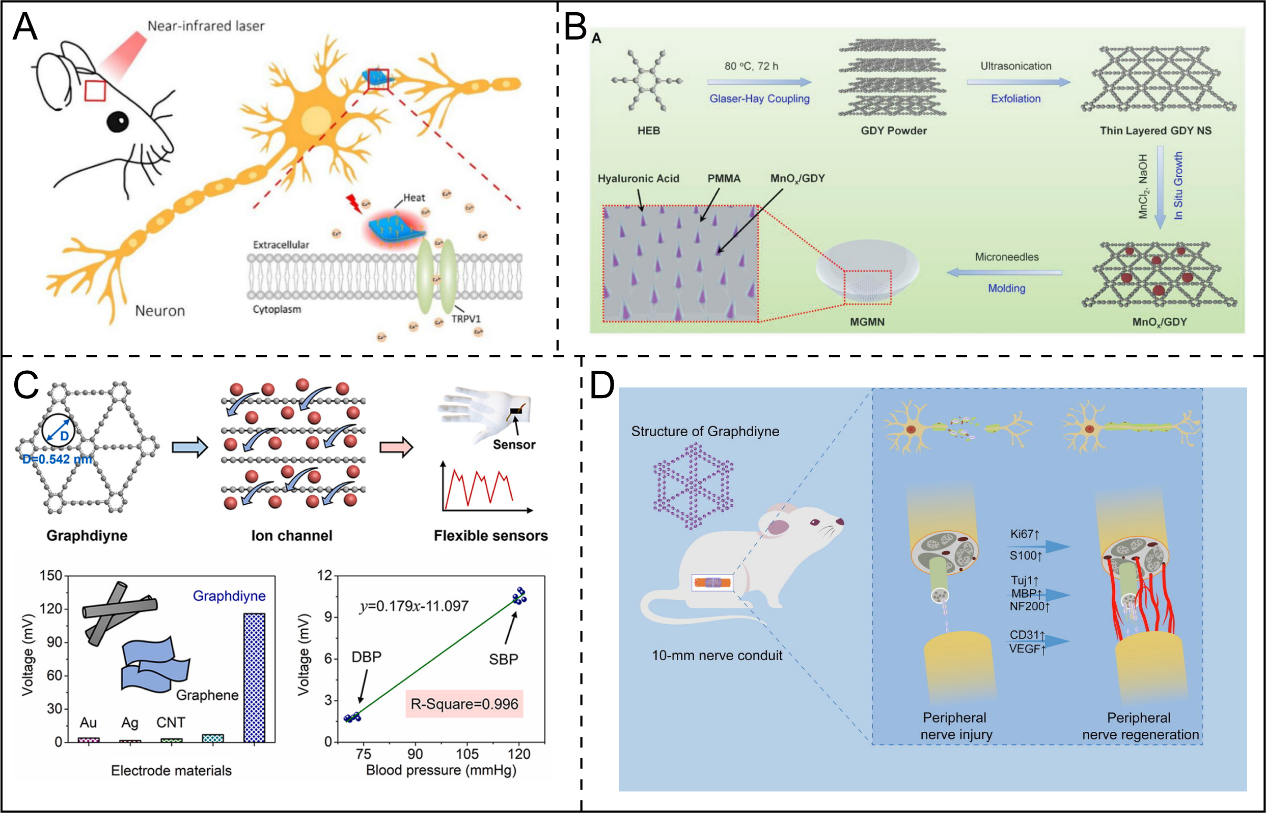


**Figure S20.** **Applications of GDY biomaterials in other biomedical research.** (A) PEG-modified GDY nanosensor specifically targeting TRPV1, which was activated under NIR, leading to cellular release of neurotransmitters and modulation of nerve firing ^[282]^. Adapted with permission from ref. ^[282]^. Copyright 2024, American Chemical Society. (B) Schematic diagram of loading MnOx/GDY nanosheets into hyaluronic acid and poly (methyl methacrylate)-based ophthalmic microneedles ^[284]^. Adapted with permission from ref. ^[284]^. Copyright 2023, John Wiley and Sons. (C) Schematic diagram of a flexible sensor prepared using GDY for precise patch detection of blood pressure ^[287]^. Adapted with permission from ref. ^[287]^. Copyright 2024, Elsevier. (D) Schematic diagram of GDY-loaded polycaprolactone composite scaffolds for promoting peripheral nerve regeneration ^[290]^. Adapted with permission from ref. ^[290]^. Copyright 2023, Elsevier.
